# Informing Decision‐Making About Caesarean Birth: A Delphi Study to Develop a Core Information Set

**DOI:** 10.1111/1471-0528.18269

**Published:** 2025-07-08

**Authors:** Carol Kingdon, Ben Greenfield, Mahmoud Aljubeh, Eve Bunni, Alexandra Hunt, Vicky Bradley, Caroline Cunningham, Siobhan Holt, Andrew Demetri, Christy Burden, Joanna Ficquet, Elena Otero‐Romero, William Parry Smith, Mairead Black, Fiona Bradley, Amy Elsmore, Jenna Frizelle, Tabitha Jones, Abi Merriel, the Options Collaborative Group, Deborah Lawlor, Gordon Smith, Jane Norman, Jon Heron, Louise Kenny, Michael Lawton, Sheelagh McGuinness, Anna Davies, Dame Tina Lavender, Christy Burden, Jonathan Ives, Simon Grant, Sherif Abdel‐Fattah, Danya Bakhbakhi, Laura Bonnet, Andrew Demetri, Christopher Dewhurst, Mairead Black, Sam Finnikin, Amie Wilson, Alexandra Freeman, Pete Blair, Katherine Birchenall, Joanne Johnson, Gary Johnstone, Carol Kenyon, Amber Marshall, Michelle Maden, Andrew Sharp, Andy Sharp, Andrew Weeks

**Affiliations:** ^1^ Department of Women's and Children's Health, Institute of Life Course and Medical Sciences, Faculty of Health and Life Sciences University of Liverpool Liverpool UK; ^2^ Liverpool Women's Hospital Liverpool UK; ^3^ Health Data Science University of Liverpool Liverpool UK; ^4^ University of Bristol Bristol UK; ^5^ United Hospitals Bath Bath UK; ^6^ Cambridge University Hospitals NHS Foundation Trust UCLH Cambridge UK; ^7^ University of Keele Keele UK; ^8^ University of Aberdeen Aberdeen UK; ^9^ University Hospitals of Liverpool Group Liverpool UK

**Keywords:** birth, caesarean, choice, consent, core information set, Delphi

## Abstract

**Objective:**

To develop a caesarean birth core information set. Caesareans are the most common surgery performed in many countries. Women need information for informed decision‐making and consent. Core information sets (CISs) provide baseline information, agreed upon by parents and clinicians, for discussion before a procedure.

**Design:**

Two‐phase consensus study using modified Delphi.

**Setting:**

United Kingdom, 2024.

**Sample:**

People planning a pregnancy/currently pregnant/new parents and maternity professionals.

**Methods:**

Phase 1: Long‐list of information points identified from 273 systematic reviews, 50 patient leaflets, three pre‐existing qualitative studies and a stakeholder survey (*n* = 230); Operationalised into a Delphi questionnaire comprising 11 information points with 108 items. Phase 2: Think‐aloud interviews (*n* = 9) informed questionnaire restructure into information about planned caesarean birth, unplanned caesarean birth (within 72 h) and emergency caesarean birth (EMCB; ≤ 30 min), followed by two‐round Delphi survey and consensus meetings.

**Results:**

*N* = 360 participated in the Delphi survey Round 1. All items were carried forward, and three were added for Round 2 (*n* = 188/56.4% attrition rate). From Round 2, one item was removed, 73 included and 37 items no‐consensus. Free‐text responses identified an unmet need for a postnatal EMCB‐CIS. Over four meetings (*n* = 36) consensus was reached for an antenatal‐caesarean‐birth‐CIS (14 points), EMCB‐CIS (5 points) and a postnatal EMCB‐CIS (12 points).

**Conclusions:**

This study has established three caesarean birth CISs to support informed decision‐making discussions between women and clinicians: (1) an antenatal CIS for planned and unplanned caesareans when there is time for discussion in clinic; (2) a one‐page CIS for emergency caesarean birth (within 30 min) when there is little time for discussion; (3) and a postnatal CIS for use after an unplanned caesarean birth before hospital discharge.

**Trial Registration:**

COMET Initiative | Development of a core information set for caesarean birth (comet‐initiative.org)

## Abstract 2

**Linked article:** This article is commented on by Walker, pp. 2040–2041 in this issue. To view this article visit https://doi.org/10.1111/1471‐0528.70012.

## 1 Introduction

Global caesarean section use doubled between 2000 and 2015 [1]. Projections suggest this trend will continue [2]. In England, 42% of births in 2023–2024 were caesarean births [2]. Optimising caesarean use is a global health priority [1, 3, 4, 5]; the operation should be available to all in need, performed safely and informed by best evidence, when requested by women. In the United Kingdom (UK), emphasis is placed on choice [6, 7], decision‐making [8, 9] and consent [10]. The UK Supreme court Montgomery ruling highlighted that women should receive information about material risks and reasonable alternatives to clinicians' recommended treatment [10]. The UK's National Institute for Health and Care Excellence's (NICE) Caesarean Birth Guideline recommends women receive information and support to make informed decisions about mode of birth [11].

Research shows that information and involvement in decision‐making impact women's satisfaction with childbirth experience [12, 13, 14, 15]. A review of 52 studies, from 28 countries, found women can feel disempowered during decision‐making about caesarean birth [16]. However, little evidence exists to guide clinicians' about what information to give, when and how [17]. Women want information, but their needs vary [6, 9, 18]. Extensive information about caesarean birth is available from the UK's National Health Service (NHS) [19], Royal Colleges [20] and charities [21, 22, 23, 24]. There is also experiential knowledge shared between women in communities, and information on social networks, internet and mass media [25]. However, studies show women want consistent information about caesarean birth aligned with what clinicians say in clinical consultations [26]. A recent study suggests women are more likely to trust NHS‐branded information [27]. However, there is no single go‐to source of information about caesarean birth for clinicians and/or women.

Core information sets provide baseline information of importance to patients and clinicians, agreed upon by consensus, which should be discussed with every person before experiencing a procedure [28]. Core Information Sets offer a way to improve the consistency and quality of information patients receive and offer clinicians a means to balance over‐ and under‐disclosure of information. Unlike decision aids, which present alternative treatments [29]. Core information sets already exist for operations in different specialities [28, 29, 30]. This study is part of the Options programme, which includes developing core information sets for planning mode of birth [31, 32]. The objective of this study was to develop a caesarean birth core information set.

## 2 Methods

### 2.1 Study Design and Setting

We conducted a consensus study in the UK using a modified Delphi design adapted from core outcome sets for randomised controlled trials methods [28, 29], and an earlier core information set study [31]. The study was registered with the Core Outcome Measures in Effectiveness Trials (COMET) Initiative (2599) [33]. Adapted Core Outcome Set–STAndards for reporting (COS‐STAR) were used [34].

### 2.2 Phase 1—Development of Information Long‐List

We collated our long‐list of information points about caesarean birth from four sources.

#### 2.2.1 Systematic Review of Systematic Reviews

We searched electronic databases (Data S1) for information items and outcomes of interest. We sought systematic reviews in the English language, published within 5 years. These timeframes were deemed current and sufficient to achieve data saturation. No studies were excluded based on methodological quality or risk of bias, as this was irrelevant for extracting information points. Key information items were extracted from full‐texts and data summaries created.

#### 2.2.2 Review of Existing Patient Information

We used Google to identify trusted sources of patient information leaflets, articles and electronic information from Royal Colleges, NHS Trusts and charities (e.g., National Childbirth Trust). Information points were extracted.

#### 2.2.3 Review of Existing Qualitative Studies

We re‐analysed existing data from three qualitative studies about information provision and decision‐making to extract information points. All studies were conducted 2018–2022, and permitted data to be used in other studies.

- Antenatal Care Education project (IRAS262911‐REC19/SW/0073) information points from focus groups with women (*n* = 46) and staff (*n* = 21) about decision‐making during pregnancy [35].
- Shared decision‐making for labour and birth (IRAS277301‐REC20/SW/0035) information points from interviews with women (*n* = 11 postnatal), (*n* = 10 paired antenatal and postnatal) [27] and focus groups with staff (*n* = 24) about information for decision‐making during birth [18].
- Dataset 3: Vaginal Birth Core Information Set (University of Bristol REC10530). We sought information points from 17 interviews with women some of whom discussed caesarean birth [31].

#### 2.2.4 Online Survey of Key Stakeholders

We conducted a UK‐wide REDCaP survey [36], advertised on social media, exploring the information women want for caesarean birth. We sought participants who were women/people planning a pregnancy/pregnant/new parents and maternity professionals. Responses were analysed for information points.

Following duplicate removal and item grouping in consultation with patient advisors, the long‐list for a Delphi questionnaire was produced.

### 2.3 Phase 2—Modified Delphi Consensus Process

We developed the Delphi survey using REDCap software [36], piloted the survey using Think‐aloud interviews; completed a two‐round Delphi survey; held consensus meetings to agree content; consulted with patient advisors about format.

#### 2.3.1 Developing the Delphi Survey

REDCap [36] software was used to create an on‐line survey comprising key study information, consent, the questionnaire and a free‐text box for any additional comments. The target population was maternity care stakeholders (as in the survey above).

#### 2.3.2 Piloting and Refining of Delphi Questionnaire

To ensure usability and clarity of survey questions, we planned think‐aloud interviews with parents and professionals [37]. They are a form of cognitive interviewing that asks participants to discuss the content of the survey they are completing whilst completing it.

#### 2.3.3 Two‐Round Delphi Survey

**FIGURE 1:**
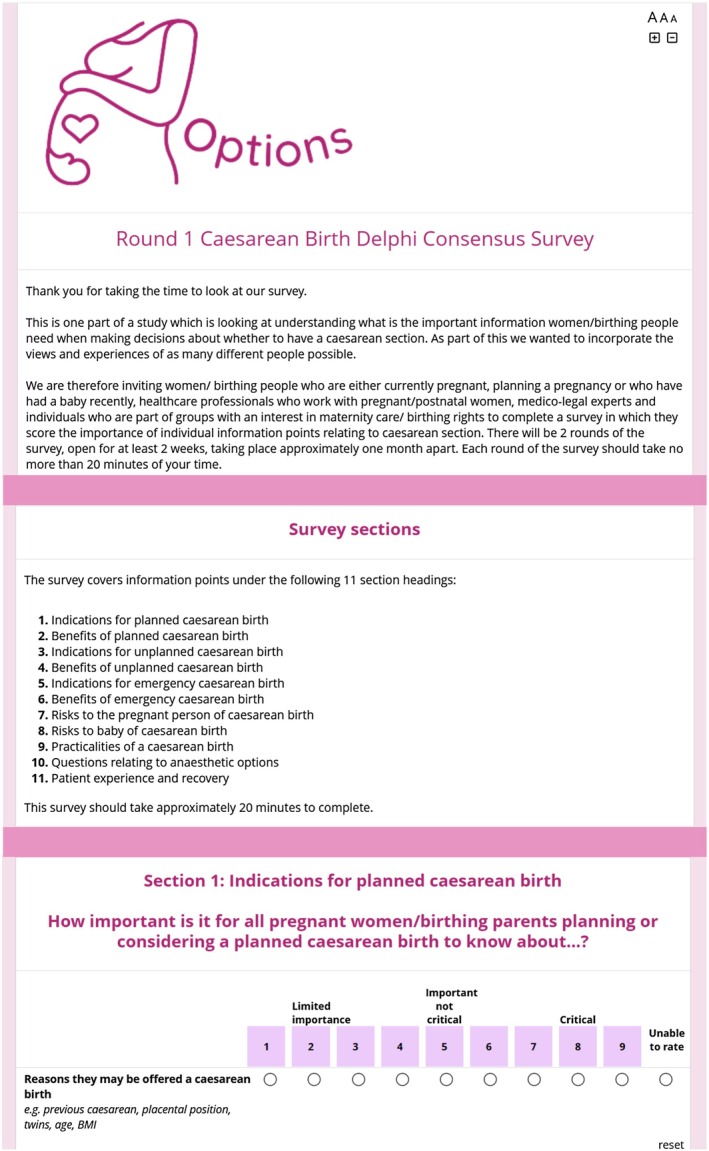
Delphi Questionnaire Round 1.

The first round of the survey (April 2024) was advertised on social media, via professional and parenting networks, five NHS hospitals and community children's centres. Participants rated the importance of information domains on a 9‐point Likert scale, ranging from 1 (limited importance) to 9 (critical importance) (Figure 1). To enhance accessibility, surveys were available in English, Arabic and Polish, two of the most common non‐English languages spoken in Liverpool where the study is based. We added a captcha to prevent Internet bots which can threaten sample validity and data integrity [38]. Items were discarded or carried forward from Round 1 to Round 2 according to predefined criteria (see consensus criteria). Round 2 questionnaires were sent to Round 1 participants, together with their scores, and the median scores and histograms for all participants. Round 2 of the survey closed in July 2024.

#### 2.3.4 Consensus Meetings

Participants in the survey were invited to online consensus meetings using Microsoft Teams. The retained items from Round 2 were presented, followed by discussion and voting on items for which consensus still had to be reached. Consensus to include was defined as ≥ 80% of attendees voting to ‘include’ the item; exclusion was defined as ≥ 80% of attendees voting to ‘exclude’ the item. Participants were shown graphs of patient and professional voting to inform discussions. Anonymised voting took place using Poll‐Everywhere software, which produced instant results. If no consensus was achieved after the first vote, discussion and re‐voting occurred, followed by discussion about grouping of items and order.

### 2.4 Sample Size

We undertook secondary data analysis from three qualitative studies, which collectively were sufficient to reach saturation for information items about caesarean birth. For the stakeholder survey, we sought a pragmatic sample with a minimum of 100 participants. There are no agreed methods to set the sample size for Delphi surveys or consensus meetings. Thus, we sought to obtain a sample with a broad range of experience, an approach supported by the COMET guidelines [39] and previous studies [29]. Using an opportunistic approach, we sought to engage at least 100 participants in the Delphi survey (patients and professionals) and a smaller group in the consensus meetings.

### 2.5 Consensus Criteria

A priori consensus criteria were set for the Delphi survey. *Consensus to include criteria*: if ≥ 80% of either stakeholder group scored an information item as critically important (7–9) and < 15% from either group scored it as of limited importance (1–3). *Consensus to exclude criteria*: ≥ 80% of one of the stakeholder groups voted the item as being of limited importance, and < 15% from either group believed it to be critically important. Items that did not meet criteria for inclusion or exclusion were carried forward from Round 1 to Round 2, and from Round 2 to consensus meetings.

### 2.6 Data Analysis

Delphi survey data were downloaded from REDCap into Microsoft Excel for analysis according to consensus criteria. Summary statistics and graphs were produced and presented at consensus meetings.

### 2.7 Ethics Statement

A favourable ethical opinion was granted 06/04/2023, by the Southwest Central Bristol Research Ethics Committee (REC23/SW/0022).

### 2.8 Patient Involvement

A GRIPP_2_ reporting checklist is provided (Data S2) [40]. Eighteen parents helped design the Options Programme. Options has a core patient involvement group and convened ad hoc groups to inform the Delphi format and information set(s). Ad hoc groups have been held with patients to inform the style, language and graphics used. In response to patients' requests, these were a mixture of on‐line and in‐person meetings, with two of the latter held in Children's Centres to encourage attendance from communities that do not typically participate in research. Patient involvement and Delphi participant responses from patients resulted in three core information sets being developed. This was a patient‐driven outcome.

## 3 Results

**FIGURE 2:**
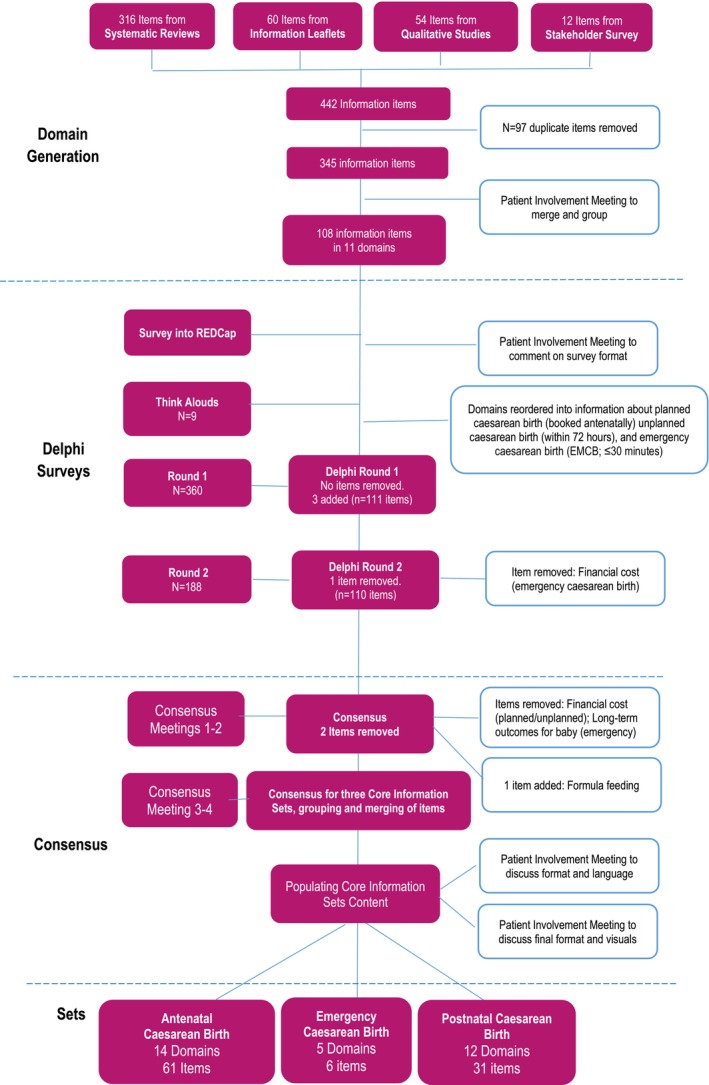
Caesarean birth core information set development flow chart.

A summary of the results is shown in Figure 2.

### 3.1 Phase 1: Information Domain Generation

From all data sources, we identified a total of 442 information items; 316 from 293 systematic reviews (Data S3), 60 from 50 patient information leaflets, 54 from qualitative studies (*n* = 59 individual interviews; 21 focus groups) and 12 from the stakeholder survey (*n* = 230 participants comprising parents [*n* = 58 planning pregnancy; *n* = 94 pregnant; *n* = 46 new parents; *n* = 7 partners], and professionals [*n* = 9 midwives; *n* = 7 obstetricians; *n* = 9 other]). Most respondents were white British (89%), with some Asian, Black African and Mixed‐race participants. Following duplicate removal 345 items remained. We grouped these into 11 broad categories/domains (i.e., indications, risks, benefits). During this process, similar items (i.e., anaesthetic options) were merged. A final 108 items, grouped into 11 domains were agreed in consultation with patient advisors before operationalising into the Phase 2 Delphi questionnaire (Data S4).

### 3.2 Phase 2: Delphi Consensus Process

#### 3.2.1 Survey

We developed the Delphi survey using REDCap software.

#### 3.2.2 Think Aloud Interviews

We conducted nine think‐aloud interviews with pregnant women, new parents and clinicians, resulting in a reordering of the questionnaire to reflect the degree of urgency with which decisions about caesarean births are made (*planned caesarean birth, unplanned caesarean birth* [*within 72 h*] *and emergency caesarean birth* [*EMCB; ≤ 30 min*]). The final Round 1 structure of the 11 domains/survey sections, is shown in Figure 1. There were no changes to the 108 information items within the 11 sections.

#### 3.2.3 Two‐Round Delphi Survey

The Delphi survey had 360 participants between 5 April 2024 and 24 July 2024. Two additional respondents were excluded as non‐UK residents. Table 1 lists demographic characteristics of participants in Rounds 1 and 2 (Data S5).

**TABLE 1:** Caesarean section core information set demographics.

Characteristics	Parents		Professionals and both a parent and professional	
	Round 1 (number & %)	Round 2 (number & %)	Round 1 (number & %)	Round 2 (number & %)
	*N* = 293^a^	*N* = 166^a^	Professionals *N* = 24	Professionals *N* = 11
			Parent and professional *N* = 21	Parent and professional *N* = 11
Roles (able to select more than one)				
As a parent				
Planning pregnancy	10/362 (2.8%)	6/207 (2.9%)	1/26 (3.8%)	1/15 (6.6%)
Currently pregnant	120/362 (33.1%)	67/207 (32.4%)	7/26 (26.9%)	4/15 (26.6%)
Given birth in past	229/362 (63.3%)	132/207 (63.8%)	18/26 (69.2%)	10/15 (66.6%)
Partner of someone who is currently/previously pregnant	3/362 (0.8%)	2/207 (1%)	0/26 (0%)	0/15 (0%)
As a professional				
Midwife			15/46 (32.6%)	7/21 (33.3%)
Midwife assistant			1/46 (2.2%)	1/21 (4.8%)
Obstetrics and gynaecology doctor			18/46 (39.1%)	9/21 (42.9%)
Anaesthetist			1/46 (2.2%)	0/21 (0%)
Member of charity organisation			1/46 (2.2%)	1/21 (4.8%)
Researcher			5/46 (10.9%)	1/21 (4.8%)
Other			5/46 (10.9%)	2/21 (9.5%)
Person who is currently pregnant—gestation in weeks	24 (10)	24 (10)	21 (11)	29 (5)
Number of children	1.36 (0.88)	1.37 (0.98)	1.35 (0.59)	1.45 (0.69)
Length of time since most recent birth	2.60 (1.70)	2.92 (1.85)	3.67 (3.39)	3.57 (2.88)
Person who has previously given birth—type of birth experienced (able to select more than one)				
Vaginal birth	80/298 (26.8%)	45/176 (25.6%)	9/19 (47.4%)	5/11 (45.5%)
Instrumental birth	52/298 (17.4%)	34/176 (19.3%)	2/19 (10.5%)	1/11 (9.1%)
Emergency caesarean birth	97/298 (32.6%)	58/176 (33%)	5/19 26.3%)	4/11 (36.4%)
Elective caesarean birth	69/298 (23.2%)	39/176 (22.2%)	3/19 (15.8%)	1/11 (9.1%)
Ethnicity				
Asian/Asian British	6/293 (2.0%)	5/166 (3.0%)	5/45 (11%)	2/22 (9.1%)
Black/African/Caribbean/Black British	4/293 (1.4%)	3/166 (1.8%)	0/45 (0%)	0/22 (0%)
Mixed/multiple ethnic groups	5/293 (1.7%)	1/166 (0.6%)	0/45 (0%)	0/22 (0%)
Other ethnic groups	2/293 (0.7%)	1/166 (0.6%)	3/45 (6.7%)	3/22 (14%)
Prefer not to say	1/293 (0.3%)	1/166 (0.6%)	0/45 (0%)	0/22 (0%)
White British	245/293 (84%)	135/166 (81%)	29/45 (64%)	15/22 (68%)
White Other	30/293 (10%)	20/166 (12%)	8/45 (18%)	2/22 (9.1%)
Age^b^	34 (5)	35 (5)	36 (9)	39 (11)
Age categories				
Under 21	1/293 (0.3%)	0/166 (0%)		
21–30	54/293 (18%)	21/166 (13%)	12/45 (27%)	4/22 (18%)
31–40	217/293 (74%)	132/166 (80%)	23/45 (51%)	12/22 (55%)
41–50	21/293 (7.2%)	13/166 (7.8%)	6/45 (13%)	2/22 (9.1%)
51–60	0/293 (0%)	0/166 (0%)	3/45 (6.7%)	3/22 (14%)
61–70	0/293 (0%)	0/166 (0%)	1/45 (2.2%)	1/22 (4.5%)
Highest level of education				
GCSEs or equivalent	11/291 (3.8%)	4/164 (2.4%)	0/21 (0%)	0/11 (0%)
A‐levels or equivalent	35/291 (12%)	15/164 (9.1%)	0/21 (0%)	0/11 (0%)
Bachelors degree or equivalent	122/291 (42%)	67/164 (41%)	11/21 (52%)	5/11 (45%)
Post‐graduate degree	120/291 (41%)	75/164 (46%)	10/21 (48%)	6/11 (55%)
Other	2/291 (0.7%)	2/164 (1.2%)	0/21 (0%)	0/11 (0%)
Prefer not to say	1/291 (0.3%)	1/164 (0.6%)	0/21 (0%)	0/11 (0%)
Employment				
Employed full time/on maternity leave	176/291 (60%)	99/164 (60%)	15/21 (71%)	7/11 (64%)
Employed part time/on maternity leave	84/291 (29%)	50/164 (30%)	5/21 (24%)	3/11 (27%)
Homemaker	15/291 (5.2%)	6/164 (3.7%)	0/21 (0%)	0/11 (0%)
Not currently employed	5/291 (1.7%)	2/164 (1.2%)	0/21 (0%)	0/11 (0%)
Other	6/291 (2.1%)	3/164 (1.8%)	0/21 (0%)	0/11 (0%)
Student	5/291 (1.7%)	4/164 (2.4%)	1/21 (4.8%)	1/11 (9.1%)
Area of residence				
East of England	20/293 (6.8%)	12/166 (7.2%)	3/21 (14%)	1/11 (9.1%)
London	15/293 (5.1%)	7/166 (4.2%)	1/21 (4.8%)	1/11 (9.1%)
Midlands	39/293 (13%)	21/166 (13%)	4/21 (19%)	2/11 (18$)
North East England and Yorkshire	32/293 (11%)	22/166 (13%)	1/21 (4.8%)	1/11 (9.1%)
North West England	68/293 (23%)	38/166 (23%)	6/21 (29%)	3/11 (27%)
Northern Ireland	12/293 (4.1%)	5/166 (3.0%)	0/21 (0%)	0/11 (0%)
Scotland	21/293 (7.2%)	11/166 (6.6%)	2/21 (9.5%)	0/11 (0%)
South East England	33/293 (11%)	19/166 (11%)	3/21 (14%)	3/11 (27%)
South West England	41/293 (14%)	24/166 (14%)	0/21 (0%)	0/11 (0%)
Wales	9/293 (3.1%)	5/166 (3.0%)	1/21 (4.8%)	0/11 (0%)
Other	1/293 (0.3%)	0/166 (0%)	0/21 (0%)	0/11 (0%)
Missing	2/293 (0.7%)	2/166 (1.2%)	0/21 (0%)	0/11 (0%)

*Note: n* = greater for some responses than number of participants because they could tick multiple answers.

^a^ *n*/*N* (%), Mean (SD).

^b^ Mean and SD for age was calculated using the midpoint from age categories.

##### 3.2.3.1 Round 1

293 participants identified as parents, 45 as professionals (21 of whom were professionals and parents), and 22 did not specify a stakeholder group. We were not able to calculate a response rate due to recruitment methods.

Most participants were female, amongst parent and health professional participants. There were a few male health professional participants (*n* = 5/45; 11%). Health professionals were also more ethnically diverse and had a wider age range. Most parents were aged 31–40. Many parents and professionals were educated to Bachelor's or post‐graduate degree level. Amongst parents, around a third of women had given birth within the last 6 months (*n* = 91/31%). Of the 229 women who had previously given birth, 27% had had a spontaneous vaginal birth (*n* = 80), 17% had an instrumental (*n* = 52), 33% (*n* = 97) had had an emergency caesarean and 23% (*n* = 69) an elective caesarean. Most professionals were Obstetricians and Gynaecologists (*n* = 18/46) or Midwives (*n* = 15/46); 21/46 were professionals and a parent (all of whom were female *n* = 21/21; most had one child *n* = 14/21). One important difference between the parents, and the professionals who were also parents, is that more professionals had had a vaginal birth (9/19), whilst more parents had had an emergency caesarean birth (97/298).

All items were retained for Round 2 (Data S6), with a further three added resulting in 111 items across 12 domains. The extra items encapsulated free‐text comments relating to short‐ and long‐term caesarean scar pain, and the third was about the importance of a postnatal debrief following emergency caesarean birth.

##### 3.2.3.2 Round 2

The response rate was 56% with comparable proportions of parents (*n* = 166) and professionals (*n* = 22) to Round 1. In Round 1, 87% were parents, and 88% were in Round 2. For professionals, 13% were in Round 1; 12% were in Round 2. The attrition rate between rounds was calculated minus exclusions from Round 1 (*n* = 22 did not specify parent or professional stakeholder group) or provide an email address for Round 2 (*n* = 5). There were few differences in the demographic profile of parents and professionals who responded to Rounds 1 and 2 when compared to those who only responded to Round 1. There were no professional participants from Wales or Scotland in Round 2.

In Round 2, professionals rated nine information items to be of greatest importance (scored 9). All nine items were also critically important to parents, who rated an additional six of the greatest importance. These items related to unplanned caesarean birth (indications for mother; and for baby; and other options for the birth of baby), emergency caesarean birth (other options for the birth of baby; benefits of the operation to baby) and planned/unplanned caesarean birth (serious conditions with short‐; and or long‐term risks to baby) (Data S7). Only one item was excluded (emergency caesarean birth: Financial cost to health), and 73 items were included. Of the 37 items that met the criteria to be discussed at the Consensus Meetings, 29 related to emergency caesarean birth, and 8 to planned/unplanned caesarean birth. The 73 items included because they scored ≥ 80% by either stakeholder group (critically important 7–9) and < 15% from either group (limited importance 1–3) are listed in Data S8.

### 3.3 Consensus Meetings

**FIGURE 3:**
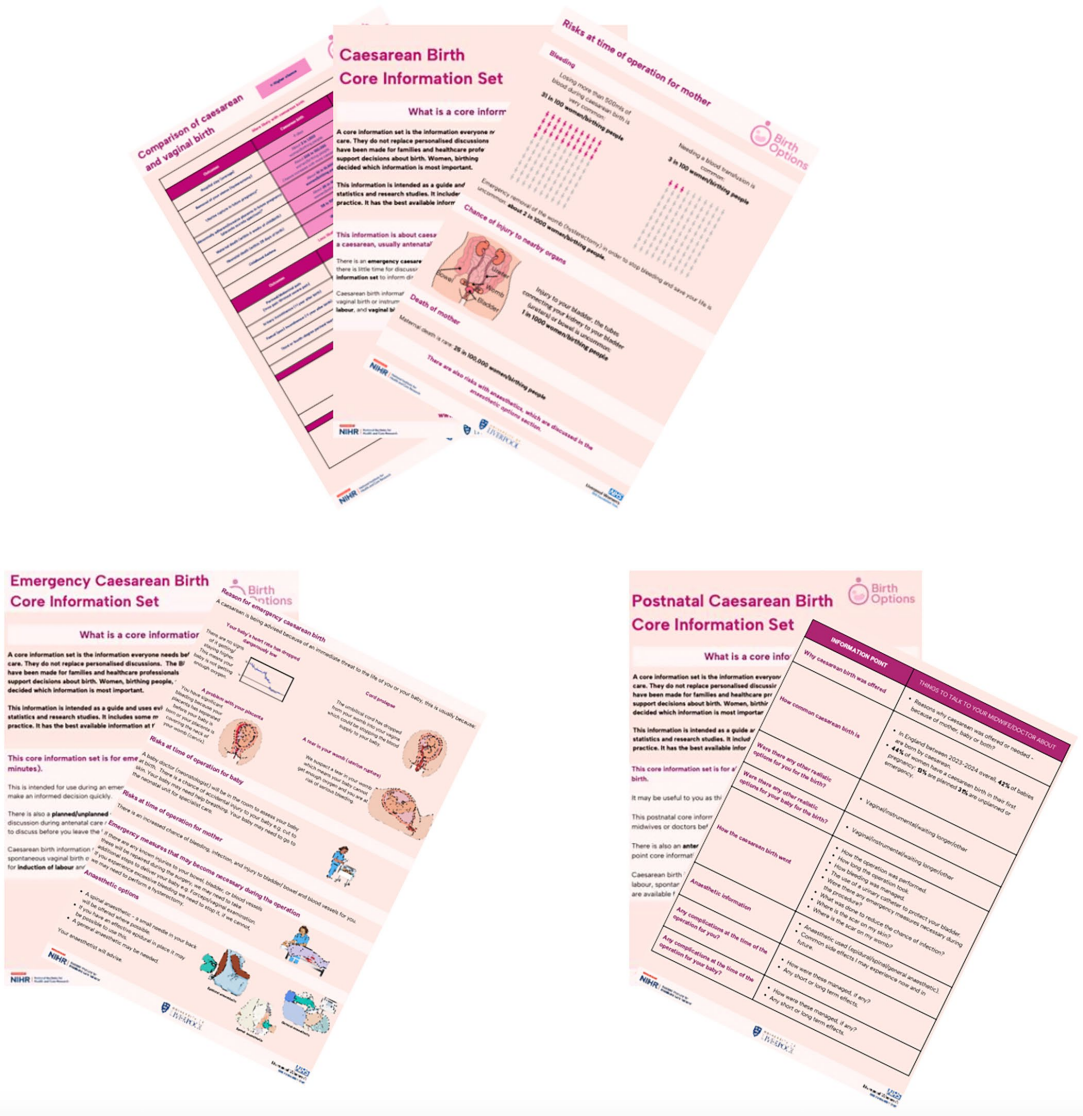
The three core information sets.

We held four consensus meetings with professionals and patients (*n* = 36) in summer 2024. All meetings were held on‐line. We were aiming to have one core information set with between 15 and 20 information points/domains. By the end of the third meeting, anonymous voting had reached a consensus on 14 domains to be discussed with all people planning to have, or considering, a caesarean birth, but controversy remained. Consensus was reached to exclude only two items relating to the financial cost of planned/unplanned caesareans and the long‐term risks to the baby of an emergency caesarean birth. As stated above, one of the additions to Round 2 of the survey for emergency caesarean birth was that someone will come and discuss with you why you needed the operation and any important longer‐term considerations. Many survey participants had an emergency caesarean birth and so had many of the consensus meeting participants. At the fourth meeting an agreement was reached for an antenatal‐caesarean‐birth‐CIS (14 points), EMCB‐CIS (5 points) and a postnatal EMCB‐CIS (12 points)—Figure 3.

The content of the final Caesarean Birth Core Information Set for use as part of antenatal care and when there is time for discussion comprises 14 information points, within which there is information on 61 items relating to planned/unplanned and emergency caesarean birth. The major changes from the consensus meetings were the inclusion of information items that participants wanted to know about emergency caesarean births in this set, (so they had had information in advance should the need arise), and the ordering of information points to reflect women's decision‐making journeys and life‐course. As shown in Table 2, participants wanted a comparison of the risks and benefits of caesarean birth and vaginal birth upfront (point 5), and information about future pregnancies last (point 14). One new information point was added (How formula feeding can be supported) (Data S9).

**TABLE 2:** Final domains and information items in caesarean birth core information sets.

Caesarean birth core information set (for use antenatally or when have time to discuss options)			
1	*Why a caesarean birth may be offered?* Maternal reasons they may be offered a planned caesarean birth Maternal reasons they may be offered an unplanned caesarean birth Reasons a planned caesarean may be offered because of the baby Reasons an unplanned caesarean may be offered because of the baby	8	*Anaesthetic options* Regional and general anaesthetic Benefits and risks of regional anaesthetic Benefits and risks of general anaesthetic Common side effects of regional anaesthetic
2	*What are the other options for the birth of your baby?* Other options for the birth of the baby, for example spontaneous/induced birth (planned) If there are other options for the birth of the baby depending on the circumstances, for example induced birth, continuing with labour (unplanned & emergency)	9	*Comparison of planned caesarean and vaginal birth* The risk of a planned caesarean birth compared to planned vaginal birth The benefits of a planned caesarean birth compared to a planned vaginal birth
3	*What if things change?* Changing their mind after deciding to have a caesarean birth (planned & unplanned) When they can decide to have a caesarean birth (planned & unplanned) When they can decide to have a caesarean birth (unplanned) What may happen if they have decided to have a caesarean and go into spontaneous labour (planned & unplanned)	10	*Preparing for your planned caesarean birth* How consent is taken How to prepare for the operation, for example when to stop eating and drinking What to do on the day
4	*What are the benefits of caesarean birth?* Benefits of the operation to themselves (planned, unplanned & emergency) Benefits of the operation to baby (planned, unplanned & emergency) Contraceptive or sterilisation options that can be performed during the operation	11	*What to expect during a caesarean birth* What happens during the operation, including presence of partners How the operation is performed, including possible variations, reasons & effects How long the operation usually takes How bleeding is routinely managed The routine use of a urinary catheter to protect your bladder What can be done to reduce infection, for example routine use of antibiotics prior to birth, vaginal cleaning prior to the operation starting That skin to skin and early breastfeeding can usually be facilitated Where the scar on their skin will be and its appearance
5	*Risks at time of operation for mother* Common complications (less than 1 in 10 to 1 in 100) Uncommon complications (less than 1 in 100 to 1 in 1000) Rare complications (less than 1 in 1000 to 1 in 10 000) Very rare complications (less than 1 in 10 000)	12	*Recovering after a caesarean birth* How long until bowel function returns Vaginal bleeding after a caesarean birth Pain management whilst in hospital and at home Caesarean scar pain in the short and long term
6	*Risk at time of operation for baby* The risks to baby during the operation (e.g., a scratch or cut to baby) Emergency measures that may become necessary during the procedure The potential for baby to need help breathing after being born The potential for baby to need admission to the neonatal intensive care unit and for how long	13	*What to expect following a caesarean birth* Pain Feeding your baby (breastfeeding and formula); skin to skin in theatre. What happens after the operation (e.g., food and drink, moving to the ward, walking, wound dressing) When the catheter is removed and how long until normal bladder function usually returns Length of time in hospital. Practical aspects of recovery (e.g., exercise and heavy lifting, sexual activity, driving, medications) The use of blood thinning medication to reduce the risk of blood clots
7	*Risks following the operation* Significant maternal complications during the caesarean birth requiring further surgery Serious maternal illness during or after birth that may result in long‐term hospital admission or consequences The risk of future pelvic floor related problems, for example pelvic organ prolapse, inability to control bladder or bowels Impact on maternal mental health Serious conditions with short or long term risks to baby Long term conditions that may be associated with caesarean for baby	14	*Future pregnancies following a caesarean birth* The effects of birth by caesarean on future pregnancies, including low lying placenta (placenta previa); invasive placenta (placenta accreta), stillbirth, womb rupture and future birth options.

Emergency caesarean birth core information set			
1	*Reason for emergency caesarean birth* Maternal reasons an emergency caesarean birth is being offered Reasons an emergency caesarean birth may be offered because of the baby	4	*Emergency measures that may be necessary during the operation* Emergency measures that may become necessary during the procedure, for example use of forceps to deliver baby, other ways to control bleeding including further surgery
2	*Risks at the time of operation for baby* The potential for baby to need admission to the neonatal intensive care unit for extra care and how long this admission may be needed	5	*Anaesthetic options* Anaesthetic options, for example spinal (an injection into the back to numb from the chest down) or general anaesthetic (being put to sleep for the operation)
3	*Risks at time of operation for mother* Significant complications during the caesarean birth requiring further surgery, for example hysterectomy, bowel damage, urine system damage		

Postnatal caesarean birth core information set (for use after an unplanned or emergency caesearean)			
1	Why caesarean birth was offered	10	*Recovering after a caesarean birth* How long until bowel function returns Vaginal bleeding after a caesarean birth Pain management whilst in hospital and at home Caesarean scar pain in the short and long term
2	How common caesarean birth is		
3	Were there any other realistic options for you for the birth		
4	Were there any other realistic options for your baby for the birth		
5	*How the caesarean birth went* How the operation was performed How long the operation took How bleeding was managed Use of a urinary catheter to protect your bladder Emergency measures that were necessary during the procedure What was done to reduce infection Where the scar on skin is		
		11	*What to expect following a caesarean birth* Pain Feeding your baby (breastfeeding and formula); skin to skin in theatre What happens after the operation (e.g., food and drink, moving to the ward, walking, wound dressing) When the catheter is removed and how long until normal bladder function usually returns Length of time in hospital. Practical aspects of recovery (e.g., exercise and heavy lifting, sexual activity, driving, medications) The use of blood thinning medication to reduce the risk of blood clots
6	*Anaesthetic information* Anaesthetic used Common side effects of anaesthetic they may experience		
7	Any complications during the operation for the mother	12	*Future pregnancies following a caesarean birth* The effects of birth by caesarean on future pregnancies, including low lying placenta (placenta previa); invasive placenta (placenta accreta), stillbirth and womb rupture. Vaginal birth after caesarean (VBAC)
8	Any complications during the operation for the baby		
9	*Risks following the operation* Significant maternal complications during the caesarean birth requiring further surgery Serious maternal illness during or after birth that may result in long‐term hospital admission or consequences The risk of future pelvic floor related problems, for example pelvic organ prolapse, inability to control bladder or bowels Impact on maternal mental health Serious conditions with short or long term risks to baby Long term conditions that may be associated with caesarean birth for baby		

To reach a consensus regarding core information that every woman should know for emergency caesarean birth (birth within ≤ 30 min) participants were asked to rank the 10 information items they most need to know to make an informed decision if their and/or their baby's life is in jeopardy. All participants acknowledged that in this scenario there is no time for discussion. The result is a short 5‐information point core Information set (with 6 items) (Data S10). Consensus participants felt this was sufficient, especially if pregnant women/birthing parents had received the above antenatal core information set and then all received a post‐natal core information guided conversation. The post‐natal caesarean birth core information guided conversation set comprises 12 points (Data S11).

### 3.4 Populating the Core Information Sets

The three caesarean birth information sets were populated using a pre‐defined hierarchy of sources including NICE, RCOG Green Top Guidelines and systematic reviews (with a priority for Cochrane). If the information was not available from these individual randomised or non‐randomised studies were used. All sources are referenced at the end of the sets as in the companion vaginal birth [41] and induction [32] core information sets. Once populated patient involvement meetings were held to agree the final format. Patient involvement resulted in changes to length, layout and risk communication methods. In the antenatal set one item was felt to be duplicated and three in the postnatal set. The sets (Data S12–S14) have been designed for multiple media use following patient advisors and existing research suggesting women want paper and electronic options [26].

## 4 Discussion

This study aimed to develop a caesarean birth core information set to inform antenatal discussions about planning birth mode. Phase 1 identified a range of information; Phase 2 reached consensus about critical‐to‐know information. This study has developed three core information sets detailing the minimum information clinicians should discuss about caesarean birth in the antenatal period, the emergency setting and when an immediate postnatal debrief is taking place following an unplanned/emergency birth. Collectively, they address what information, when and why. We propose as a minimum all pregnant people should be provided with the antenatal core information set for caesarean birth. These three sets were driven by the needs of research participants and patient involvement contributors, who highlighted the need for these three companion caesarean birth core information sets. A third of parents who responded to the Delphi survey had had a baby in the last 6 months, with most experiencing an emergency caesarean birth, which reflects current trends in use in England. Nationally, amongst women aged 31–40, the proportion of caesarean (planned/unplanned/emergency) and spontaneous vaginal births is now similar [2]. In nulliparous women, the emergency caesarean rate is 31%.

### 4.1 Strengths and Limitations

We used a robust consensus methodology to develop three sets reconciling what women want to know when, with what professionals think women should know, to facilitate informed decision‐making about and consent for planned, unplanned and emergency caesarean birth. A strength is the mean age of participants was similar to the UK birthing population [2]. The attrition between Delphi rounds is a limitation, but commensurate with similar surveys [42, 43, 44]. Participant demographics were comparable between rounds. Fewer professionals than parents, and no medicolegal professionals, participated in the stakeholder survey, two‐round Delphi survey and consensus groups. A limitation is that we did not collect information on the number of participants for which English was not their first language. Patient involvement was embedded into all stages of the research process and in the production of the final sets. This is a key strength, helping to balance educational and ethnicity equity considerations. During groups culturally sensitive translation, health literacy, language alongside use of illustrations and multiple formats (paper and digital) were discussed and acted upon. The emergency caesarean birth set is designed with explanatory images and words that require little or no translation. The final sets were acceptable to patients and professionals as end users. They have been designed in the context for which they are intended, where caesarean procedures are governed by national guidance. Additional development work would be required in other contexts. Either the methodology could be replicated to develop further sets appropriate for other settings (including low resource or fee‐paying facilities), or these sets could be tested in other contexts and modified accordingly.

Pregnant women need information about caesarean birth to know what to expect to help them make decisions. Existing research shows women use sources of varying quality, for information about caesarean birth [25, 45, 46]. In the UK much research about caesarean birth information has focused on interventions to inform decision‐making that promote vaginal birth [4, 47]. However over the last decade the legal requirement for informed decision‐making has been made clearer [6, 7, 8, 9, 10]. Irrespective of which mode of birth the information relates to the challenge is women are not homogeneous in their information requirements [9], nor are professionals consistent in the information they provide [3, 18] and pregnancy and birth are dynamic and demand fluidity in decision‐making [6]. The development of three companion core information sets was unanticipated, but obvious when involving women and professionals in equitable information development. They offer a way to improve the consistency and quality of information patients receive and balance over‐ and under‐discussion of information across antenatal, intrapartum and postnatal care tailored to what women want to know, and when. The views of participants in this study resonate with existing research reporting that women understand in an obstetric emergency there is little time for discussion [48, 49].

Items excluded included the financial cost to health service of emergency and planned caesareans. These operations can be lifesaving procedures with financial considerations critical to most settings. Consensus regarding the removal of this item may be unique to the UK with its free‐at‐point‐of‐access NHS. Beyond the range of items covered in the sets, another vital dimension of information quality is the evidence underpinning materials. NICE recommends further research into the short‐ and long‐term outcomes of caesarean birth [11]. In other contexts, there have been calls for randomised trials of planned vaginal and planned caesarean birth in low‐risk populations [50, 51, 52] and national surveillance studies now exist [53]. Maternal request for caesarean section is an established global phenomena [54]. Further research is required to address implementation considerations for information sets, short‐ and long‐term health outcomes for women, and current drivers of caesarean use.

### 4.2 Conclusion

This study has established three caesarean birth core information sets: (1) for planned and unplanned caesareans when there is time for discussion; (2) for emergency caesareans (within 30 min); (3) post‐emergency caesareans pre‐hospital discharge. They are intended to provide a minimum set of information to support discussions between parents and professionals planning mode of birth. They are not intended to replace individualised conversations but to offer a standardised starting point. Further evaluation will be required to assess their use and whether they can contribute to more equitable care for all women across ethnic and socio‐economic groups and improve their actual birth experience.

## Author Contributions

A.M. produced the protocol and supporting documentation for this study with support from A.D. A.M., M.M., J.F., T.J., B.G. and A.E. undertook the systematic review, A.E. reviewed the patient information leaflets, F.B. extracted themes from the stakeholder survey and A.M. re‐analysed the qualitative data. C.K. and B.G. performed the think‐aloud interviews. G.J., E.B., A.H., C.K., V.B., A.M., B.G. and L.B. undertook the Delphi and consensus meetings and analysis. M.A., A.M., E.B. and C.K. populated the core information sets. C.C., S.H., C.B., J.F., E.O.‐R., W.P.S. and M.B. were the site PIs for the study and recruited participants. All Options Steering Group members attended meetings to plan and disseminate the study. All authors read and approved the final manuscript. A.M. is the guarantor for the manuscript.

## Disclosure

The words women/mother is used as a collective term in this paper, but we acknowledge those that identify by other terms.

## Conflicts of Interest

The authors declare no conflicts of interest.

## Supporting information


Data S1.

Data S2.

Data S3.

Data S4.

Data S5.

Data S6.

Data S7.

Data S8.

Data S9.

Data S10.

Data S11.

Data S12.

Data S13.

Data S14.

## Data Availability

The data that support the findings of this study are available from the corresponding author upon reasonable request.
