# Supplementary material for: Informing Decision‐Making About Caesarean Birth: A Delphi Study to Develop a Core Information Set
Source: BJOG. 2025 Jul 8;132(13):2024–39. doi: 10.1111/1471-0528.18269 (PMC12592771; doi:10.1111/1471-0528.18269)
Supplement: Supplementary file 1 — Data S1. [file BJO-132-2024-s008.docx]

**Caesarean systematic reviews search**

| **Database** | **Date searched** | **Results** |
| --- | --- | --- |
| MEDLINE (Ovid) | 11/4/23 | 1571 |
| EMBASE (Ovid) | 11/4/23 | 2941 |
| CINAHL (EBSCOHOST) | 11/4/23 | 1071 |
| PsycINFO (EBSCOHOST) | 11/4/23 | 78 |
| The Cochrane Library (CSDR) | 11/4/23 | 95 |
| NIHR Journals Library [HTA/Evidence Synthesis] <https://www.journalslibrary.nihr.ac.uk/#/> | 11/4/23 | 0 |
| International Network of Agencies for Health Technology Assessment (INAHTA)  <https://database.inahta.org> | 11/4/23 | 0 |
| Epistemonikos <https://www.epistemonikos.org/> | 11/4/23 | 1327 |
| Health Evidence  <https://www.healthevidence.org/> | 11/4/23 | 29 |
| DoPHER (Database of promoting health effectiveness reviews)  <https://eppi.ioe.ac.uk/cms/Default.aspx?tabid=62> | 11/4/23 | 0 |
| **Total across all databases** | | 7112 |
| **Duplicates removed** | | 3686 |
| **Total in Endnote** | | 3426 |

**Note: Searches limited to systematic reviews, English language, 2018-2023**

**Ovid MEDLINE(R) ALL <1946 to April 10, 2023>**

1 exp Cesarean Section/

2 cesarea*.mp.

3 caesarea*.mp.

4 caesaria*.mp.

5 "c section*".mp.

6 postcesarea*.mp.

7 postcaesarea*.mp.

8 abdominal deliver*.mp.

9 LSCS.mp.

10 or/1-9

11 systematic review.pt.

12 meta-analysis.pt.

13 systematic review/

14 meta analysis/

15 network meta-analysis/

16 (0266-4623 or 1469-493X or 1366-5278 or 1530-440X or 2046-4053).is.

17 (systematic review? or evidence report* or technology assessment?).jw.

18 (meta-analys* or meta analys* or metaanalys* or meta synth* or meta-synth* or metasynth*).mp.

19 ((systematic or meta) adj2 (analys* or review)).ti,kw.

20 ((systematic* or methodologic*) adj5 (review* or overview*)).mp.

21 (integrative research review* or research integration or (evidence adj3 review*)).mp.

22 ("review of reviews" or umbrella review).ti,ab,kw.

23 (overview adj2 review*).mp.

24 cochrane review*.mp.

25 (quantitativ* adj5 synthes*).mp.

26 (qualitativ* adj5 synthes*).mp.

27 (realist adj (review or synthes*)).mp.

28 (rapid review or rapid evidence assessment).mp.

29 (mixed method? adj1 (review or synthes*)).mp.

30 framework synthes*.mp.

31 (metaethnography or meta-ethnography).mp.

32 (meta-aggregation or meta-interpretation).mp.

33 (metatheory or meta-theory or metastudy or meta-study).mp.

34 (thematic synthes* or thematic analys*).mp. and review.pt,ti.

35 "interpretive synthes*".mp.

36 (narrative adj (review or synthes*)).mp.

37 ((scoping or mapping) adj1 review).mp.

38 or/11-37

39 10 and 38

40 limit 39 to (english language and yr="2018 -Current")

**Embase <1974 to 2023 April 07>**

1 exp cesarean section/

2 cesarea*.mp.

3 caesarea*.mp.

4 caesaria*.mp.

5 "c section*".mp.

6 postcesarea*.mp.

7 postcaesarea*.mp.

8 abdominal deliver*.mp.

9 LSCS.mp.

10 or/1-9

11 "systematic review"/

12 meta analysis/

13 network meta-analysis/

14 (0266-4623 or 1469-493X or 1366-5278 or 1530-440X or 2046-4053).is.

15 (systematic review? or evidence report* or technology assessment?).jw.

16 (meta-analys* or meta analys* or metaanalys* or meta synth* or meta-synth* or metasynth*).mp.

17 ((systematic or meta) adj2 (analys* or review)).ti,kw.

18 ((systematic* or methodologic*) adj5 (review* or overview*)).mp.

19 (integrative research review* or research integration or (evidence adj3 review*)).mp.

20 ("review of reviews" or umbrella review).mp.

21 (overview adj2 review*).mp.

22 cochrane review*.mp.

23 (quantitativ* adj5 synthes*).mp.

24 (qualitativ* adj5 synthes*).mp.

25 (realist adj (review or synthes*)).mp.

26 (rapid review or rapid evidence assessment).mp.

27 (mixed method? adj1 (review or synthes*)).mp.

28 framework synthes*.mp.

29 (metaethnography or meta-ethnography).mp.

30 (meta-aggregation or meta-interpretation).mp.

31 (metatheory or meta-theory or metastudy or meta-study).mp.

32 (thematic synthes* or thematic analys*).mp. and review.pt,ti.

33 "interpretive synthes*".mp.

34 (narrative adj (review or synthes*)).mp.

35 ((scoping or mapping) adj1 review).mp.

36 or/11-35

37 10 and 36

38 limit 37 to (english language and yr="2018 - 2023")

**CINAHL via EBSCOHOST**

S1 (MH "Cesarean Section+")

S2 cesarea*

S3 caesarea* or caesaria*

S4 "c section*"

S5 postcesarea*

S6 postcaesarea*

S7 “abdominal deliver*” or LSCS

S8 S1 OR S2 OR S3 OR S4 OR S5 OR S6 OR S7

S9 PT systematic review

S10 PT meta analysis

S11 PT meta synthesis

S12 IS (0266-4623 or 1469-493X or 1366-5278 or 1530-440X or 2046-4053)

S13 (systematic review? or evidence report* or technology assessment?)

S14 (meta-analys* or meta analys* or metaanalys* or meta synth* or meta-synth* or metasynth*)

S15 TI ( ((systematic or meta) N2 (analys* or review)) ) OR SU ( ((systematic or meta) N2 (analys* or review)) )

S16 ((systematic* or methodologic*) N5 (review* or overview*))

S17 (integrative research review* or research integration or (evidence N3 review*))

S18 ("review of reviews" or umbrella review)

S19 (overview N2 review*)

S20 "cochrane review*"

S21 (quantitativ* N5 synthes*)

S22 (qualitativ* N5 synthes*)

S23 (realist N0 (review or synthes*))

S24 (rapid review or rapid evidence assessment)

S25 (mixed method? N1 (review or synthes*))

S26 "framework synthes*"

S27 (metaethnography or meta-ethnography)

S28 (meta-aggregation or meta-interpretation)

S29 (metatheory or meta-theory or metastudy or meta-study)

S30 ( (thematic synthes* or thematic analys*) ) AND PT review

S31 ( (thematic synthes* or thematic analys*) ) AND TI review

S32 "interpretive synthes*"

S33 (narrative N0 (review or synthes*))

S34 ((scoping or mapping) N1 review)

S35 (MH "Systematic Review")

S36 (MH "Cochrane Library")

S37 (MH "Meta Analysis")

S38 (MH "Meta Synthesis")

S39 S9 OR S10 OR S11 OR S12 OR S13 OR S14 OR S15 OR S16 OR S17 OR S18 OR S19 OR S20 OR S21 OR S22 OR S23 OR S24 OR S25 OR S26 OR S27 OR S28 OR S29 OR S30 OR S31 OR S32 OR S33 OR S34 OR S35 OR S36 OR S37 OR S38

S40 S8 AND S39

S41 S8 AND S39, Limiters - Publication Year: 2018-2023, Narrow by Language: - english

**PsycINFO via EBSCOHOST**

S1 DE "Caesarean Birth"

S2 cesarea*

S3 caesarea* or caesaria*

S4 "c section*"

S5 postcesarea*

S6 postcaesarea*

S7 “abdominal deliver*” or LSCS

S8 S1 OR S2 OR S3 OR S4 OR S5 OR S6 OR S7

S9 (systematic review? or evidence report* or technology assessment?)

S10 (meta-analys* or meta analys* or metaanalys* or meta synth* or meta-synth* or metasynth*)

S11 TI ( ((systematic or meta) N2 (analys* or review)) ) OR SU ( ((systematic or meta) N2 (analys* or review)) )

S12 ((systematic* or methodologic*) N5 (review* or overview*))

S13 (integrative research review* or research integration or (evidence N3 review*))

S14 ("review of reviews" or umbrella review)

S15 (overview N2 review*)

S16 "cochrane review*"

S17 (quantitativ* N5 synthes*)

S18 (qualitativ* N5 synthes*)

S19 (realist N0 (review or synthes*))

S20 (rapid review or rapid evidence assessment)

S21 (mixed method? N1 (review or synthes*))

S22 "framework synthes*"

S23 (metaethnography or meta-ethnography)

S24 (meta-aggregation or meta-interpretation)

S25 (metatheory or meta-theory or metastudy or meta-study)

S26 ( ( (thematic synthes* or thematic analys*) ) ) AND TI review

S27 "interpretive synthes*"

S28 (narrative N0 (review or synthes*))

S29 ((scoping or mapping) N1 review)

S30 DE "Systematic Review"

S31 DE "Meta Analysis"

S32 S9 OR S10 OR S11 OR S12 OR S13 OR S14 OR S15 OR S16 OR S17 OR S18 OR S19 OR S20 OR S21 OR S22 OR S23 OR S24 OR S25 OR S26 OR S27 OR S28 OR S29 OR S30 OR S31

S33 S8 AND S32

S34 S8 AND S32 Limiters - Published: 20180101-20231231, Narrow by Language: - english

**Cochrane CDSR**

#1 MeSH descriptor: [Cesarean Section] explode all trees

#2 (cesarea*):ti,ab,kw

#3 (caesarea*):ti,ab,kw

#4 (caesaria*):ti,ab,kw

#5 ("c section*"):ti,ab,kw

#6 (postcesarea*):ti,ab,kw

#7 (postcaesarea*):ti,ab,kw

#8 (abdominal NEXT deliver*):ti,ab,kw

#9 (LSCS):ti,ab,kw

#10 {OR #1-#9}

**NIHR Journals Library**

[HTA/Evidence synthesis records] <https://www.journalslibrary.nihr.ac.uk/#/>

Cesarea* OR caesarea* OR caesaria OR c-section

Limit to 2018-2023

**International Network of Agencies for Health Technology Assessment (INAHTA)**

<https://database.inahta.org>

Cesarea* OR caesarea* OR caesaria OR c-section

Limit to 2018-2023

**Epistemonikos**

<https://www.epistemonikos.org/>

(title:(cesarea* OR caesarea* OR caesaria* OR "c section*" OR postcesarea* OR postcaesarea* OR "abdominal deliver*" OR LSCS) OR abstract:(cesarea* OR caesarea* OR caesaria* OR "c section*" OR postcesarea* OR postcaesarea* OR "abdominal deliver*" OR LSCS))

Date = Published from 2018 to 2023

Publication Type = Systematic Review

**Health Evidence**

<https://www.healthevidence.org/>

(cesarea* OR caesarea* OR caesaria* OR "c section*" OR postcesarea* OR postcaesarea* OR "abdominal deliver*" OR LSCS) AND Limit:

Date = Published from 2018 to 2023

Review Type = Meta-analysis, Narrative review, Systematic review of reviews

**DoPHER (Database of promoting health effectiveness reviews)**

<https://eppi.ioe.ac.uk/cms/Default.aspx?tabid=62>

Browse through list of titles
