# Supplementary material for: Informing Decision‐Making About Caesarean Birth: A Delphi Study to Develop a Core Information Set
Source: BJOG. 2025 Jul 8;132(13):2024–39. doi: 10.1111/1471-0528.18269 (PMC12592771; doi:10.1111/1471-0528.18269)
Supplement: Supplementary file 2 — Data S2. [file BJO-132-2024-s004.docx]

**GRIPP_2_-SF Report checklist**

| **Section and topic** | **Item** | **Reported on page No** |
| --- | --- | --- |
| 1: Aim | To collaboratively involve patients and public advisors in the Core Options PPI group as partners engaged in all stages of the development of the caesarean birth core information set. To involve current patients and new parents in ad hoc PPI meetings to inform the content of the information set. | 5 |
| 2: Methods | Ad hoc groups have been held with patients to inform the style, language, and graphics used. In response to patient’s requests, these were a mixture of on-line and in-person meetings, with two of the latter held in Children's Centres to encourage comment from communities that do not typically participate in research but attend regular groups there. | 5 |
| 3: Study results | Patient involvement resulted in changes to the length, layout and risk communication methods to include percentages and icon-arrays. The sets have been designed for multiple media use with women wanting paper and electronic copies. | 9 |
| 4: Discussion and conclusions | Patient involvement was embedded into all stages of the research process and in the production of the final sets. This is a key strength that helped to balance educational and ethnicity equity considerations. During groups culturally sensitive translation, health literacy, language alongside use of illustrations and multiple formats (paper and digital) were discussed and acted upon. The emergency caesarean birth set is designed with explanatory images and words that require little or no translation when there is no time for discussion. | 9-10 |
| 5: Reflections/critical perspective | The design of the final sets has benefited from the involvement of patients with recent experience of maternity care. Further evaluation will be required to assess use by women, and whether they can contribute to more equitable care for all women across ethnic and socio-economic groups. | 11 |

**Reference:** Staniszewska S, Brett J, Simera I, Seers K, Mockford C, Goodlad S et al. GRIPP2 reporting checklists: tools to improve reporting of patient and public involvement in research *BMJ*2017; 358 :j3453 doi:10.1136/bmj.j3453
