# Supplementary material for: Informing Decision‐Making About Caesarean Birth: A Delphi Study to Develop a Core Information Set
Source: BJOG. 2025 Jul 8;132(13):2024–39. doi: 10.1111/1471-0528.18269 (PMC12592771; doi:10.1111/1471-0528.18269)
Supplement: Supplementary file 3 — Data S3. [file BJO-132-2024-s006.docx]

Studies from databases/registers **(n = 3427)**

**Identification**

Studies included in review **(n = 273)**

Studies excluded **(n = 3095)**

Studies not retrieved **(n = 2)**

Studies assessed for eligibility **(n = 292)**

Studies sought for retrieval **(n = 294)**

Studies screened **(n = 3400)**

Studies excluded **(n = 19)**

Non-English (n=2)

Duplicates (n=6)

Wrong study design (n=11)

References removed **(n = 27)**

Duplicates identified manually (n = 7)

Duplicates identified by Covidence (n = 20)

Marked as ineligible by automation tools (n = 0)

**Screening**

**Included**
