# Supplementary material for: Informing Decision‐Making About Caesarean Birth: A Delphi Study to Develop a Core Information Set
Source: BJOG. 2025 Jul 8;132(13):2024–39. doi: 10.1111/1471-0528.18269 (PMC12592771; doi:10.1111/1471-0528.18269)
Supplement: Supplementary file 4 — Data S4. [file BJO-132-2024-s002.docx]

**Long-list**

Information point long list (n = 345) and initial categorisation prior to think aloud interview refinement.

| Indications for caesarean (40) | Maternal indication for caesarean? | Maternal request |
| --- | --- | --- |
|  | Fetal indications for caesarean? | Fear of childbirth/tokophobia |
|  | Other options? | Life-threatening maternal complications |
|  |  | Life-threatening fetal complications |
|  |  | Clinical influence |
|  | Specifics for emergency situation? | Simultaneous sterilisation |
|  |  | Maternal age |
|  |  | Prenatal UI |
|  | Separate EMCS and ELCS | Obesity |
|  |  | Preterm |
|  |  | Non-obstetric medical condition |
|  |  | Socioeconomic factors |
|  |  | Fetal injury |
|  |  | Pelvic floor trauma |
|  |  | Timing of birth |
|  |  | Previous experience |
|  |  | Previous infertility |
|  |  | Control |
|  |  | Avoidance of prolonged labour |
|  |  | Lack of support |
|  |  | Fear of pain |
|  |  | Fear of faeces |
|  |  | Estimated fetal weight |
|  |  | Short birth interval |
|  |  | Previous caesarean |
|  |  | Indications for emergency caesarean |
|  |  | Classification of emergency caesarean |
|  |  | Maternal rights |
|  |  | Alternatives |
|  |  | Do nothing |
|  |  | Common indications |
|  |  | What can happen if you do nothing |
|  |  | What if labour spontaneously |
|  |  | Patient/family preference |
|  |  | Caesarean rates |
|  |  | Consent process including emergency |
|  |  | Comparison to IOL |
|  |  | Ability to change mind |
|  |  | Ability to ask for unplanned caesarean |
|  |  | Difficulty decision making in pain for emergency |

| Maternal risks of caesarean (51) | Common complications | Morbidity increase with successive caesareans |
| --- | --- | --- |
|  | Uncommon complications | Surgical site infection |
|  | Rare complications | Visceral injury |
|  | Severe and significant risks | Hysterectomy |
|  | Bleeding complications | Death |
|  | Wound complications | Wound dehiscence |
|  | Infection complications | Wound haematoma |
|  | Effects on future pregnancy | Anaesthetic complications |
|  | Effects on future health | VTE |
|  | Effects on pelvic floor | ICU admission |
|  | Psychological effects | Blood transfusion |
|  | Risks vs vaginal delivery/IOL | Future PAS |
|  |  | Future stillbirth |
|  |  | PPH |
|  |  | Urinary incontinence |
|  |  | Faecal incontinence |
|  |  | Endometritis |
|  |  | Sepsis |
|  |  | Failure of simultaneous sterilisation |
|  |  | Difficult fetal extraction |
|  |  | Difficult operative steps |
|  |  | Abnormal placentation |
|  |  | Side effects to medications |
|  |  | UTI |
|  |  | Skin reactions/blistering |
|  |  | Urinary retention |
|  |  | Pruritis |
|  |  | Longer time to breastfeeding initiation |
|  |  | Incisional hernia |
|  |  | Dental caries in childhood |
|  |  | Hypothermia |
|  |  | PET |
|  |  | Perinatal death |
|  |  | Stillbirth |
|  |  | Neonatal hypoglycaemia after steroids |
|  |  | Anaesthetic medication side effects |
|  |  | Acute pain |
|  |  | Chronic pain |
|  |  | Anaemia |
|  |  | Post dural puncture headache |
|  |  | Midline incision |
|  |  | Childhood eczema risk |
|  |  | Uterine rupture in future pregnancy |
|  |  | Haemodynamic side effects |
|  |  | Mental health postnatally |
|  |  | SUI |
|  |  | Morbidity with extreme preterm |
|  |  | Anxiety |
|  |  | Long term reduction in HRQoL |
|  |  | Fascial dehiscence |
|  |  | Rectus diastasis |

| Benefits of caesarean (12) | Maternal benefits | Reduced pelvic floor dysfunction |
| --- | --- | --- |
|  | Neonatal benefits | Protective against intrapartum SB |
|  | Benefits vs vaginal delivery/IOL | Avoids macrosomia adverse effects |
|  |  | Allows timing |
|  |  | Control |
|  |  | Lifesaving in emergencies |
|  |  | Reduced risk of morbidity/mortality in presence of complications |
|  |  | Avoids risks of labour |
|  |  | Autonomy |
|  |  | Choice |
|  |  | Support |
|  |  | Protective against sexual dysfunction |

| Surgical technique (23) | Expected surgical technique | Suture material choice |
| --- | --- | --- |
|  | Variation, reasoning and effects | Subcutaneous vs interrupted closure |
|  | Emergency measures | Incision type and location |
|  | Potential need for forceps delivery during delivery | Use of drains |
|  |  | Peritoneal closure |
|  |  | Placental removal (manual vs spontaneous) |
|  |  | Vaginal preparation/cleansing |
|  |  | Uterine incision |
|  |  | Hysterotomy closure (single, double, locking, non-locking) |
|  |  | Sterilisation method (TL vs salpingectomy) |
|  |  | Intrauterine cleansing |
|  |  | Hysterotomy expansion |
|  |  | T incision |
|  |  | Skin preparation types |
|  |  | Use of fetal pillow |
|  |  | Use of Abdominal Aortic Balloon Occlusion in PAS |
|  |  | Wound irrigation |
|  |  | Glove change |
|  |  | Classical caesarean |
|  |  | Clips vs suture |
|  |  | Use of O-ring retractors |
|  |  | Operative field considerations |
|  |  | Use of forceps for delivery |

| Process (14) | Management of bleeding inc blood transfusion | Use of uterotonics |
| --- | --- | --- |
|  | Measures to reduce infection risk | Use of TXA |
|  | Comfort measures | Antibiotic (dosing, choice) |
|  | How to facilitate skin to skin and feeding | Timing of antibiotics (pre-incision, post cord-clamping) |
|  |  | Pain relief |
|  |  | Antiemetics |
|  |  | Estimated Blood Loss |
|  |  | Nausea and vomiting |
|  |  | Hypothermia |
|  |  | Shivering |
|  |  | Warming techniques |
|  |  | Operative time |
|  |  | Ability to facilitate early skin to skin |
|  |  | Ability to facilitate early feeding |

| Anaesthetic concerns (20) | Options? | Hypotension |
| --- | --- | --- |
|  | Benefits and risks of spinal anaesthesia | Bradycardia |
|  | Benefits and risks of general anaesthesia | Pruritis |
|  |  | Conversion to GA |
|  |  | GA as a choice |
|  |  | Failed spinal block |
|  |  | Requirement for additional analgesia/anaesthetic duration |
|  |  | High spinal block as a complication |
|  |  | Need for vasopressor |
|  |  | Tachycardia |
|  |  | Reactive hypertension |
|  |  | Various LA techniques (ilioinguinal, ilioinguinal, hypogastric, transversalis fascia, erector spinae, transversus abdominis, quadratus lumborum, wound infiltration) |
|  |  | Wound catheter |
|  |  | Respiratory depression |
|  |  | Shivering |
|  |  | Nausea and vomiting |
|  |  | Options to reduce N&V |
|  |  | Magnesium sulfate |
|  |  | Adjunctive analgesia with GA |
|  |  | Post dural puncture headache |

| Decision making and process (49) | Schedule of caesarean week | Involvement of woman and partner |
| --- | --- | --- |
|  | How to prepare for operation | Shared decision making |
|  | Consent process | Provider preference and influence |
|  | What happens during the operation | Process of actually arraning/booking a caesarean |
|  | What happens after the operation | Tokophobia |
|  |  | Facilitation of e.g. complementary medicine choices |
|  |  | Choice of timing |
|  |  | Blood transfusion |
|  |  | Skin to skin |
|  |  | Delayed cord clamping |
|  |  | Warming |
|  |  | Shivering |
|  |  | Pain |
|  |  | Nausea and vomiting |
|  |  | Surgical technique |
|  |  | Anaesthetic risks |
|  |  | Antibiotics |
|  |  | Uterotonics |
|  |  | Dressing type (negative pressure wound therapy) |
|  |  | Operative time |
|  |  | Pre-op process |
|  |  | Pre-op blood tests |
|  |  | Pre-op medication (e.g. antacid) |
|  |  | Fasting instructions |
|  |  | How consent is obtained |
|  |  | MRSA swabs and implication |
|  |  | Shaving instructions |
|  |  | Removal of nail varnish/jewellery/piercing |
|  |  | Change into gown |
|  |  | Team introductions and WHO safety checks |
|  |  | Who can be present and for what stages of process |
|  |  | Necessity of cannula/IV access |
|  |  | Patient positioning (on back, tilted left) |
|  |  | Catheter insertion and duration |
|  |  | Screen/operating theatre set up |
|  |  | Additional complexities with multiple pregnancy |
|  |  | Rules around recovery room/postnatal ward visiting |
|  |  | When can shower |
|  |  | When can mobilise |
|  |  | When can eat and drink |
|  |  | How to manage wound |
|  |  | TWOC process |
|  |  | Physical support post-operatively |
|  |  | Expected bleeding |
|  |  | Emotional trauma for partner in emergency |
|  |  | Post-caesarean advice e.g. lifting/driving/contraception |
|  |  | Average length of stay |
|  |  | Length of stay if emergency |
|  |  | Length of stay if baby on NICU/needing tests or treatment |

| Baby related (46) | Immediate risk | APGAR scores |
| --- | --- | --- |
|  | Short term risks | Cord pH |
|  | Long term risks | NNU admission |
|  | Breastfeeding suport | NNU length of stay |
|  |  | Mother-infant bonding |
|  |  | Transient tachypnoea of newborn risk |
|  |  | Respiratory morbidity |
|  |  | Respiratory distress |
|  |  | Sepsis |
|  |  | Sepsis workup |
|  |  | Antibiotics |
|  |  | Intubation |
|  |  | CPAP |
|  |  | Respiratory support |
|  |  | Better vaccine response |
|  |  | Asphyxia |
|  |  | Laceration |
|  |  | Adverse events |
|  |  | Neonatal death |
|  |  | Stillbirth |
|  |  | Hypoglycaemia |
|  |  | Hypothermia |
|  |  | Early breastfeeding |
|  |  | Failure of breastfeeding |
|  |  | Breastfeeding at discharge |
|  |  | Asthma |
|  |  | T1DM |
|  |  | IBD |
|  |  | ALL |
|  |  | Obesity |
|  |  | Neurodiversity |
|  |  | Allergies |
|  |  | Immune and metabolic disorder |
|  |  | Psychiatric illness |
|  |  | Changes to gut microbiome |
|  |  | Coeliac disease |
|  |  | Lower birthweight |
|  |  | Dental caries |
|  |  | Enamel defects |
|  |  | Wheeze |
|  |  | Atopic |
|  |  | Dermatitis |
|  |  | Eczema |
|  |  | ADHD |
|  |  | Early neonatal weight loss |
|  |  | Birth trauma/delivery injury |

| Recovery (38) | Bleeding complications | PPH |
| --- | --- | --- |
|  | Wound complications | Hb drop/anaemia |
|  | Infection complications | Wound infection |
|  | Pain management | Wound dehiscence |
|  | VTE risk and prevention | Wound haematoma |
|  |  | Wound seroma |
|  |  | Organ or space infection |
|  |  | Wound appearance |
|  |  | Keloid scarring |
|  |  | Endometritis |
|  |  | Fever |
|  |  | Sepsis |
|  |  | UTI |
|  |  | Respiratory infection |
|  |  | Pyelonephritis |
|  |  | Nausea and vomiting |
|  |  | Return to bowel function |
|  |  | Ileus |
|  |  | Benefits of chewing gum |
|  |  | Pain |
|  |  | Analgesia |
|  |  | Opioids or other modality |
|  |  | VTE |
|  |  | Pharmaceutical VTE prophylaxis |
|  |  | Mechanical VTE prophylaxis |
|  |  | Urinary retention |
|  |  | Time to catheter removal |
|  |  | Return to theatre/reoperation |
|  |  | Sedation |
|  |  | Bruising |
|  |  | Blood pressure changes |
|  |  | Hallucination |
|  |  | Post dural puncture headache |
|  |  | Abdominal binder |
|  |  | Transfusion related adverse effects |
|  |  | Readmission to hospital |
|  |  | Length of stay |
|  |  | Breastfeeding support |

| Maternal morbidity (15) | Significant risks | Mortality |
| --- | --- | --- |
|  |  | ITU admission |
|  |  | Uterine vessel injury |
|  |  | Hysterotomy extension |
|  |  | Bladder damage |
|  |  | Bowel damage |
|  |  | Ureter damage |
|  |  | Tube or ovarian damage |
|  |  | Hysterectomy |
|  |  | MRSA |
|  |  | Necrotising fasciitis |
|  |  | Zeptic thrombophlebitis |
|  |  | Shock |
|  |  | Coma |
|  |  | Organ failure |

| Long term effects (37) | Effects on future pregnancy | Infertility |
| --- | --- | --- |
|  | (Uro)gynaecological effects | Asherman's syndrome |
|  | Psychological effects inc emergency | Adhesions |
|  |  | Tubal infertility |
|  |  | Niche related infertility |
|  |  | Residual myometrial thickness |
|  |  | Niche or isthmocele prevalence |
|  |  | QoL (physical/mental/pain/social) |
|  |  | Dysmennorhoea |
|  |  | Menorrhagia |
|  |  | Loss of control |
|  |  | Positive/negative birth experience |
|  |  | Natural birth |
|  |  | Sleep disturbance |
|  |  | When to resume regular life post-op |
|  |  | Dyspareunia |
|  |  | Pelvic floor function |
|  |  | Urogenital hiatial area |
|  |  | Levator ani defects |
|  |  | Bladder neck mobility |
|  |  | Puborectalis defects |
|  |  | Postpartum UI |
|  |  | Faecal incontinence |
|  |  | Avoidance of OASI |
|  |  | Pelvic organ prolapse |
|  |  | Perineal pain |
|  |  | Chronic pain |
|  |  | Stress urinary incontinence |
|  |  | Access to support structures |
|  |  | Scar appearance |
|  |  | Comfort |
|  |  | Low self esteem |
|  |  | Distress (acute vs chronic) |
|  |  | PTSD |
|  |  | Postpartum depression |
|  |  | Anxiety |
|  |  | Contraception post caesarean |
