# Supplementary material for: Informing Decision‐Making About Caesarean Birth: A Delphi Study to Develop a Core Information Set
Source: BJOG. 2025 Jul 8;132(13):2024–39. doi: 10.1111/1471-0528.18269 (PMC12592771; doi:10.1111/1471-0528.18269)
Supplement: Supplementary file 5 — Data S5. [file BJO-132-2024-s014.docx]

**Full demographic table for Delphi Round 1 and 2 participants**

Caesarean section core information sets

|  | **Parents** | | **Professionals and both a parent and professional** | |
| --- | --- | --- | --- | --- |
| **Characteristics** | Round 1 (number & %) N=293*^1^* | Round 2 (number & %) N=166*^1^* | Round 1 (number & %) Round 2 (number & %)  *Professionals N = 24 Professionals N = 11*  *Parent & Professional Parent & Professional N=*  *N = 21 11* | |
| **Roles (able to select more than one)** |  |  |  |  |
| **As a parent:** |  |  |  |  |
| Planning pregnancy | 10/362 (2.8%), | 6/207 (2.9%), | 1/26 (3.8%) | 1/15 (6.6%) |
| Currently pregnant | 120/362 (33.1%), | 67/207 (32.4%), | 7/26 (26.9%) | 4/15 (26.6%) |
| Given birth in past | 229/362 (63.3%), | 132/207 (63.8%), | 18/26 (69.2%) | 10/15 (66.6%) |
| Partner of someone who is currently/previously pregnant | 3/362 (0.8%), | 2/207 (1%), | 0/26 (0%) | 0/15 (0%) |
| **As a professional:** |  |  |  |  |
| Midwife |  |  | 15/46 (32.6%) | 7/21 (33.3%) |
| Midwife assistant |  |  | 1/46 (2.2%) | 1/21(4.8%) |
| Obstetrics and gynaecology Doctor |  |  | 18/46 (39.1%) | 9/21 (42.9%) |
| Anaesthetist |  |  | 1/46 (2.2%) | 0/21 (0%) |
| Member of charity organisation |  |  | 1/46 (2.2%) | 1/21 (4.8%) |
| Researcher |  |  | 5/46 (10.9%) | 1/21 (4.8%) |
| Other |  |  | 5/46 (10.9%) | 2/21 (9.5%) |
| **Person who is currently pregnant - Gestation in weeks** | 24 (10) | 24 (10) | 21 (11) | 29 (5) |
| **Number of children** | 1.36 (0.88) | 1.37 (0.98) | 1.35 (0.59) | 1.45 (0.69) |
| **Length of time since most recent birth** | 2.60 (1.70) | 2.92 (1.85) | 3.67 (3.39) | 3.57 (2.88) |
| **Person who has previously given birth -**  **Type of birth experienced**  **(able to select more than one)** |  |  |  |  |
| Vaginal birth | 80/298 (26.8%), | 45/176 (25.6%), | 9/19 (47.4%) | 5/11 (45.5%) |
| Instrumental birth | 52/298 (17.4%), | 34/176 (19.3%), | 2/19 (10.5%) | 1/11 (9.1%) |
| Emergency caesarean birth | 97/298 (32.6%), | 58/176 (33%), | 5/19 26.3%) | 4/11 (36.4%) |
| Elective caesarean birth | 69/298 (23.2%), | 39/176 (22.2%), | 3/19 (15.8%) | 1/11 (9.1%) |
| **Gender** |  |  |  |  |
| Female | 292/293 (99.7%), | 166/166 (100%), | 40/45 (89%) | 19/22 (86%) |
| Male | 0/293 (0%), | 0/166 (0%), | 5/45 (11%) | 3/22 (14%) |
| Prefer not to say | 1/293 (0.3%), | 0/166 (0%), | 0/45 (0%) | 0/22 (0%) |
| **Ethnicity** |  |  |  |  |
| Asian/Asian British | 6/293 (2.0%), | 5/166 (3.0%), | 5/45 (11%) | 2/22 (9.1%) |
| Black/African/Caribbean/Black British | 4/293 (1.4%), | 3/166 (1.8%), | 0/45 (0%) | 0/22 (0%) |
| Mixed/multiple ethnic groups | 5/293 (1.7%), | 1/166 (0.6%), | 0/45 (0%) | 0/22 (0%) |
| Other ethnic groups | 2/293 (0.7%), | 1/166 (0.6%), | 3/45 (6.7%) | 3/22 (14%) |
| Prefer not to say | 1/293 (0.3%), | 1/166 (0.6%), | 0/45 (0%) | 0/22 (0%) |
| White British | 245/293 (84%), | 135/166 (81%), | 29/45 (64%) | 15/22 (68%) |
| White Other | 30/293 (10%), | 20/166 (12%), | 8/45 (18%) | 2/22 (9.1%) |
| **Age*^2^*** | 34 (5) | 35 (5) | 36 (9) | 39 (11) |
| **Age categories** |  |  |  |  |
| Under 21 | 1/293 (0.3%), | 0/166 (0%), |  |  |
| 21-30 | 54/293 (18%), | 21/166 (13%), | 12/45 (27%) | 4/22 (18%) |
| 31-40 | 217/293 (74%), | 132/166 (80%), | 23/45 (51%) | 12/22 (55%) |
| 41-50 | 21/293 (7.2%), | 13/166 (7.8%), | 6/45 (13%) | 2/22 (9.1%) |
| 51-60 | 0/293 (0%) | 0/166 (0%) | 3/45 (6.7%) | 3/22 (14%) |
| 61-70 | 0/293 (0%) | 0/166 (0%) | 1/45 (2.2%) | 1/22 (4.5%) |
| **Highest level of education** |  |  |  |  |
| GCSEs or equivalent | 11/291 (3.8%), | 4/164 (2.4%), | 0/21 (0%) | 0/11 (0%) |
| A-levels or equivalent | 35/291 (12%), | 15/164 (9.1%), | 0/21 (0%) | 0/11 (0%) |
| Bachelors degree or equivalent | 122/291 (42%), | 67/164 (41%), | 11/21 (52%) | 5/11 (45%) |
| Post-graduate degree | 120/291 (41%), | 75/164 (46%), | 10/21 (48%) | 6/11 (55%) |
| Other | 2/291 (0.7%), | 2/164 (1.2%), | 0/21 (0%) | 0/11 (0%) |
| Prefer not to say | 1/291 (0.3%), | 1/164 (0.6%), | 0/21 (0%) | 0/11 (0%) |
| **Employment** |  |  |  |  |
| Employed full time/On maternity leave | 176/291 (60%), | 99/164 (60%), | 15/21 (71%) | 7/11 (64%) |
| Employed part time/On maternity leave | 84/291 (29%), | 50/164 (30%), | 5/21 (24%) | 3/11 (27%) |
| Homemaker | 15/291 (5.2%), | 6/164 (3.7%), | 0/21 (0%) | 0/11 (0%) |
| Not currently employed | 5/291 (1.7%), | 2/164 (1.2%), | 0/21 (0%) | 0/11 (0%) |
| Other | 6/291 (2.1%), | 3/164 (1.8%), | 0/21 (0%) | 0/11 (0%) |
| Student | 5/291 (1.7%), | 4/164 (2.4%), | 1/21 (4.8%) | 1/11 (9.1%) |
| **Area of residence** |  |  |  |  |
| East of England | 20/293 (6.8%), | 12/166 (7.2%), | 3/21 (14%) | 1/11 (9.1%) |
| London | 15/293 (5.1%), | 7/166 (4.2%), | 1/21 (4.8%) | 1/11 (9.1%) |
| Midlands | 39/293 (13%), | 21/166 (13%), | 4/21 (19%) | 2/11 (18$) |
| North East England and Yorkshire | 32/293 (11%), | 22/166 (13%), | 1/21 (4.8%) | 1/11 (9.1%) |
| North West England | 68/293 (23%), | 38/166 (23%), | 6/21 (29%) | 3/11 (27%) |
| Northern Ireland | 12/293 (4.1%), | 5/166 (3.0%), | 0/21 (0%) | 0/11 (0%) |
| Scotland | 21/293 (7.2%), | 11/166 (6.6%), | 2/21 (9.5%) | 0/11 (0%) |
| South East England | 33/293 (11%), | 19/166 (11%), | 3/21 (14%) | 3/11 (27%) |
| South West England | 41/293 (14%), | 24/166 (14%), | 0/21 (0%) | 0/11 (0%) |
| Wales | 9/293 (3.1%), | 5/166 (3.0%), | 1/21 (4.8%) | 0/11 (0%) |
| Other | 1/293 (0.3%), | 0/166 (0%), | 0/21 (0%) | 0/11 (0%) |
| Missing | 2/293 (0.7%), | 2/166 (1.2%), | 0/21 (0%) | 0/11 (0%) |
| **Area of work (professionals)** |  |  |  |  |
| East of England |  |  | 11/45 (24%) | 4/22 (18%) |
| London |  |  | 6/45 (13%) | 4/22 (18%) |
| Midlands |  |  | 4/45 (8.9%) | 2/22 (9.1%) |
| North East England and Yorkshire |  |  | 1/45 (2.2%) | 1/22 (4.5%) |
| North West England |  |  | 12/45 (27%) | 6/22 (27%) |
| South East England |  |  | 3/45 (6.7%) | 3/22 (14%) |
| South West England |  |  | 3/45 (6.7%) | 2/22 (9.1%) |
| Wales |  |  | 2/45 (4.4%) | 0/22 (0%) |
| Scotland |  |  | 2/45 (4.4%) | 0/22 (0%) |
| Other |  |  | 1/45 (2.2%) | 0/22 (0%) |
| *^1^*n/N (%),; Mean (SD) | | |  |  |
| *^2^*Mean and sd for age was calculated using the midpoint from age categories | | |  |  |
