# Supplementary material for: Informing Decision‐Making About Caesarean Birth: A Delphi Study to Develop a Core Information Set
Source: BJOG. 2025 Jul 8;132(13):2024–39. doi: 10.1111/1471-0528.18269 (PMC12592771; doi:10.1111/1471-0528.18269)
Supplement: Supplementary file 6 — Data S6. [file BJO-132-2024-s013.docx]

**Delphi Round 1 Results**

| **Question** | **% crit important** | **% not important** | **carry into round 2?** | **consensus?** |
| --- | --- | --- | --- | --- |
| reasons they may be offered a caesarean birth e.g previous caesarean | 81.60% | 0.90% |  |  |
| reasons a caesarean birth may be offered because of baby | 84% | 0.60% |  |  |
| other options for birth of baby e.g. spontaneous or induced birth | 86.80% | 0.90% |  |  |
| changing their mind after deciding to have a caesarean | 82.60% | 1.50% |  |  |
| when they can decide to have a caesarean birth | 78.90% | 1.50% |  |  |
| what may happen if they decide to have a caesarean and go into spontaneous labour | 88.40% | 0.90% |  |  |
| benefits of the operation to themselves e.g. feeling in control, minimising chance of AVB | 78.70% | 2.10% |  |  |
| benefits of operation to baby e.g. reduced risk of shoulder dystocia | 81.40% | 1.80% |  |  |
| benefits of caesarean birth compared to a vaginal birth | 73.70% | 7.20% |  |  |
| reasons they may be offered an unplanned caesarean birth e.g. concerns bout how the labour is progressing, developing infection. | 95.80% | 0.60% |  |  |
| reasons a caesarean birth may be offered because of the baby e.g. concerns how baby is coping with labour | 95.50% | 0.30% |  |  |
| if there are other options for the birth of the baby depending on the circumstance | 93.40% | 0.30% |  |  |
| changing their mind after deciding to have a caesarean birth | 84.90% | 2.40% |  |  |
| when they can decide to have a caesarean birth | 80.60% | 2.70% |  |  |
| what will happen if they decide to have a caesarean and then go into spontaneous labour | 87.30% | 2.10% |  |  |
| benefits of the operation to themselves e.g. feeling in control, minimising chance of AVB | 82.30% | 3.10% |  |  |
| benefits of the operation to the baby e.g. less exposure to developing stress or infection | 84.90% | 1.50% |  |  |
| section 5 q 1: maternal reasons they would be advised to have an emergency caesarean birth e.g. serious concerns regarding their health | 94.10% | 0.90% |  |  |
| s5 q2: reasons they would be advised to have an emergency caesarean because of concerns regardign baby | 96.30% | 0.90% |  |  |
| s5 q3: other options for the birth fo the baby e.g. induced birth, continuing with labour | 86.40% | 3.30% |  |  |
| s6 q1: benefits of the operation to themselves e.g. control of bleeding or infection, reduced risk of vaginal or perineal tear | 81.30% | 2.70% |  |  |
| s6 q2: benefits of the operation to baby e.g. reduced risk of stillbirth in labour | 90.70% | 1.20% |  |  |
| s7 q1: planned/unplanned: very common complications (risk more than 1 in 10 | 91.50% | 1.20% |  |  |
| s7 q1ii: emergency: very common complications | 74.20% | 5.30% |  |  |
| s7 q2i: planned/unplanned common complications | 92.80% | 0% |  |  |
| s7 q2ii: emergency: common complications | 74.80% | 4.70% |  |  |
| s7 q3i: planned/unplanned: uncommon complocations | 85.50% | 1.20% |  |  |
| s7 q3ii: emergency: uncommon complications | 68.20% | 8.40% |  |  |
| s7 q4i: planned/unplanned rare complications | 74.90% | 5.90% |  |  |
| s7 q4ii: emergency: rare complications | 59.70% | 15.10% |  |  |
| s7 q5i: planned/ unplanned: significant complications during the caesarean birth requiring further surgery | 89.90% | 0.60% |  |  |
| s7 q5ii: emergency: significant complciations during caesarean birth requiring further surgery | 73.30% | 6.60% |  |  |
| s7 q6i: planned/unplanned: serious illness during or after birth that may result in long term hospital admission | 88% | 0.60% |  |  |
| s7 q6ii: emergency: serious illness during or after birth that may result in long term hospital admission | 70.30% | 7.30% |  |  |
| s7 q7i: planned/unplanned: the effects of birth by caesarean on future pregnancies e.g. low lying placenta | 89.60% | 0.60% |  |  |
| s7 q7ii: emergency: the effects of birth by caesarean on future pregnancies e.g. low lying placenta | 62.60% | 10.70% |  |  |
| s7 q8i: planned/unplanned: the risk of future pelvic floor related problems | 83.70% | 1.50% |  |  |
| s7 q8ii: emergency: the risk of future pelvic floor related problems | 56.60% | 12.90% |  |  |
| s7 q9i: planned/unplanned: the psychological effects of birth (especially unplanned mode of delivery | 79.30% | 1.80% |  |  |
| s7 q9ii: emergency: the psychological effects of birth | 62.90% | 11.40% |  |  |
| s7 q10i: planned/unplanned: liklihood of pain after a caesarean | 83% | 2.50% |  |  |
| s7 q10ii: emergency: the liklihood of pain after a caesarean birth | 58.70% | 11.40% |  |  |
| s7 q11i: planned/unplanned: the risk of a caesarean birth compared to vaginal birth | 90.20% | 1.80% |  |  |
| s7 q11ii: emergency: the risk of a caesarean birth compared to a vaginal birth | 64% | 12.60% |  |  |
| s8 q1i: planned/unplanned: the risks to baby during the operation | 89.90% | 0.90% |  |  |
| s8 q1ii: emergency: the risks to baby during the operation | 68.30% | 8.20% |  |  |
| s8 q2i: planned/unplanned: the potential for baby to need help breathing after being born | 88.90% | 0.60% |  |  |
| s8 q2ii: emergency: the potential for baby to need help breathing after being born | 76.20% | 5% |  |  |
| s8 q3i: planned/unplanned: the potential for baby to need admission to the neonatal intensive care unit | 86.40% | 1.80% |  |  |
| s8 q3ii: emergency: the potential for baby to need admission to the neonatal intensive care unit | 72.80% | 6.30% |  |  |
| s8 q4: planned/unplanned: serious conditions with short or long term risks to baby | 86.10% | 1.50% |  |  |
| s8 q4ii: emergency: serious conditions with short or long term risks to baby | 71.90% | 10.80% |  |  |
| s8 q5i: planned/unplanned: longterm conditions that may be associated with caesarean birth | 76.20% | 4.80% |  |  |
| s8 q5ii: emergency: long term conditions that may be associated with caesarean birth to the baby | 38.30% | 17.40% |  |  |
| s9 q1: planned/unplanned: how the operation is performed | 73.30% | 3.80% |  |  |
| s9 qi: emergency: how the operation is performed | 45.70% | 20% |  |  |
| s9 q2i: planned/unplanned how long the operation usually takes | 63.20% | 6.60% |  |  |
| s9 q2ii: emergency: how long the operation usually takes | 44.40% | 20.30% |  |  |
| s9 q3i: planned/unplanned: how bleeding is routinely managed | 64.10% | 4.80% |  |  |
| s9 q3ii: emergency: how bleeding is routinely managed | 45.80% | 17.50% |  |  |
| s9 q4i: planned/unplanned: emergency measures | 75.90% | 1.90% |  |  |
| s9 s4ii: emergency: emergency measures that may become necessary during the procedures | 59.30% | 12.70% |  |  |
| s9 q5i: planned/unplanned: what can be done to reduce infection | 71.80% | 4.10% |  |  |
| s9 q5ii: emergency: what can be done to reduce infection | 52.10% | 18.70% |  |  |
| s9 q6i: planned/unplanned: where the scar on their skin and its appearance | 50.80% | 12.60% |  |  |
| s9 q6ii: emergency: where the scar on their skin will be | 30.20% | 33.10% |  |  |
| s9 q7ii: planned/unplanned: contraceptive or sterilisation options | 49.50% | 15.80% |  |  |
| s9 q7ii: emergency: contraceptive or sterlisation options | 23.20% | 41.60% |  |  |
| s9 q8i: planned/unplanned: how common side effects of spinal anaesthetic can be treated | 68.40% | 2.80% |  |  |
| s9 q8ii: emergency: how common side effects of spinal anaesthetic | 52.10% | 15.90% |  |  |
| s9 q9i planned/unplanned: the routine use of a urinary catheter | 69.80% | 4.10% |  |  |
| s9 q9ii: emergency: the routine use of a urinary catheter | 56.40% | 13.60% |  |  |
| s9 q10: planned/unplanned: that skin to skin and early breastfeeding | 86.30% | 1.20% |  |  |
| s9q10ii: emergency: that skin to skin and early breastfeeding | 76.20% | 4.40% |  |  |
| s10 q1i: planned/unplanned: anaesthetic options e.g. spinal | 88.90% | 0.30% |  |  |
| s10 q1ii: emergency: anaesthetic | 73.70% | 5.80% |  |  |
| s10 q2i: planned/unplanned: benefits and risks of spinal | 87.30% | 0.60% |  |  |
| s10 q2ii: emergency: benefits and risks of spinal | 70.10% | 7.30% |  |  |
| s10 q3i: planned/unplanned: benefits and risks of general a | 84.1 | 1.60% |  |  |
| s10 q3ii: emergency: benefits and risks of gen a | 70.20% | 7.30% |  |  |
| s11 q1i: planned/unplanned : how to prepare for the operation | 88.20% | 0.60% |  |  |
| s11 q1ii: emergency: how to prepare | 45.20% | 26.90% |  |  |
| s11 q2i: planned/unplanned: what to do on the day | 83.70% | 0.30% |  |  |
| s11 q2ii: emergency: what to do on the day | 45.80% | 24.10% |  |  |
| s11 q3i: planned/unplanned how consent is taken | 78.20% | 2.90% |  |  |
| s11 q3ii: emergency: how consent is taken | 67.60% | 9.90% |  |  |
| s11 q4i: planned/unplanned: what happens during the operation | 80.50% | 0.90% |  |  |
| Section 11 Q4ii: Emergency:What happens during the operatione | 56% | 10.80% |  |  |
| Section 11 Q5i: Planned/unplanned:What happens after the operation | 77.90% | 1.50% |  |  |
| Section 11 Q5ii: Emergency:What happens after the operation | 54.70% | 13.10% |  |  |
| Section 11 Q6i: Planned/unplanned:Expectations regarding vaginal bleeding | 69.20% | 2.20% |  |  |
| Section 11 Q6ii: Emergency:Expectations regarding vaginal bleeding | 52.90% | 11.90% |  |  |
| Section 11 Q7i: Planned/unplanned:Pain management | 76.30% | 1.20% |  |  |
| Section 11 Q7ii: Emergency:Pain management | 63.10% | 8.70% |  |  |
| Section 11 Q8i:Planned/unplanned:The use of blood thinning medication | 73.10% | 2.90% |  |  |
| Section 11 Q8ii: Emergency:The use of blood thinning medication | 60.20% | 10.80% |  |  |
| Section 11 Q9i: Planned/unplanned:When the catheter is removed and how long until normal bladder function usually | 66.90% | 4.50% |  |  |
| Section 11 Q9ii: Emergency:When the catheter is removed and how long until normal bladder function usually | 50.90% | 15.70% |  |  |
| Section 11 Q10i: Planned/unplanned:How long until normal bowel function usually returns | 65.40% | 6.40% |  |  |
| Section 11 Q10ii: Emergency:How long until normal bowel function usually returns( | 49% | 18% |  |  |
| Section 11 Q11i: Planned/unplanned:The usual length of time they will stay in hospital | 66.60% | 4.20% |  |  |
| Section 11 Q11ii:Emergency:The usual length of time they will stay in hospital | 51% | 17.60% |  |  |
| Section 11 Q12i: Planned/unplanned:How breastfeeding can be supported | 79.40% | 2.80% |  |  |
| Section 11 Q12ii:Emergency:How breastfeeding can be supported | 66.50% | 10.60% |  |  |
| Section 11 Q13i: Planned/unplanned:Practical aspects of longer recovery | 71.20% | 3.50% |  |  |
| Section 11 Q13ii: Emergency:Practical aspects of longer recovery | 50.40% | 16.30% |  |  |
| Section 11 Q14i: Planned/unplanned:Financial cost to health service | 15.10% | 62.50% |  |  |
| Section 11 Q14ii: Emergency:Financial cost to health service | 10.90% | 73.10% |  |  |
