# Supplementary material for: Informing Decision‐Making About Caesarean Birth: A Delphi Study to Develop a Core Information Set
Source: BJOG. 2025 Jul 8;132(13):2024–39. doi: 10.1111/1471-0528.18269 (PMC12592771; doi:10.1111/1471-0528.18269)
Supplement: Supplementary file 7 — Data S7. [file BJO-132-2024-s001.docx]

**Items rated most important by parents and health professionals in Delphi Round 2**

| **Round 2** Professionals rated nine information items to be of greatest importance (score 9). All nine were also critically important to parents. These items were; indications for emergency caesarean birth for mother (professionals median9/mean8.8-parents median9/mean8.4) and baby (professionals median9/mean8.7-parents median 9/mean8.7); for planned/unplanned caesarean birth very common complications (i.e. risk more than 1 in 10) (professionals median9/mean8.4-parents median 9/mean8.5), common complications (i.e risk bewteen 1in10 and 1in100) (professionals median9/mean8.5-parents median 9/mean8.4), significant complications during the caesarean that may require further surgery (i.e. hysterectomy, bowel) (professionals median9/mean8.3-parents median 9/mean8.3), risk of caesarean birth compared to vaginal birth (professionals median9/mean8.4-parents median 9/mean8.3), risks to baby during the operation (professionals median9/mean8.2-parents median 9/mean8.3), possibility baby may need help breathing when born (professionals median9/mean8.2-parents median 9/mean8.3), and how to prepare for the operation (i.e. when to stop eating and drinking) (professionals median9/mean8.2-parents median 9/mean8.4). Parents rated an additional six items of greatest importance. These items related to unplanned caesarean birth; indications for mother (median9/mean8.4) and baby (median9/mean8.4) and other options for birth of baby (median9/mean8.4); emergency caesarean birth other options for birth of baby (median9/mean8.2), benefits of the operation to baby (median9/mean8.4); planned/unplanned: serious conditions with short or long term risks to baby (median9/mean8.2). | | |  |
| --- | --- | --- | --- |
| Median | Mean | Item | |
| Health Professionals | | | |
| 9 | 8.8 | section 5 q 1: maternal reasons they would be advised to have an emergency caesarean birth e.g. serious concerns regarding their health | |
| 9 | 8.7 | s5 q2: reasons they would be advised to have an emergency caesarean because of concerns regardign baby | |
| 9 | 8.4 | s7 q1: planned/unplanned: very common complications (risk more than 1 in 10 | |
| 9 | 8.5 | s7 q2i: planned/unplanned common complications (risk between more than 1 in 10 and 1 in 100) | |
| 9 | 8.3 | s7 q5i: planned/ unplanned: significant complications during the caesarean birth requiring further surgery | |
| 9 | 8.4 | s7 q11i: planned/unplanned: the risk of a caesarean birth compared to vaginal birth | |
| 9 | 8.2 | s8 q1i: planned/unplanned: the risks to baby during the operation | |
| 9 | 8.2 | s8 q2i: planned/unplanned: the potential for baby to need help breathing after being born | |
| 9 | 8.2 | s11 q1i: planned/unplanned : how to prepare for the operation (when to stop eating and drinking) | |
| Parents | | | |
| 9 | 8.4 | S3; Q1reasons they may be offered an unplanned caesarean birth e.g. concerns bout how the labour is progressing, developing infection. | |
| 9 | 8.4 | r2_section3_q2 reasons a caesarean birth may be offered because of the baby e.g. concerns how baby is coping with labour | |
| 9 | 8.4 | r2_section3_q3 if there are other options for the birth of the baby depending on the circumstance | |
| 9 | 8.6 | section 5 q 1: maternal reasons they would be advised to have an emergency caesarean birth e.g. serious concerns regarding their health | |
| 9 | 8.7 | s5 q2: reasons they would be advised to have an emergency caesarean because of concerns regardign baby | |
| 9 | 8.2 | s5 q3: other options for the birth fo the baby e.g. induced birth, continuing with labour | |
| 9 | 8.4 | s6 q2: benefits of the operation to baby e.g. reduced risk of stillbirth in labour | |
| 9 | 8.5 | s7 q1: planned/unplanned: very common complications (risk more than 1 in 10 | |
| 9 | 8.4 | s7 q2i: planned/unplanned common complications (risk between more than 1 in 10 and 1 in 100) | |
| 9 | 8.3 | s7 q5i: planned/ unplanned: significant complications during the caesarean birth requiring further surgery | |
| 9 | 8.3 | s7 q11i: planned/unplanned: the risk of a caesarean birth compared to vaginal birth | |
| 9 | 8.3 | s8 q1i: planned/unplanned: the risks to baby during the operation | |
| 9 | 8.3 | s8 q2i: planned/unplanned: the potential for baby to need help breathing after being born | |
| 9 | 8.2 | s8 q4: planned/unplanned: serious conditions with short or long term risks to baby; | |
| 9 | 8.4 | s11 q1i: planned/unplanned : how to prepare for the operation (when to stop eating and drinking) | |
