# Supplementary material for: Informing Decision‐Making About Caesarean Birth: A Delphi Study to Develop a Core Information Set
Source: BJOG. 2025 Jul 8;132(13):2024–39. doi: 10.1111/1471-0528.18269 (PMC12592771; doi:10.1111/1471-0528.18269)
Supplement: Supplementary file 8 — Data S8. [file BJO-132-2024-s012.docx]

**Delphi Round 2 Results**

Summary of information item scoring for survey round 2 and inclusion or exclusion in consensus meeting

*KEY: In if either parents or professionals, consensus; Discuss if both parents and professionals, no consensus;*

*Out if either parents or professionals, consensus out.*

| **Domain** | **Information Item** | **Parents** | **Professionals** | **Included in CIS** | **Included consensus meeting** |
| --- | --- | --- | --- | --- | --- |
| Indications for planned caesarean birth | Reasons they may be offered a caesarean birth e.g. previous caesarean, placental position, twins, age, BMI | CONSENSUS | CONSENSUS | Yes | No |
|  | Reasons a caesarean birth may be offered because of the baby e.g. breech, large for dates | CONSENSUS | CONSENSUS | Yes | No |
|  | Other options for the birth of the baby e.g. spontaneous or induced birth | CONSENSUS | CONSENSUS | Yes | No |
|  | Changing their mind after deciding to have a caesarean birth | CONSENSUS | CONSENSUS | Yes | No |
|  | When they can decide to have a caesarean birth | CONSENSUS | CONSENSUS | Yes | No |
|  | What may happen if they have decided to have a caesarean and go into spontaneous labour | CONSENSUS | CONSENSUS | Yes | No |
| Benefits of planned caesarean birth | Benefits of the operation to themselves e.g. feeling in control, minimising chance of assisted vaginal birth or emergency caesarean, avoiding vaginal or perineal tears, reduced risk of urine/bowel incontinence | CONSENSUS | CONSENSUS | Yes | No |
|  | Benefits of the operation to babye.g. reduced risk of shoulders becoming stuck | CONSENSUS | CONSENSUS | Yes | No |
|  | The benefits of a caesarean birth compared to a vaginal birth | CONSENSUS | CONSENSUS | Yes | No |
| Indications for unplanned caesarean birth | Reasons they may be offered an unplanned caesarean birthe.g. if there are concerns about how the labour is progressing, developing infection | CONSENSUS | CONSENSUS | Yes | No |
|  | Reasons a caesarean birth may be offered because of the babye.g. there are concerns with how your baby is coping with labour | CONSENSUS | CONSENSUS | Yes | No |
|  | If there are other options for the birth of the baby depending on the circumstancese.g. induced birth, continuing with labour | CONSENSUS | CONSENSUS | Yes | No |
|  | Changing their mind after deciding to have a caesarean birth | CONSENSUS | CONSENSUS | Yes | No |
|  | When they can decide to have a caesarean birth | CONSENSUS | CONSENSUS | Yes | No |
|  | What will happen if they decided to have a caesarean and then go into spontaneous labour | CONSENSUS | CONSENSUS | Yes | No |
| Benefits of unplanned caesarean birth | Benefits of the operation to themselves e.g. reduced risk of vaginal or perineal tearing, treatment of developing conditions, minimising chance of assisted vaginal birth | CONSENSUS | CONSENSUS | Yes | No |
|  | Benefits of the operation to babye.g. less exposure to developing stress of infection | CONSENSUS | CONSENSUS | Yes | No |
| Indications for emergency caesarean birth | Maternal reasons they would be advised to have an emergency caesarean birthe.g. serious concerns regarding their health or developing conditions | CONSENSUS | CONSENSUS | Yes | No |
|  | Reasons they would be advised to have an emergency caesarean because of concerns regarding babye.g. serious and time critical concerns for the health of the baby | CONSENSUS | CONSENSUS | Yes | No |
|  | Other options for the birth of the babye.g. induced birth, continuing with labour | CONSENSUS | CONSENSUS | Yes | No |
| Benefits of emergency caesarean birth | Benefits of the operation to themselvese.g. control of bleeding or infection, reduced risk of vaginal or perineal tearing, minimising chance of assisted vaginal birth | CONSENSUS | CONSENSUS | Yes | No |
|  | Benefits of the operation to babye.g. reduced risk of stillbirth in labour | CONSENSUS | CONSENSUS | Yes | No |
| Risks to pregnant woman | Planned/unplanned: Very common complications (risk more than 1 in 10)e.g. urinary incontinence | CONSENSUS | CONSENSUS | Yes | No |
|  | Emergency: Very common complications (risk more than 1 in 10)e.g. urinary incontinence | CONSENSUS | CONSENSUS | Yes | No |
|  | Planned/unplanned:Common complications (risk between 1 and 10 and 1 in 100)e.g. wound infection/breakdown, infection of the womb lining, womb rupture in future pregnancy | CONSENSUS | CONSENSUS | Yes | No |
|  | Emergency: Common complications (risk between 1 and 10 and 1 in 100)e.g. wound infection/breakdown, infection of the womb lining, womb rupture in future pregnancy | CONSENSUS | CONSENSUS | Yes | No |
|  | Planned/unplanned:Uncommon complications (risk between 1 in 100 and 1 in 1,000)e.g. excessive bleeding requiring blood transfusion, blood clots in legs or lung (deep vein thrombosis or pulmonary embolism), urinary or intestinal injury, hysterectomy | CONSENSUS | CONSENSUS | Yes | No |
|  | Emergency:Uncommon complications (risk between 1 in 100 and 1 in 1,000)e.g. excessive bleeding requiring blood transfusion, blood clots in legs or lung (deep vein thrombosis or pulmonary embolism), urinary or intestinal injury, hysterectomy | NO CONSENSUS | CONSENSUS | Yes | No |
|  | Planned/unplanned:Rare complications (risk between 1 in 1,000 and 1 in 10,000) e.g. death | CONSENSUS | CONSENSUS | Yes | No |
|  | Emergency:Rare complications (risk between 1 in 1,000 and 1 in 10,000) e.g. death | NO CONSENSUS | NO CONSENSUS | No Consensus | Yes |
|  | Planned/unplanned:Significant complications during the caesaerean birth requiring further surgery e.g. hysterectomy, bowel damage, urine system damage | CONSENSUS | CONSENSUS | Yes | No |
|  | Emergency:Significant complications during the caesaerean birth requiring further surgery e.g. hysterectomy, bowel damage, urine system damage | CONSENSUS | CONSENSUS | Yes | No |
|  | Planned/unplanned:Serious illness during or after birth that may result in long-term hospital admission or consequencese.g. admission to ICU, sepsis, blood clots in legs or lungs (deep vein thrombosis or pulmonary embolism), the need for future operations | CONSENSUS | CONSENSUS | Yes | No |
|  | Emergency:Serious illness during or after birth that may result in long-term hospital admission or consequencese.g. admission to ICU, sepsis, blood clots in legs or lungs (deep vein thrombosis or pulmonary embolism), the need for future operations | CONSENSUS | CONSENSUS | Yes | No |
|  | Planned/unplanned:The effects of birth by caesarean on future pregnancies e.g. low lying placenta (where the placenta blocks the exit of the womb), invasive placenta (where the placenta invades the wall of the womb), ectopic pregnancy (pregnancy outside of the womb), womb rupture (where a hole forms in the womb), stillbirth | CONSENSUS | CONSENSUS | Yes | No |
|  | Emergency:The effects of birth by caesarean on future pregnancies e.g. low lying placenta (where the placenta blocks the exit of the womb), invasive placenta (where the placenta invades the wall of the womb), ectopic pregnancy (pregnancy outside of the womb), womb rupture (where a hole forms in the womb), stillbirth | NO CONSENSUS | NO CONSENSUS | No Consensus | Yes |
|  | Planned/unplanned:The risk of future pelvic floor related problemse.g. pelvic organ prolapse, inability to control bladder or bowels | CONSENSUS | CONSENSUS | Yes | No |
|  | Emergency:The risk of future pelvic floor related problemse.g. pelvic organ prolapse, inability to control bladder or bowels | NO CONSENSUS | NO CONSENSUS | No Consensus | Yes |
|  | Planned/unplanned:The psychological effects of birth (especially unplanned mode of delivery)e.g. on quality of life, post-traumatic stress disorder (PTSD), negative birth experience, postnatal depression | CONSENSUS | CONSENSUS | Yes | No |
|  | Emergency:The psychological effects of birth (especially unplanned mode of delivery)e.g. on quality of life, post-traumatic stress disorder (PTSD), negative birth experience, postnatal depression | NO CONSENSUS | NO CONSENSUS | No Consensus | Yes |
|  | Planned/unplanned:The likelihood of pain after a caesarean birth and how long it may last for | CONSENSUS | CONSENSUS | Yes | No |
|  | Emergency:The likelihood of pain after a caesarean birth and how long it may last for | NO CONSENSUS | NO CONSENSUS | No Consensus | Yes |
|  | Planned/unplanned:The risk of a caesarean birth compared to vaginal birth | CONSENSUS | CONSENSUS | Yes | No |
|  | Emergency:The risk of a caesarean birth compared to vaginal birth | NO CONSENSUS | CONSENSUS | Yes | No |
| Risks to the baby | Planned/unplanned: The risks to baby during the operatione.g. cut to baby's skin, difficulty or injury during delivery of baby | CONSENSUS | CONSENSUS | Yes | No |
|  | Emergency: The risks to baby during the operatione.g. cut to baby's skin, difficulty or injury during delivery of baby | CONSENSUS | CONSENSUS | Yes | No |
|  | Planned/unplanned:The potential for baby to need help breathing after being borne.g. oxygen, ventilation, resuscitation | CONSENSUS | CONSENSUS | Yes | No |
|  | Emergency:The potential for baby to need help breathing after being borne.g. oxygen, ventilation, resuscitation | CONSENSUS | CONSENSUS | Yes | No |
|  | Planned/unplanned:The potential for baby to need admission to the neonatal intensive care unit for extra care and how long this admission may be needed | CONSENSUS | CONSENSUS | Yes | No |
|  | Emergency:The potential for baby to need admission to the neonatal intensive care unit for extra care and how long this admission may be needed | CONSENSUS | CONSENSUS | Yes | No |
|  | Planned/unplanned:Serious conditions with short or long term risks to baby after birthe.g. infection (may need antibiotics), low blood sugar, seizures, brain injury, organ failure, stillbirth, neonatal death | CONSENSUS | CONSENSUS | Yes | No |
|  | Emergency:Serious conditions with short or long term risks to baby after birthe.g. infection (may need antibiotics), low blood sugar, seizures, brain injury, organ failure, stillbirth, neonatal death | CONSENSUS | CONSENSUS | Yes | No |
|  | Planned/unplanned:Long term conditions that may be associated with caesarean birth to the babye.g. asthma, type 1 diabetes, obesity, immune disorders | CONSENSUS | NO CONSENSUS | Yes | No |
|  | Emergency:Long term conditions that may be associated with caesarean birth to the babye.g. asthma, type 1 diabetes, obesity, immune disorders | NO CONSENSUS | NO CONSENSUS | No Consensus | Yes |
| Practicalities | Planned/unplanned:How the operation is performed (including possible variations and their reasons and effects) | CONSENSUS | NO CONSENSUS | Yes | No |
|  | Emergency:How the operation is performed (including possible variations and their reasons and effects) | NO CONSENSUS | NO CONSENSUS | No Consensus | Yes |
|  | Planned/unplanned:How long the operation usually takes | NO CONSENSUS | NO CONSENSUS | No Consensus | Yes |
|  | Emergency:How long the operation usually takes | NO CONSENSUS | NO CONSENSUS | No Consensus | Yes |
|  | Planned/unplanned:How bleeding is routinely managede.g. through the use of medications to help the womb contract (oxytocin) | NO CONSENSUS | NO CONSENSUS | No Consensus | Yes |
|  | Emergency:How bleeding is routinely managede.g. through the use of medications to help the womb contract (oxytocin) | NO CONSENSUS | NO CONSENSUS | No Consensus | Yes |
|  | Planned/unplanned:Emergency measures that may become necessary during the proceduree.g. use of forceps to deliver baby, other ways to control bleeding including further surgery | CONSENSUS | NO CONSENSUS | Yes | No |
|  | Emergency:Emergency measures that may become necessary during the proceduree.g. use of forceps to deliver baby, other ways to control bleeding including further surgery | NO CONSENSUS | NO CONSENSUS | No Consensus | Yes |
|  | Planned/unplanned:What can be done to reduce infectione.g. routine use of antibiotics prior to birth, vaginal cleaning prior to the operation starting | CONSENSUS | NO CONSENSUS | Yes | No |
|  | Emergency:What can be done to reduce infectione.g. routine use of antibiotics prior to birth, vaginal cleaning prior to the operation starting | NO CONSENSUS | NO CONSENSUS | No Consensus | Yes |
|  | Planned/unplanned:Where the scar on their skin will be and it's appearance | NO CONSENSUS | NO CONSENSUS | No Consensus | Yes |
|  | Emergency:Where the scar on their skin will be and it's appearance | NO CONSENSUS | NO CONSENSUS | No Consensus | Yes |
|  | Planned/unplanned:Contraceptive or sterilisation options that can be performed during the operatione.g. fitting of contraceptive coil, tube tying/removal | NO CONSENSUS | NO CONSENSUS | No Consensus | Yes |
|  | Emergency:Contraceptive or sterilisation options that can be performed during the operatione.g. fitting of contraceptive coil, tube tying/removal | NO CONSENSUS | NO CONSENSUS | No Consensus | Yes |
|  | Planned/unplanned:How common side effects of spinal anaesthetic can be treated during the operatione.g. nausea/vomiting or shivering | NO CONSENSUS | NO CONSENSUS | No Consensus | Yes |
|  | Emergency:How common side effects of spinal anaesthetic can be treated during the operatione.g. nausea/vomiting or shivering | NO CONSENSUS | NO CONSENSUS | No Consensus | Yes |
|  | The routine use of a urinary catheter to protect your bladder | CONSENSUS | CONSENSUS | Yes | No |
|  | Emergency:The routine use of a urinary catheter to protect your bladder | NO CONSENSUS | CONSENSUS | Yes | No |
|  | Planned/unplanned:That skin to skin and early breastfeeding can usually be facilitated | CONSENSUS | CONSENSUS | Yes | No |
|  | Emergency:That skin to skin and early breastfeeding can usually be facilitated | CONSENSUS | NO CONSENSUS | Yes | No |
| Anaesthetic options | Planned/unplanned:Anaesthetic optionse.g. spinal (an injection into the back to numb from the chest down) or general anaesthetic (being put to sleep for the operation) | CONSENSUS | CONSENSUS | Yes | No |
|  | Emergency:Anaesthetic optionse.g. spinal (an injection into the back to numb from the chest down) or general anaesthetic (being put to sleep for the operation) | CONSENSUS | CONSENSUS | Yes | No |
|  | Planned/unplanned:Benefits and risks of spinal anaesthetic | CONSENSUS | CONSENSUS | Yes | No |
|  | Emergency:Benefits and risks of spinal anaesthetic | NO CONSENSUS | NO CONSENSUS | No Consensus | Yes |
|  | Planned/unplanned:Benefits and risks of general anaesthetic | CONSENSUS | CONSENSUS | Yes | No |
|  | Emergency:Benefits and risks of general anaesthetic | NO CONSENSUS | NO CONSENSUS | No Consensus | Yes |
| Patient experience and recovery | Planned/unplanned:How to prepare for the operatione.g. when to stop eating and drinking, taking an antacid | CONSENSUS | CONSENSUS | Yes | No |
|  | Emergency:How to prepare for the operatione.g. when to stop eating and drinking, taking an antacid | NO CONSENSUS | NO CONSENSUS | No Consensus | Yes |
|  | Planned/unplanned:What to do on the daye.g. where and when to attend, presence of birth partners | CONSENSUS | CONSENSUS | Yes | No |
|  | Emergency:What to do on the daye.g. where and when to attend, presence of birth partners | NO CONSENSUS | NO CONSENSUS | No Consensus | Yes |
|  | Planned/unplanned:How consent is takene.g. written or verbal | CONSENSUS | CONSENSUS | Yes | No |
|  | Emergency:How consent is takene.g. written or verbal | NO CONSENSUS | NO CONSENSUS | No Consensus | Yes |
|  | Planned/unplanned:What happens during the operatione.g. medical professionals who may be present and why, where birth partner sits, music, when and how they can meet the baby | CONSENSUS | CONSENSUS | Yes | No |
|  | Emergency:What happens during the operatione.g. medical professionals who may be present and why, where birth partner sits, music, when and how they can meet the baby | NO CONSENSUS | NO CONSENSUS | No Consensus | Yes |
|  | What happens after the operation e.g. how long in recovery prior to moving to the ward, eating and drinking, walking, showering, dressing removal, catheter removal | CONSENSUS | CONSENSUS | Yes | No |
|  | Emergency:What happens after the operatione.g. how long in recovery prior to moving to the ward, eating and drinking, walking, showering, dressing removal, catheter removal | NO CONSENSUS | NO CONSENSUS | No Consensus | Yes |
|  | Planned/unplanned:Expectations regarding vaginal bleeding after a caesarean section | CONSENSUS | NO CONSENSUS | Yes | No |
|  | Emergency:Expectations regarding vaginal bleeding after a caesarean section | NO CONSENSUS | NO CONSENSUS | No Consensus | Yes |
|  | Planned/unplanned:Pain management both whilst in hospital and at home | CONSENSUS | CONSENSUS | Yes | No |
|  | Emergency:Pain management both whilst in hospital and at home | NO CONSENSUS | NO CONSENSUS | No Consensus | Yes |
|  | Planned/unplanned:The use of blood thinning medication to reduce the risk of blood clots in legs and lungs (deep vein thrombosis or pulmonary embolism) after birth | CONSENSUS | CONSENSUS | Yes | No |
|  | Emergency:The use of blood thinning medication to reduce the risk of blood clots in legs and lungs (deep vein thrombosis or pulmonary embolism) after birth | NO CONSENSUS | CONSENSUS | Yes | No |
|  | Planned/unplanned:When the catheter is removed and how long until normal bladder function usually returns | CONSENSUS | NO CONSENSUS | Yes | No |
|  | Emergency:When the catheter is removed and how long until normal bladder function usually returns | NO CONSENSUS | NO CONSENSUS | No Consensus | Yes |
|  | Planned/unplanned:How long until normal bowel function usually returns | NO CONSENSUS | NO CONSENSUS | No Consensus | Yes |
|  | Emergency:How long until normal bowel function usually returns | NO CONSENSUS | NO CONSENSUS | No Consensus | Yes |
|  | Planned/unplanned:The usual length of time they will stay in hospital | NO CONSENSUS | CONSENSUS | Yes | No |
|  | Emergency:The usual length of time they will stay in hospital | NO CONSENSUS | NO CONSENSUS | No Consensus | Yes |
|  | Planned/unplanned:How breastfeeding can be supported | CONSENSUS | CONSENSUS | Yes | No |
|  | Emergency:How breastfeeding can be supported | NO CONSENSUS | NO CONSENSUS | No Consensus | Yes |
|  | Planned/unplanned:Practical aspects of longer recoverye.g. driving, heavy lifting, exercise, sex and contraception | CONSENSUS | CONSENSUS | Yes | No |
|  | Emergency:Practical aspects of longer recoverye.g. driving, heavy lifting, exercise, sex and contraception | NO CONSENSUS | NO CONSENSUS | No Consensus | Yes |
|  | Planned/unplanned:Financial cost to health service e.g. length of stay, need for re-attendance to medical providers, need for further treatment | NO CONSENSUS | NO CONSENSUS | No Consensus | Yes |
|  | Emergency:Financial cost to health service e.g. length of stay, need for re-attendance to medical providers, need for further treatment | NO CONSENSUS | NO CONSENSUS | No | No |
| Additional questions | Planned/unplanned:Caesarean scar pain in the short and long term | NO CONSENSUS | NO CONSENSUS | No Consensus | Yes |
|  | Emergency:Caesarean scar pain in the short and long term | NO CONSENSUS | NO CONSENSUS | No Consensus | Yes |
|  | Emergency:The fact that someone will come and discuss with you why you needed the operation and any important longer-term considerations | CONSENSUS | CONSENSUS | Yes | No |
