# Supplementary material for: Informing Decision‐Making About Caesarean Birth: A Delphi Study to Develop a Core Information Set
Source: BJOG. 2025 Jul 8;132(13):2024–39. doi: 10.1111/1471-0528.18269 (PMC12592771; doi:10.1111/1471-0528.18269)
Supplement: Supplementary file 9 — Data S9. [file BJO-132-2024-s011.docx]

**Consensus meeting antenatal caesarean birth core information set items**

|  | **Item number(s) merged** | **Delphi order 1** | **Final order** | **Why a caesarean birth may be offered?** |
| --- | --- | --- | --- | --- |
| 1 | 1 |  |  | *Reasons they may be offered a caesarean birth e.g. previous caesarean, placental position, twins, age, BMI (planned caesarean birth)* |
| 2 | 10,18 |  |  | *Reasons they may be offered an unplanned caesarean birth e.g. if there are concerns about how the labour is progressing, developing infection (unplanned caesarean birth, emergency)* |
| 3 | 2 |  |  | *Reasons a caesarean birth may be offered because of the baby e.g. breech, large for dates​ (planned caesarean birth)* |
| 4 | 11,19 |  |  | *Reasons a caesarean birth may be offered because of the baby e.g. there are concerns with how your baby is coping with labour (unplanned caesarean birth, emergency caesarean)* |
|  |  | **2** | **2** | **What are the other options for the birth of your baby?** |
| 5 | 3 |  |  | *Other options for the birth of the baby e.g. spontaneous or induced birth (planned caesarean)* |
| 6 | 20 |  |  | *If there are other options for the birth of the baby depending on the circumstances e.g. induced birth, continuing with labour (unplanned caesarean birth, emergency caesarean birth)* |
|  |  | **3** | **3** | **What if things change?** |
| 7 | 4 |  |  | Changing their mind after deciding to have a caesarean birth (planned CS)​ |
| 8 | 13 |  |  | Changing their mind after deciding to have a caesarean birth (unplanned CS)​ |
| 9 | 5 |  |  | When they can decide to have a caesarean birth (planned CS)​ |
| 10 | 14 |  |  | When they can decide to have a caesarean birth (unplanned CS)​ |
| 11 | 6 |  |  | What may happen if they have decided to have a caesarean and go into spontaneous labour (planned CS) |
| 12 | 15 |  |  | What may happen if they have decided to have a caesarean and go into spontaneous labour (unplanned CS) |
|  |  | **4** | **4** | **Benefits of caesarean birth? ​** |
| 13 | 7 |  |  | Benefits of the operation to themselves e.g. feeling in control, minimising chance of assisted vaginal birth or emergency caesarean, avoiding vaginal or perineal tears, reduced risk of urine/bowel incontinence |
| 14 | 8 |  |  | Benefits of the operation to baby e.g. reduced risk of shoulders becoming stuck​ |
| 15 | 16,21 |  |  | Benefits of the operation to themselves e.g. reduced risk of vaginal or perineal tearing, treatment of developing conditions, minimising chance of assisted vaginal birth |
| 16 | 17,22 |  |  | Benefits of the operation to baby e.g. less exposure to developing stress of infection, stillbirth |
| 17 | 67,68 |  |  | Contraceptive or sterilisation options that can be performed during the operation e.g. fitting of contraceptive coil, tube tying/removal |
|  |  | **5** | **9** | **Comparison on Caesarean and Vaginal Birth** |
| 18 | 43,44 |  |  | The risk of a caesarean birth compared to vaginal birth |
| 19 | 9 |  |  | The benefits of a caesarean birth compared to a vaginal birth |
|  |  | **6** | **11** | **What to expect during a Caesarean Birth​** |
| 20 | 55,56 |  |  | How the operation is performed (including possible variations and their reasons and effects)​ |
| 21 | 57,58 |  |  | How long the operation usually takes​ |
| 22 | 59,60 |  |  | How bleeding is routinely managed e.g. through the use of medications to help the womb contract (oxytocin)​ |
| 23 | 71,70 |  |  | The routine use of a urinary catheter to protect your bladder​ |
| 24 | 61,62 |  |  | Emergency measures that may become necessary during the procedure e.g. use of forceps to deliver baby, other ways to control bleeding including further surgery​ |
| 25 | 63,64 |  |  | What can be done to reduce infection e.g. routine use of antibiotics prior to birth, vaginal cleaning prior to the operation starting​ |
| 26 | 73,74 |  |  | That skin to skin and early breastfeeding can usually be facilitated​ |
| 27 | 65,66 |  |  | Where the scar on their skin will be and its appearance​ |
|  |  | **7** | **8** | **Anaesthetic Options** |
| 28 | 75,76 |  |  | Anaesthetic options e.g. spinal (an injection into the back to numb from the chest down) or general anaesthetic (being put to sleep for the operation)​ |
| 29 | 77,78 |  |  | Benefits and risks of spinal anaesthetic​ |
| 30 | 79,80 |  |  | Benefits and risks of general anaesthetic​ |
| 31 | 69,70 |  |  | How common side effects of spinal anaesthetic can be treated during the operation e.g. nausea/vomiting or shivering |
|  |  | **8** | **10** | **Preparing for your Caesarean Birth** |
| 32 | 81,82 |  |  | How to prepare for the operation e.g. when to stop eating and drinking, taking an antacid​ |
| 33 | 83,84 |  |  | What to do on the day e.g. where and when to attend, presence of birth partners​ |
| 34 | 85,86 |  |  | How consent is taken e.g. written or verbal​ |
| 35 | 87,88 |  |  | What happens during the operation e.g. medical professionals who may be present and why, where birth partner sits, music, when and how they can meet the baby​ |
|  |  | **9** | **5** | **Risks at time of operation for mother ?** |
| 36 | 23,24 |  |  | Very common complications (risk more than 1 in 10)e.g. urinary incontinence​ |
| 37 | 25,26 |  |  | Common complications (risk between 1 and 10 and 1 in 100)e.g. wound infection/breakdown, infection of the womb lining |
| 38 | 27,28 |  |  | Uncommon complications (risk between 1 in 100 and 1 in 1,000)e.g. excessive bleeding requiring blood transfusion, blood clots in legs or lung (deep vein thrombosis or pulmonary embolism), urinary or intestinal injury, hysterectomy​ |
| 39 | 29,30 |  |  | Rare complications (risk between 1 in 1,000 and 1 in 10,000) e.g. death​ |
| 40 | 31,32 |  |  | Significant complications during the caesarean birth requiring further surgery e.g. hysterectomy, bowel damage, urine system damage​ |
| 41 | 33,34 |  |  | Serious illness during or after birth that may result in long-term hospital admission or consequences e.g. admission to ICU, sepsis, blood clots in legs or lungs (deep vein thrombosis or pulmonary embolism), the need for future operations |
|  |  | **10** | **6** | **Risk at time of operation for baby​** |
| 42 | 45,46 |  |  | The risks to baby during the operation e.g. cut to baby's skin, difficulty or injury during delivery of baby​ |
| 43 | 47,48 |  |  | The potential for baby to need help breathing after being born e.g. oxygen, ventilation, resuscitation​ |
| 44 | 49,50 |  |  | The potential for baby to need admission to the neonatal intensive care unit for extra care and how long this admission may be needed |
|  |  | **11** | **7** | **Risks following the operation** |
| 45 | 37,38 |  |  | The risk of future pelvic floor related problems e.g. pelvic organ prolapse, inability to control bladder or bowels​ |
| 46 | 39,40 |  |  | The psychological effects of birth (especially unplanned mode of delivery)e.g. on quality of life, post-traumatic stress disorder (PTSD), negative birth experience, postnatal depression​ |
| 47 | 51,52 |  |  | Serious conditions with short or long term risks to baby after birth e.g. infection (may need antibiotics), low blood sugar, seizures, brain injury, organ failure, stillbirth, neonatal death​ |
| 48 | 53,54 |  |  | Long term conditions that may be associated with caesarean birth to the baby e.g. asthma, type 1 diabetes, obesity, immune disorders​ |
|  |  | **12** | **14** | **Future pregnancies following a caesarean birth​** |
| 49 | 35,36 |  |  | The effects of birth by caesarean on future pregnancies e.g. low lying placenta (where the placenta blocks the exit of the womb), invasive placenta (where the placenta invades the wall of the womb), ectopic pregnancy (pregnancy outside of the womb), womb rupture (where a hole forms in the womb), stillbirth |
|  |  | **13** | **12** | **Recovering after a caesarean birth​** |
| 50 | 99,100 |  |  | How long until normal bowel function usually returns​ |
| 51 | 91,92 |  |  | Expectations regarding vaginal bleeding after a caesarean section​ |
| 52 | 93,94 |  |  | Pain management both whilst in hospital and at home​ |
| 53 | Round 2 |  |  | Caesarean scar - pain in the short term |
|  |  | **14** | **13** | **What to expect following a caesarean birth** |
| 54 | 89,90 |  |  | What happens after the operation e.g. how long in recovery prior to moving to the ward, eating and drinking, walking, showering, dressing removal, catheter removal​ |
| 55 | 105, 106 |  |  | Practical aspects of longer recovery e.g.  driving, heavy lifting, exercise, sex and contraception​ |
| 56 | 101,102 |  |  | The usual length of time they will stay in hospital​ |
| 57 | 97,98 |  |  | When the catheter is removed and how long until normal bladder function usually returns​ |
| 58 | 95,96 |  |  | The use of blood thinning medication to reduce the risk of blood clots in legs and lungs (deep vein thrombosis or pulmonary embolism) after birth​ |
| 59 | 103,104 |  |  | How breastfeeding can be supported​ |
| 61 | NEW |  |  | How formula feeding can be supported​ |
|  | 99,100 |  |  | How long until normal bowel function usually returns​ |
|  | 91,92 |  |  | Expectations regarding vaginal bleeding after a caesarean section​ |
|  | 93,94 |  |  | Pain management both whilst in hospital and at home​ |
|  | Round 2 |  |  | Caesarean scar pain - in the long term |
| 60 | 41,42 |  |  | The likelihood of pain after a caesarean birth and how long it may last for​ |
|  | Round 2 |  |  | PN set |
|  | 107,108 |  |  | Financial cost - removed |
