# Supplementary material for: Informing Decision‐Making About Caesarean Birth: A Delphi Study to Develop a Core Information Set
Source: BJOG. 2025 Jul 8;132(13):2024–39. doi: 10.1111/1471-0528.18269 (PMC12592771; doi:10.1111/1471-0528.18269)
Supplement: Supplementary file 10 — Data S10. [file BJO-132-2024-s007.docx]

**Consensus meeting emergency caesarean birth core information set items**

|  | **1. Why a caesarean is being advised and for what reason** |
| --- | --- |
| 1 | *Reasons they may be offered an unplanned caesarean birth e.g. if there are concerns about how the labour is progressing, developing infection (unplanned caesarean birth, emergency)* |
| 2 | *Reasons a caesarean birth may be offered because of the baby e.g. there are concerns with how your baby is coping with labour (unplanned caesarean birth, emergency caesarean)* |
|  | **2. Any risks to the baby and whether it is anticipated that the baby may need immediate help breathing, admission to the neonatal unit** |
| **3** | The potential for baby to need admission to the neonatal intensive care unit for extra care and how long this admission may be needed |
|  | **3. Significant complications during the caesarean birth requiring further surgery** |
| 4 | Significant complications during the caesarean birth requiring further surgery e.g. hysterectomy, bowel damage, urine system damage​ |
|  | **4. Emergency measures that may become necessary during the procedure** |
| 5 | Emergency measures that may become necessary during the procedure e.g. use of forceps to deliver baby, other ways to control bleeding including further surgery​ |
|  | **5. Anaesthetic options** |
| 6 | Anaesthetic options e.g. spinal (an injection into the back to numb from the chest down) or general anaesthetic (being put to sleep for the operation)​ |
