# Supplementary material for: Informing Decision‐Making About Caesarean Birth: A Delphi Study to Develop a Core Information Set
Source: BJOG. 2025 Jul 8;132(13):2024–39. doi: 10.1111/1471-0528.18269 (PMC12592771; doi:10.1111/1471-0528.18269)
Supplement: Supplementary file 11 — Data S11. [file BJO-132-2024-s003.docx]

**Consensus meeting postnatal caesarean birth core information set items**

This set includes items from the long-list and arises from an unmet need identified in the Delphi survey. Round 2 of the Delphi survey had the question/item - Emergency: The fact that someone will come and discuss with you why you needed the operation and any important longer-term considerations.

| **Domain** | **Items** | **Information** |
| --- | --- | --- |
| **1** |  | **Why caesarean birth was offered** |
| **2** |  | **How common caesarean birth is** |
| **3** |  | **Were there any other realistic options for you for your birth** |
| **4** |  | **Were there any other realistic options for your baby for the birth** |
| **5** |  | **How the caesarean birth went** |
|  | 1 | How the operation was performed (including possible variations and their reasons and effects) |
|  | 2 | How long the operation took |
|  | 3 | How bleeding was managed e.g. through the use of medications to help the womb contract (oxytocin) |
|  | 4 | The presence of a urinary catheter to protect your bladder |
|  | 5 | Emergency measures that were necessary during the procedure (e.g. the use of forceps to deliver the baby, other ways to control bleeding including further surgery |
|  | 6 | What was done to reduce infection (e.g. routine use of antibiotics prior to birth, vaginal cleaning prior to the operation starting |
|  | 7 | Where the scar on their skin is |
| **6** |  | **Anaesthetic information** |
|  | 8 | Anaesthetic used |
|  | 9 | Common side effects of anaesthetic they may experience |
| **7** |  | **Any complications at the time of the operation for the mother** |
| **8** |  | **Any complications at the time of the operation for the baby** |
| **9** |  | **Risks following the operation** |
|  | 10 | The risk of future pelvic floor related problems e.g. pelvic organ prolapse, inability to control bladder or bowels |
|  | 11 | The psychological effects of birth (especially unplanned mode of delivery) e.g. on quality of life, post-traumatic stress disorder (PTSD), negative birth experience, postnatal depression |
|  | 12 | Serious conditions with short or long-term risks to baby after birth e.g. infection (may need antibiotics, low blood sugar, seizures, brain injury, organ failure, stillbirth, neonatal death |
|  | 13 | Long term conditions that may be associated with caesarean birth to the baby e.g. asthma, type 1 diabetes, obesity, immune disorders |
| **10** |  | **What to expect following a caesarean birth** |
|  | 14 | What happened after the operation e.g. how long in recovery prior to moving to the ward, eating and drinking, walking, showering, dressing removal, catheter removal |
|  | 15 | Practical aspects of longer recovery e.g.  driving, heavy lifting, exercise, sex and contraception​ |
|  | 16 | The usual length of time they will stay in hospital​ |
|  | 17 | When the catheter is removed and how long until normal bladder function usually returns​ |
|  | 18 | The use of blood thinning medication to reduce the risk of blood clots in legs and lungs (deep vein thrombosis or pulmonary embolism) after birth​ |
|  | 19 | How breastfeeding can be supported​ |
|  | 20 | How formula feeding can be supported​ |
|  | 21 | How long until normal bowel function usually returns​ |
|  | 22 | Expectations regarding vaginal bleeding after a caesarean section​ |
|  | 23 | Pain management both whilst in hospital and at home​ |
|  | 24 | Caesarean scar pain in the short and long term |
|  | 25 | The likelihood of pain after a caesarean birth and how long it may last for​ |
| **11** |  | **Recovering after a caesarean birth** |
|  | 26 | How long until normal bowel function usually returns​ |
|  | 27 | Expectations regarding vaginal bleeding after a caesarean section​ |
|  | 28 | Pain management both whilst in hospital and at home​ |
|  | 29 | Caesarean scar pain in the short and long term​ |
| **12** |  | **Future pregnancies following a caesarean birth​** |
|  | 30 | VBAC and success chances |
|  | 31 | The effects of birth by caesarean on future pregnancies e.g. low lying placenta (where the placenta blocks the exit of the womb), invasive placenta (where the placenta invades the wall of the womb), ectopic pregnancy (pregnancy outside of the womb), womb rupture (where a hole forms in the womb), stillbirth, |
