# Supplementary material for: Informing Decision‐Making About Caesarean Birth: A Delphi Study to Develop a Core Information Set
Source: BJOG. 2025 Jul 8;132(13):2024–39. doi: 10.1111/1471-0528.18269 (PMC12592771; doi:10.1111/1471-0528.18269)
Supplement: Supplementary file 12 — Data S12. [file BJO-132-2024-s010.pdf]

Caesarean Birth Core Information Set

Birth Options

What is a core information set?

A core information set is the information everyone needs before making a decision about their care. They do not replace personalised discussions. The Birth Options core information sets have been made for families and healthcare professionals to use to provide information to support decisions about birth. Women, birthing people, partners, midwives and doctors have decided which information is most important.

This information is intended as a guide and uses evidence from national guidelines, national statistics and research studies. It includes some more general information that explains usual practice. It has the best available information at the time that it was made (2025).

This information is about caesarean birth. It is most useful when you have time to discuss a caesarean, usually antenatally.

There is an **emergency caesarean birth core information set** for when delivery is within 30 minutes and there is little time for discussion. If you have an unplanned/emergency caesarean there is a **postnatal core information set** to inform discussions before you leave hospital.

Caesarean birth information sets do not include much information about induction of labour, spontaneous vaginal birth or instrumental vaginal birth. Other core information sets are available for **induction of labour**, and **vaginal birth.**

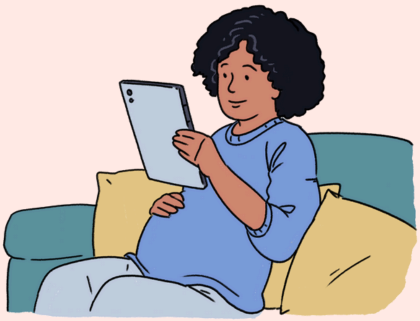

www.birthoptions.co.uk

FUNDED BY NIHR | National Institute for Health and Care Research

UNIVERSITY OF LIVERPOOL

NHS Liverpool Women's NHS Foundation Trust

What is a caesarean birth?

Birth Options

A caesarean is an operation where your baby is born through a cut made in your abdomen and womb.

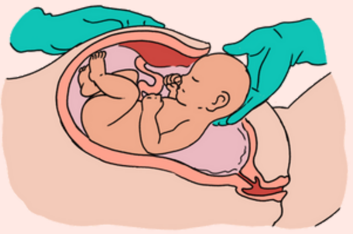

When is caesarean birth an option?

Caesarean birth can be an option before or during labour.
There are different levels of urgency for a caesarean birth:

- *Planned caesarean birth.*
- *Unplanned caesarean birth (with time to discuss options within 72 hours).*
- *Emergency caesarean birth (can be within 30 minutes).*

How common are caesareans?

How likely you are to have a caesarean birth depends on your current pregnancy, previous birth experience and personal preferences. In England, the total caesarean birth rate for all births is **42 in 100.**

44 in 100 women/birthing people have a caesarean birth in their first pregnancy.

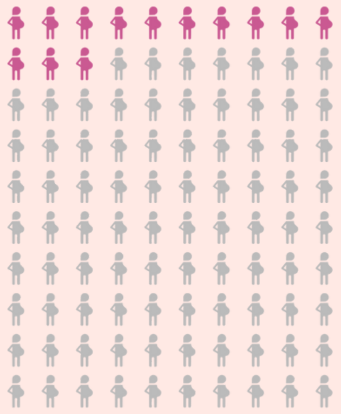

13 in 100 have a planned caesarean birth

And

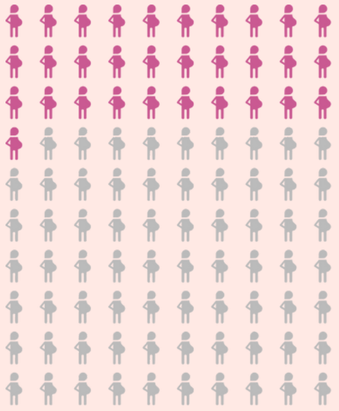

31 in 100 have an unplanned/ emergency caesarean birth

If they have had a vaginal birth before, it is less common to have an unplanned/emergency caesarean birth.

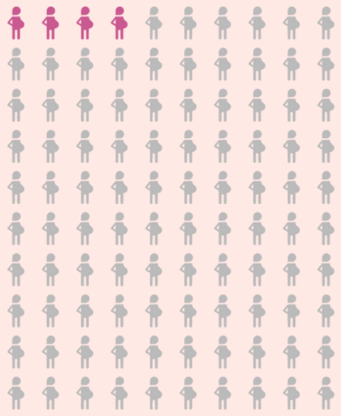

4 in 100 have an unplanned caesarean birth

FUNDED BY NIHR | National Institute for Health and Care Research

UNIVERSITY OF LIVERPOOL

NHS Liverpool Women's NHS Foundation Trust

Why a caesarean birth may be offered?

Birth Options

Planned caesarean birth

Can be scheduled before labour following a discussion with a senior midwife or doctor.

It can be offered or requested for lots of reasons including:

- The position of your baby e.g. Baby being bottom first (Breech).
- You have had a previous caesarean birth (especially if more than one previous caesarean).
- You are expecting twins that might need a caesarean birth.
- Some medical problems e.g. infection.
- Low lying (placenta praevia) or invasive placenta (placenta accreta).
- You request it.

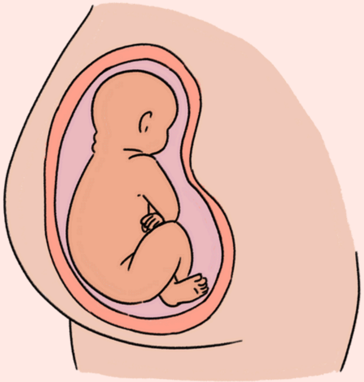

Baby in breech position

Unplanned caesarean birth

Can be offered because:

- Your baby shows signs of not coping with labour.
- There are concerns for your health (e.g. infection) before or during labour.
- Your labour slows down.
- You request it during labour.

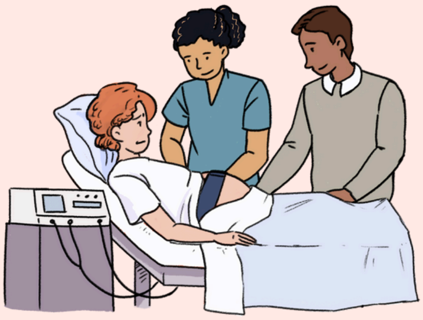

Emergency caesarean birth

This is when a caesarean is being advised because you or your baby's life is at risk. Please see the emergency caesarean core information set.

What are the other options for the birth of your baby?

Before labour, other options for the birth of your baby may include spontaneous or induced birth. You may find the Core Information Sets for **vaginal birth** or **induction of labour** helpful.

If you are in labour, continuing with labour or an instrumental birth with forceps or ventouse may be an option too.

You do not have to agree to a caesarean birth even if it is recommended.

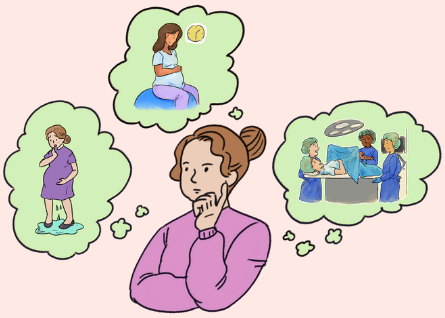

FUNDED BY NIHR | National Institute for Health and Care Research

UNIVERSITY OF LIVERPOOL

NHS Liverpool Women's NHS Foundation Trust

What if things CHANGE?

Birth Options

Pregnancy and birth are unpredictable.

It is useful to think about your birth preferences but you can change your decision at any time.

You may want to keep an open mind, or go-with-the-flow.

If a caesarean is planned but labour begins on its own, you should contact your maternity unit.

What are the benefits of caesarean birth?

The benefits of **planned caesarean birth** will depend on your circumstances, concerns, priorities and plans for future pregnancies.

- You may feel more in control.
- Compared to planned vaginal birth, there is less chance of:
    - Instrumental vaginal or emergency caesarean birth.
    - Vaginal tears.
    - Urine/bowel incontinence.
    - Pelvic organ prolapse requiring treatment in hospital.
    - Your baby's shoulders becoming stuck during birth (shoulder dystocia).
    - Your baby developing an infection during labour.

Please see table "comparison of planned caesarean and vaginal birth" for more information.

- There are contraceptive options during the operation e.g. fitting a coil or tube tying/removal (sterilisation).

Unplanned caesarean birth is usually offered for the health of you and/or your baby, or because you request one.

Emergency caesarean birth is advised because of a risk to the life of you or your baby.
Your doctors will discuss the benefits at the time.

FUNDED BY NIHR | National Institute for Health and Care Research

University of Liverpool

NHS Liverpool Women's NHS Foundation Trust

Risks at time of operation for mother

Birth Options

Bleeding

Losing more than 500mls of blood during caesarean birth is very common:
31 in 100 women/birthing people

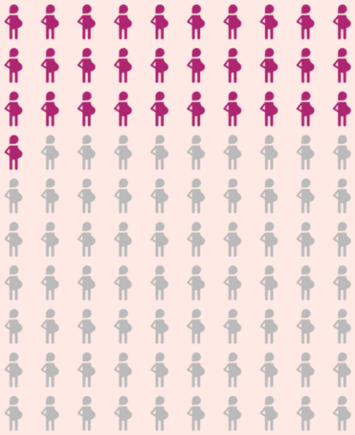

Needing a blood transfusion is common:
3 in 100 women/birthing people

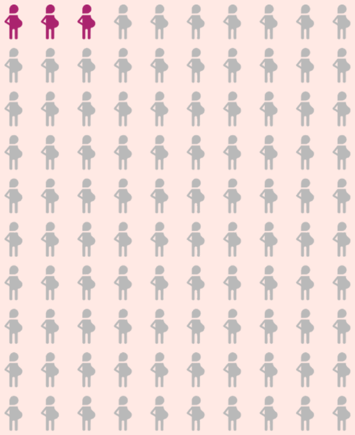

Emergency removal of the womb (hysterectomy) in order to stop bleeding and save your life is uncommon: **about 2 in 1000 women/birthing people.**

Chance of injury to nearby organs

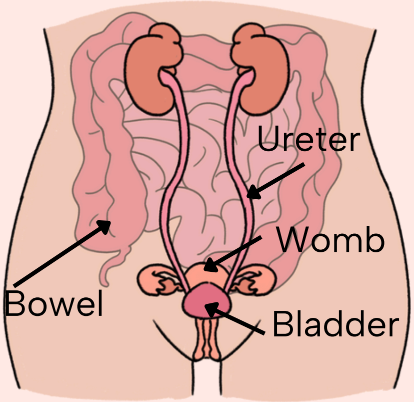

Injury to your bladder, the tubes connecting your kidney to your bladder (ureters) or bowel is uncommon:
1 in 1000 women/birthing people

Death of mother

Maternal death is rare: **25 in 100,000 women/birthing people**

There are also risks with anaesthetics, which are discussed in the *anaesthetic options* section.

FUNDED BY NIHR | National Institute for Health and Care Research
UNIVERSITY OF LIVERPOOL
NHS Liverpool Women's NHS Foundation Trust

Risk at time of operation for baby

Birth Options

Every type of birth comes with some risks for the baby, for caesarean this includes:

A scratch/cut your baby (this usually heals well)

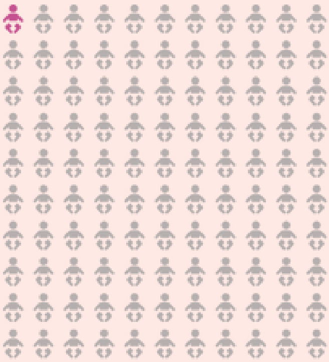

1–2 in 100 babies

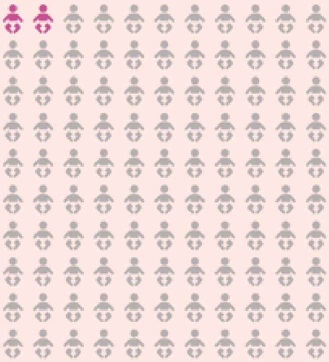

Difficulty delivering your baby

It may be difficult to deliver your baby. This happens in **10 in 100** unplanned/emergency caesarean births, but also happens in planned caesareans. Extra things like forceps may be needed during the operation.

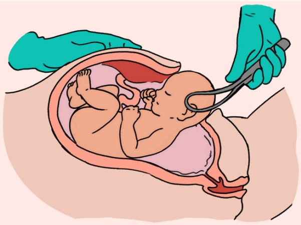

Breathing Difficulties

Sometimes when babies are born they need some help to move from receiving the oxygen from your placenta to breathing on their own:

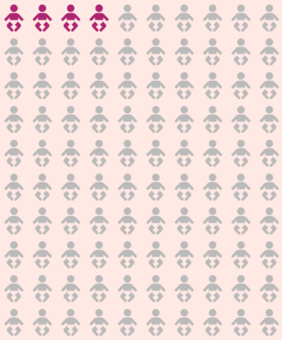

4 in 100 babies need extra help with breathing when they're born

Severe injury to the baby during birth is uncommon

1 in 1000 caesarean births

Brain injury

Brain injury due to lack of oxygen during birth (hypoxic ischaemic encephalopathy) is very rare.

FUNDED BY NIHR | National Institute for Health and Care Research

UNIVERSITY OF LIVERPOOL

NHS Liverpool Women's NHS Foundation Trust

Risks following the operation

Birth Options

Short-term risks for mother after birth

Going back to theatre

Sometimes because of bleeding, infections or other problems.
Another procedure may be needed.

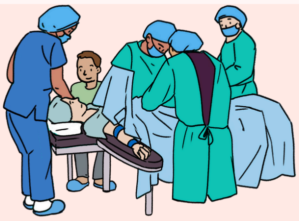

Admission to critical care unit

Admission to critical care is uncommon: **3 in 1000** women/birthing people.

Wound Infection

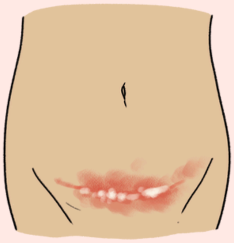

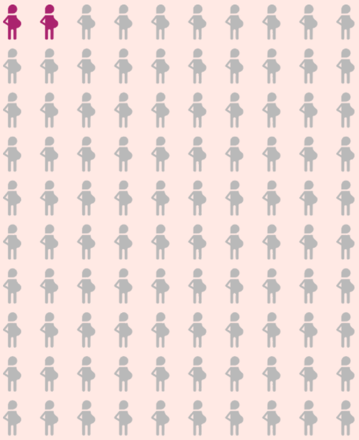

Wound infection is common:
2–7 in 100 women/birthing people.

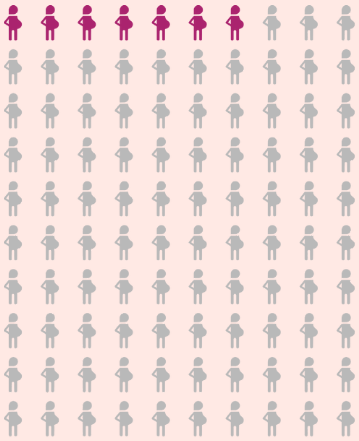

Mental health

Psychological complications such as postnatal depression or post-traumatic stress disorder (PTSD) are common:
3 in 100
women/birthing people

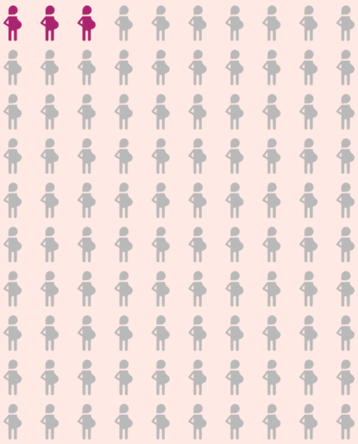

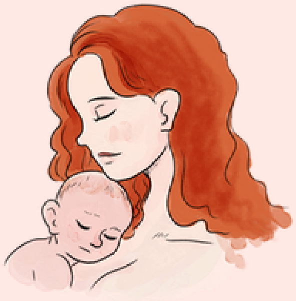

FUNDED BY NIHR | National Institute for Health and Care Research

UNIVERSITY OF LIVERPOOL

NHS Liverpool Women's NHS Foundation Trust

Risks Following the operation

Birth Options

Developing blood clots in legs and lungs

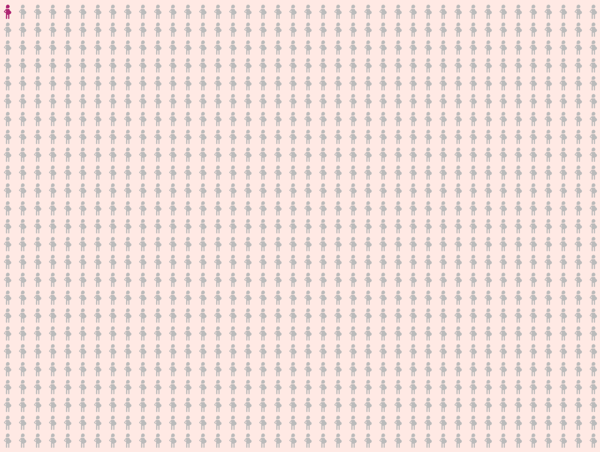

1-2 in 1000 women/birthing people

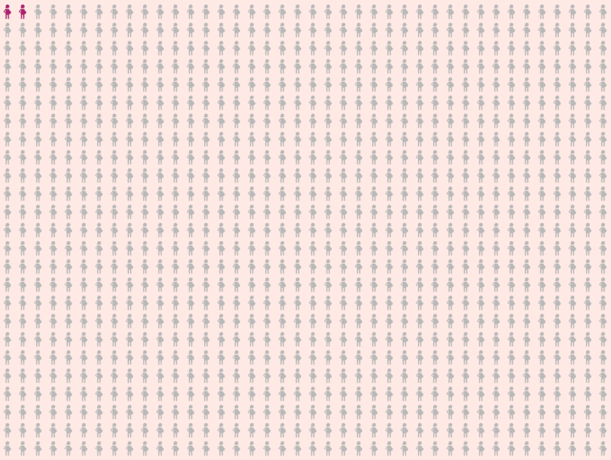

Long-term risks to mother after birth

Incontinence (leaking urine or faeces/wind)

Leaking urine (wee) more than a year after birth is common:

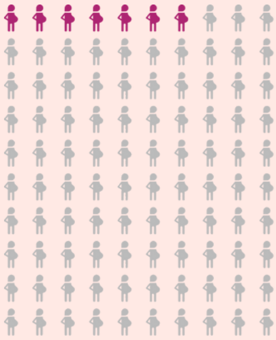

7-20 in 100 women/birthing people.

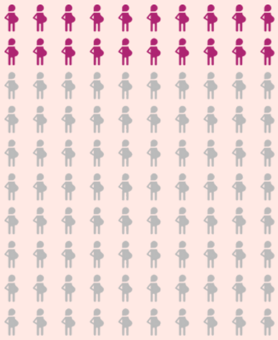

Leaking faeces (poo) or flatus (wind) more than a year after birth is common:

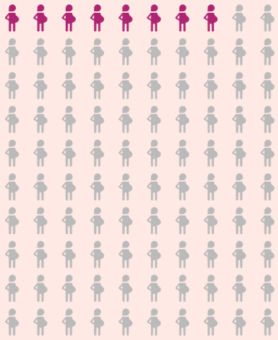

8 in 100 women/birthing people.

Prolapse

Pelvic organ prolapse (bulging bladder, womb or back passage (rectum)) requiring hospital treatment after a year is uncommon: **2 in 1000** women/birthing people

FUNDED BY NIHR National Institute for Health and Care Research
UNIVERSITY OF LIVERPOOL
NHS Liverpool Women's NHS Foundation Trust

Risks following the operation

Birth Options

Short-term risks for baby after birth

Admission to Neonatal Care Unit

Sometimes when baby's are born they will need to go to the neonatal unit for specialist care. This could be for breathing difficulties, infection, low blood sugars or other reasons.

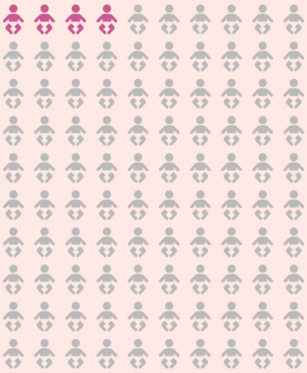

4-12 in 100

babies are admitted to the neonatal care unit after a caesarean birth.

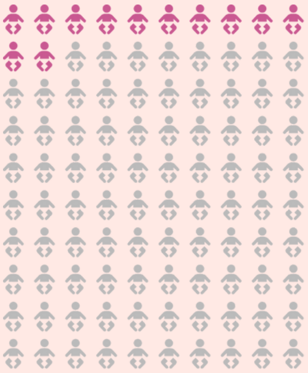

Death of baby (Up to 28 days after birth)

Neonatal death is rare: **58 in 100,000 babies.**

Long-term risks to baby after birth

For women/birthing people that have a caesarean birth, **18 in 1000** children will develop childhood asthma.

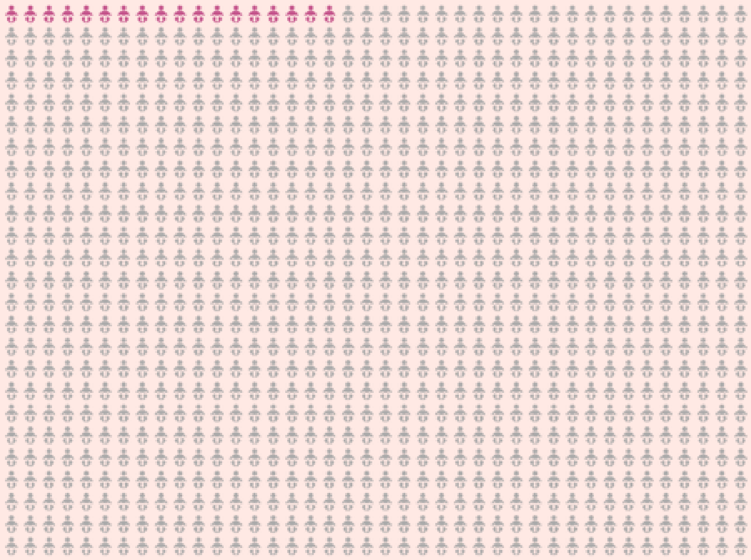

FUNDED BY NIHR | National Institute for Health and Care Research

UNIVERSITY OF LIVERPOOL

NHS Liverpool Women's NHS Foundation Trust

Anaesthetic options

Birth Options

There are two options for anaesthesia during a caesarean birth.
Regional and General Anaesthesia

Regional anaesthesia

Involves an **injection into your back** so the lower half of your body is numb and you are awake for your birth.

Types of regional anaesthesia and procedure:

Spinal anaesthesia: The most common method for caesarean births. An anaesthetic is injected into your back.

Epidural anaesthesia: A thin tube is placed near the nerves in your back to deliver a local anaesthetic. It is often used during labour but can be used for a caesarean.

Benefits of regional anaesthesia:

- Safer for you and your baby.
- You can share your birth experience with your partner.
- Less sickness and vomiting.
- Your baby is usually more alert at birth.

Potential side effects:

- Low blood pressure, dizziness, sickness/vomiting and shivering.
- Severe headaches are uncommon, about **1 in 200** women.
- Permanent nerve damage is very rare, approximately **1 in 50,000**.

General anaesthesia

You will be asleep for your birth

It is used for:

- Certain medical problems (e.g. bleeding conditions) preventing regional anaesthesia.
- If you are not comfortable with regional anaesthesia (spinal/epidural).
- Emergency caesarean births where the team feel a general anaesthetic will be quicker than regional anaesthesia.

Potential complications of general anaesthesia:

- Common problems are sickness, sore throat, shivering, and itching. These are short lived.
- Uncommonly women have problems with breathing or damage to lips/teeth
- Rarely, there are severe allergic reactions.
- Brain damage or death is very rare.

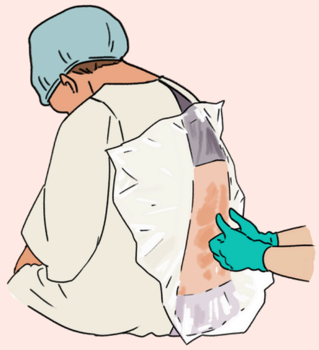

Regional anaesthesia

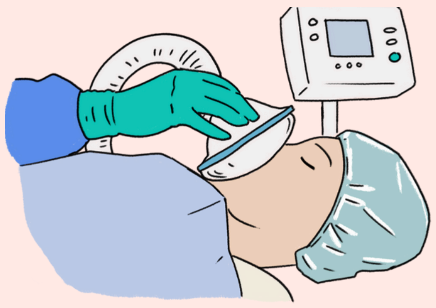

General anaesthesia

FUNDED BY NIHR National Institute for Health and Care Research
UNIVERSITY OF LIVERPOOL
NHS Liverpool Women's NHS Foundation Trust

Comparison of caesarean and vaginal birth

= Higher chance

Birth Options

More likely with caesarean birth		
**Outcomes**	**Caesarean birth**	**Vaginal birth**
**Hospital stay (average)**	4 days	2.5 days
**Removal of your uterus (hysterectomy)**	About **2 in 1,000** women/birthing people	About **1 in 1,000** women/birthing people
**Uterine rupture in future pregnancy***	About **200 in 100,000** women/birthing people. Chance increases with more caesarean births.	About **7 in 100,000** women/birthing people
**Abnormally adherent/invasive placenta in future pregnancy (placenta accreta spectrum)***	About **10 in 10,000** **women/birthing people**	About **3 in 10,000** women/birthing people
**Maternal death (within 6 weeks of childbirth)**	About **25 in 100,000** women/birthing people	About **4 in 100,000** women/birthing people
**Neonatal death (within 28 days of birth)**	**58 in 100,000** babies	**30 in 100,000** babies
**Childhood Asthma**	**18 in 1,000** babies	**15 in 1,000** babies

Less likely with caesarean birth		
**Outcomes**	**Caesarean birth**	**Vaginal birth**
**Perineal/abdominal pain (1=no pain 10=most severe pain)**	Pain scores: **1** – during birth **4.5** – 3 days after birth	Pain scores: **7.3** – during birth **5.2** – 3 days after birth
**Urinary incontinence (>1 year after birth)**	About **7 – 20 in 100** women/birthing people	About **49 in 100** women/birthing people
**Faecal (poo) incontinence (>1 year after birth)**	About **8 in 100** women/birthing people	About **15 in 100** women/birthing people
**Third or fourth-degree perineal tears**	**0 in 10,000** women/birthing people	About **56 in 10,000** women/birthing people

Likely to be similar for caesarean or vaginal birth

Blood clots in legs or lungs.
Severe blood loss (more than 1500mls).
Postnatal depression.
Admission to neonatal unit.
Your baby developing an infection.
Persistent verbal delay in baby/child.
Infant death (up to one year).

Conflicting or limited evidence

Admission to critical care.
Stillbirth in next pregnancy.
Lung problems in baby/child.
Cerebral palsy.
Childhood obesity.
Autism in baby/child.
Type 1 diabetes in baby/child.

FUNDED BY NIHR | National Institute for Health and Care Research
UNIVERSITY OF LIVERPOOL
NHS Liverpool Women's NHS Foundation Trust

Preparing for your planned caesarean birth

Birth Options

Consent for your caesarean birth

This may take place in person or over the phone.
This is a good opportunity to ask questions you may have.
You will usually be asked to sign a consent form.

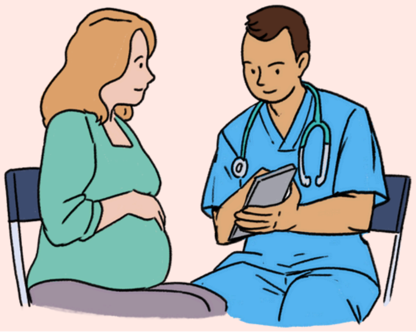

Pre-operation assessment

This may be an appointment or happen at the time of booking your operation, depending on the hospital policy.

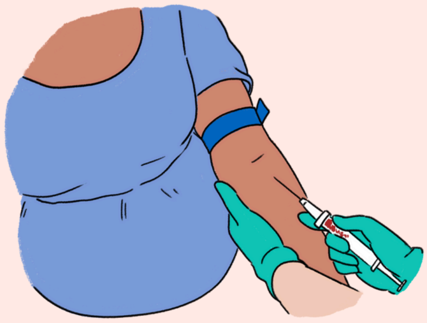

We will usually:

- Measure your blood pressure.
- Take some blood tests.
- Check your general health and pregnancy.
- You may be given antacid tablet to take the night before your caesarean.

When to stop eating and drinking before your operation

You will be given a time to stop eating and drinking before your operation because having food in your stomach carries a small risk of it going into your lungs and causing an infection if a general anaesthetic is needed.
You may be given a sugary drink to have before your operation.

On the day

You will usually be seen by the obstetric and anaesthetic doctors to check your consent and anaesthetic.
You may be given some antacid medication to take.
You will be asked to change into a surgical gown, given some tight socks (TEDs) and perhaps a cap.
You birth partner may be asked to change into a theatre outfit.

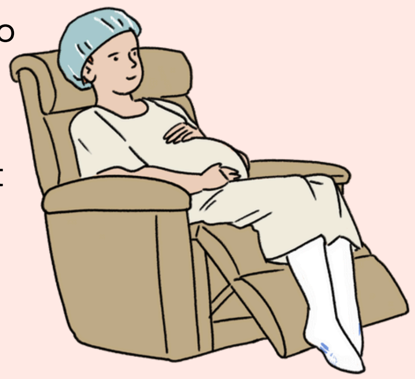

FUNDED BY NIHR | National Institute for Health and Care Research
UNIVERSITY OF LIVERPOOL
NHS Liverpool Women's NHS Foundation Trust

What to expect during a caesarean birth

Typically, a caesarean birth takes about 60 minutes, but it varies according to the complexity of the operation

	1. Consent	2. Blood taken	3. Getting ready for surgery	4. Before the operation starts	5. Surgical procedure	6. Managing bleeding	7. Immediately after surgery
	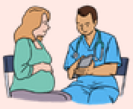	To prepare for a blood transfusion should you need it, blood may be taken. 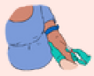	You will need to wear a surgical gown and semi-tight socks to reduce the risk of blood clots. One support person can be in the room with you if you have an epidural or spinal, but not a general anaesthetic. 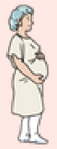 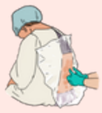 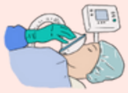	To reduce the risk of infection antibiotics will be given and your vagina may be cleaned.<br><br>The anaesthetist will ask if you can feel a cold sensation on your abdomen.<br><br>Medical staff will introduce themselves and do a safety checklist.<br><br>If you don't already have a urinary catheter, one will be inserted to protect your bladder.<br><br>Continuous monitoring of the baby may continue until the start of the operation.<br><br>Your abdomen will be cleaned and draped.	The cut on your abdomen is usually made just below the bikini line, about 10 cm long.<br><br>Your baby will be delivered through a cut in the womb.<br><br>Where possible, cord clamping will be delayed (optimised). Skin-to-skin contact and early breastfeeding will be encouraged as long as you and your baby are stable.<br><br>A waterproof dressing will cover the incision, and vaginal cleaning may be performed to remove blood/clots. 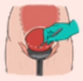	Bleeding is a very common complication of caesareans. Medication is given to keep the uterus contracted and help prevent excessive bleeding.<br><br>Surgical measures may be taken if bleeding continues, such as inserting a balloon in the uterus.<br><br>In rare cases, a hysterectomy may be necessary as a life-saving measure. 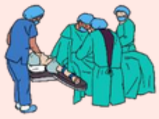	A checklist will be performed before transferring you to a recovery area.<br><br>You will remain in recovery until it is safe to move to your room.

Planned Caesarean birth

1. Antenatal appointment
2. Pre-Op Appointment including bloods
3. Prepare for surgery
4. Before the operation starts
5. Surgical Procedure
6. Managing Bleeding
7. Post - surgery

Unplanned Caesarean

1. Consent
2. Take bloods
3. Prepare for surgery
4. Before the operation starts
5. Surgical Procedure
6. Managing Bleeding
7. Post-surgery

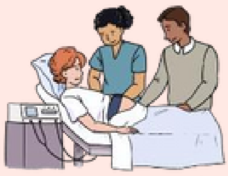

Emergency Caesarean Birth

1. Consent
2. Take bloods
3. Prepare for surgery
4. Before the operation starts
5. Surgical Procedure
6. Managing Bleeding
7. Post-surgery

FUNDED BY NIHR | National Institute for Health and Care Research

UNIVERSITY OF LIVERPOOL

NHS Liverpool Women's NHS Foundation Trust

Recovering after a caesarean birth

Birth Options

Bowels

It can take some time for your bowels to get back into a normal pattern, but this usually happens by about 1 week after birth. Some women will experience painful trapped wind and constipation, which you can take medication for.

Vaginal bleeding

- Vaginal bleeding after caesarean is normal; use period pads (not tampons) to avoid infection.
- You can expect bleeding as heavy as a period which will slowly tail off until it stops around two weeks.

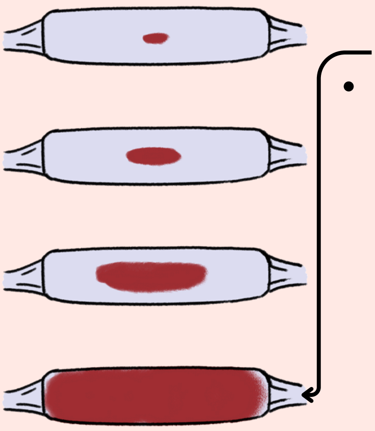

- If you are **soaking through pads** please see your midwife.
- If your bleeding slows down and then becomes heavier again speak to your midwife or doctor. It may be a sign of an infection.

Managing pain

Pain management both whilst in hospital and at home

You have had a big operation. It is important to take painkillers regularly to keep the pain under control. You will usually be offered paracetamol, dihydrocodeine and an anti-inflammatory (ibuprofen/naproxen/diclofenac).

In the hospital you may also be offered other drugs if needed, for example, morphine.

Caesarean scar pain in the short and long term

The scar (usually horizontal below the bikini line) will fade with time. Discomfort may last several days to weeks; but can continue for several months.

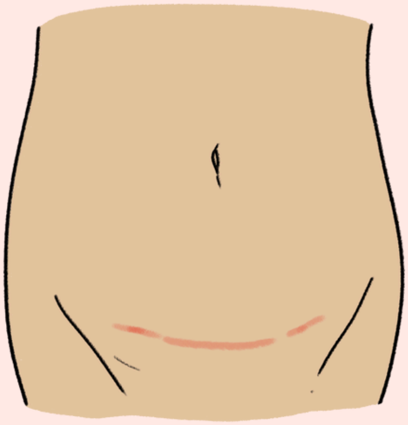

FUNDED BY NIHR | National Institute for Health and Care Research
UNIVERSITY OF LIVERPOOL
NHS Liverpool Women's NHS Foundation Trust

What to expect following a caesarean birth

Birth Options

Pain

You will experience pain after your caesarean. This will improve over time.
Each woman is different but pain should begin to get better by 2 weeks, although your cut is likely to remain tender for longer than this.

Feeding your baby

Both breastfeeding and formula feeding can be supported around the time of birth.
In theatre you or your birth partner may wish to have skin to skin.

Food and Drink

You will be able to eat and drink soon after birth once you feel up to it.

Moving to the ward

You will be moved from the recovery room to the ward within a couple of hours of your baby being born depending on how you are.

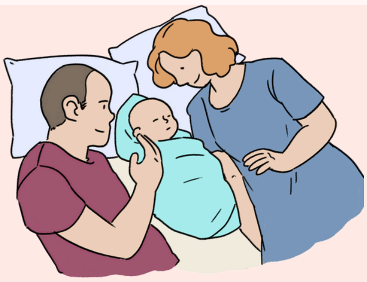

Walking

You will not be able to walk, shower, or have your catheter removed until you can feel and move your legs properly.

Catheter removal

Typically, bladder function returns in a few hours. Your catheter will usually be removed after 12 hours, once you are able to walk around. There may be some pain/discomfort when weeing (or when bladder is full) as you recover from the caesarean. Sometimes the bladder needs to rest more and you may need another catheter. You may need to go home with this for a few days.

FUNDED BY NIHR | National Institute for Health and Care Research
UNIVERSITY OF LIVERPOOL
NHS Liverpool Women's NHS Foundation Trust

What to expect following a caesarean birth

Birth Options

Wound Dressing

You will be advised to leave your dressing on for at least 24 hours.

Length of time in hospital

Many women will go home approximately 24 hours after a planned caesarean however some will stay longer.

Prepare for your total hospital stay to be about 4 days.

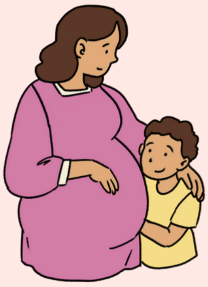

Exercise and heavy lifting

We would advise no heavy lifting for 4-6 weeks.

Your body needs time to heal. You should be guided by your body in terms of exercise. It may be best to wait 6 weeks to resume high-impact exercise.

Sexual Activity

You should be guided by your body in terms of when you are ready to resume sexual activity.

Driving

You will be advised not to drive until you can do an emergency stop. This is often 4-6 weeks, but may be sooner. You are advised to contact your insurance company.

Blood thinning medication to reduce the risk of blood clots in legs and lungs

You will have an assessment of your risk of clots in your legs and lungs (deep vein thrombosis/pulmonary embolism) at the time of your caesarean.

Many women will be offered blood thinners as injections to reduce this risk. These can be taken at home for 10 days – 6 weeks.

FUNDED BY NIHR | National Institute for Health and Care Research
UNIVERSITY OF LIVERPOOL
NHS Liverpool Women's NHS Foundation Trust

Future pregnancies following a caesarean birth

The effects of caesarean birth on future pregnancies

Low-lying placenta (placenta praevia)

Where the placenta blocks the exit of the womb.

9 in 1000 women/birthing people

This increases with the number of caesarean births you have.

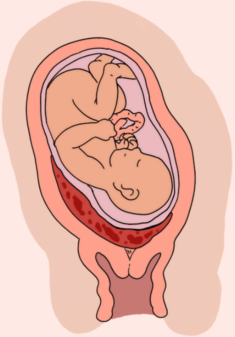

Invasive placenta (placenta accreta)

The placenta invades the wall of the womb. *This increases with the number of caesarean births you have.*

1 in 1000

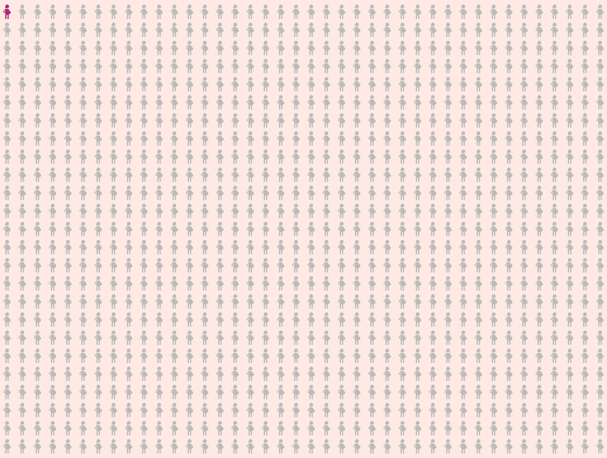

Stillbirth

Stillbirth after 39 weeks is uncommon, but may be increased in pregnancies after caesarean birth.

The *evidence is unclear.*

Womb (uterine) rupture

A hole forms in the womb.

2 in 1000 women/birthing people

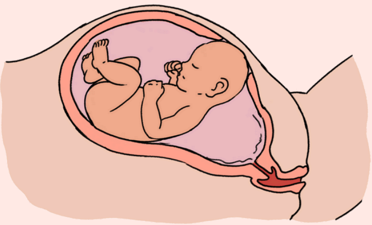

Future birth options

Choosing between a repeat caesarean birth or a vaginal birth after a caesarean birth (VBAC) may be an option. This will depend on the reason for your previous caesarean birth and your next pregnancy.

FUNDED BY NIHR | National Institute for Health and Care Research

University of Liverpool

NHS Liverpool Women's NHS Foundation Trust

WHERE WE GOT THIS INFORMATION FROM

Birth Options

"How common are caesareans?":

- NHS Maternity Statistics, England, 2023-24. last accessed 10/03/25. Available from: https://digital.nhs.uk/data-and-information/publications/statistical/nhs-maternity-statistics/2023-24/births
- Jardine J, Blotkamp A, Gurol-Urganci I, Knight H, Harris T, Hawdon J, van der Meulen J, Walker K, Pasupathy D. Risk of complicated birth at term in nulliparous and multiparous women using routinely collected maternity data in England: cohort study. BMJ. 2020 Oct 1;371:m3377. doi: 10.1136/bmj.m3377

Why a caesarean birth may be offered

- Caesarean birth. London: National Institute for Health and Care Excellence (NICE); 2024 Jan 30. PMID: 33877751. Available from: https://www.nice.org.uk/guidance/ng192/chapter/Recommendations.
- Twin and triplet pregnancy. London: National Institute for Health and Care Excellence (NICE); 2024 Apr 9. PMID: 31513365. Available from: https://www.nice.org.uk/guidance/NG137.
- Birth after Previous Caesarean Birth (Green-top Guideline No. 45) | RCOG Accessed: 10/03/2025. Available from: https://www.rcog.org.uk/guidance/browse-all-guidance/green-top-guidelines/birth-after-previous-caesarean-birth-green-top-guideline-no-45/.

What are the other options for the birth of your baby?

- Intrapartum care. London: National Institute for Health and Care Excellence (NICE); 2023 Sep 29. PMID: 37871143. Available from: https://www.nice.org.uk/guidance/ng235
- Assisted Vaginal Birth - Murphy - 2020 - BJOG: An International Journal of Obstetrics & Gynaecology - Wiley Online Library. Accessed: 10/03/2025. Available from: https://www.rcog.org.uk/guidance/browse-all-guidance/green-top-guidelines/assisted-vaginal-birth-green-top-guideline-no-26/
- Antenatal care. London: National Institute for Health and Care Excellence (NICE); 2021 Aug 19. PMID: 34524750. Available from: https://www.nice.org.uk/guidance/ng201/chapter/Recommendations

What if things change?

- Antenatal care. London: National Institute for Health and Care Excellence (NICE); 2021 Aug 19. PMID: 34524750. Available from: https://www.nice.org.uk/guidance/ng201/chapter/Recommendations
- Kingdon C, Neilson J, Singleton V, Gyte G, Hart A, Gabbay M, et al. Choice and birth method: mixed-method study of caesarean delivery for maternal request. Bjog. 2009;116(7):886-95.
- Divall B, Spiby H, Nolan M, Slade P. Plans, preferences or going with the flow: An online exploration of women's views and experiences of birth plans. Midwifery. 2017 Nov;54:29-34. doi: 10.1016/j.midw.2017.07.020.

Benefits of caesarean birth

- Caesarean birth. London: National Institute for Health and Care Excellence (NICE); 2024 Jan 30. PMID: 33877751. Available from: https://www.nice.org.uk/guidance/ng192/chapter/Recommendations.
- Chen I, Opiyo N, Tavender E, Mortazhejri S, Rader T, Petkovic J, Yogasingam S, Taljaard M, Agarwal S, Laopaiboon M, Wasiak J,Khunpradit S, Lumbiganon P, Gruen RL, Betran AP.Non-clinical interventions for reducing unnecessary caesarean section.Cochrane Database of Systematic Reviews 2018, Issue 9. Art. No.: CD005528.DOI: 10.1002/14651858.CD005528.pub3.
- Adewale V, Varotsis D, Iyer N, Di Mascio D, Dupont A, Abramowitz L, Steer PJ, Gimovsky M, Berghella V. Planned cesarean delivery vs planned vaginal delivery: a systematic review and meta-analysis of randomized controlled trials. Am J Obstet Gynecol MFM. 2023 Dec;5(12):101186. doi: 10.1016/j.ajogmf.2023.101186.
- Coates D, Thirukumar P, Henry A. Women's experiences and satisfaction with having a cesarean birth: An integrative review. Birth. 2020 Jun;47(2):169-182. doi: 10.1111/birt.12478

Risks at time of operation for mother

- NHS Maternity Statistics, England, 2023-24. last accessed 10/03/25. Available from: https://digital.nhs.uk/data-and-information/publications/statistical/nhs-maternity-statistics/2023-24/births
- Khan KS, Moore PAS, Wilson MJ, Hooper R, Allard S, Wrench I, Beresford L, Roberts TE, McLoughlin C, Geoghegan J, Daniels JP, Catling S, Clark VA, Ayuk P, Robson S, Gao-Smith F, Hogg M, Lanz D, Dodds J; SALVO study group. Cell salvage and donor blood transfusion during cesarean section: A pragmatic, multicentre randomised controlled trial (SALVO). PLoS Med. 2017 Dec 19;14(12):e1002471. doi: 10.1371/journal.pmed.1002471
- Royal College of Obstetricians and Gynaecologists. Planned Caesarean Birth: Consent Advice No. 14 August 2022 Minor Update November 2024
- Larsson C, Djuvfelt E, Lindam A, Tunón K, Nordin P. Surgical complications after caesarean section: A population-based cohort study. PLoS One. 2021 Oct 5;16(10):e0258222. doi: 10.1371/journal.pone.0258222

Risks following the operation

- Royal College of Obstetricians and Gynaecologists. Planned Caesarean Birth: Consent Advice No. 14 August 2022 Minor Update November 2024.
- Larsson C, Källen K, Andolf E. Cesarean section and risk of pelvic organ prolapse: a nested case-control study. Am J Obstet Gynecol. 2009 Mar;200(3):243.e1-4. doi: 10.1016/j.ajog.2008.11.028.
- Rowlands, I.J., Redshaw, M. Mode of birth and women's psychological and physical wellbeing in the postnatal period. BMC Pregnancy Childbirth 12, 138 (2012). https://doi.org/10.1186/1471-2393-12-138.
- Reducing the Risk of Thrombosis and Embolism during Pregnancy and the Puerperium (Green-top Guideline No. 37a). Accessed 10/03/2025. Available from: https://www.rcog.org.uk/guidance/browse-all-guidance/green-top-guidelines/reducing-the-risk-of-thrombosis-and-embolism-during-pregnancy-and-the-puerperium-green-top-guideline-no-37a/.
- Herstad L, Klungsøyr K, Skjærven R, Tanbo T, Forsén L, Åbyholm T, Vangen S. Elective cesarean section or not? Maternal age and risk of adverse outcomes at term: a population-based registry study of low-risk primiparous women. BMC Pregnancy Childbirth. 2016 Aug 17;16:230. DOI: 10.1186/s12884-016-1028-3.

Comparison of planned caesarean and vaginal birth

- Caesarean birth. London: National Institute for Health and Care Excellence (NICE); 2024 Jan 30. PMID: 33877751. Available from: https://www.nice.org.uk/guidance/ng192/chapter/Recommendations.

Risks at time of operation for baby

- Royal College of Obstetricians and Gynaecologists. Planned Caesarean Birth: Consent Advice No. 14 August 2022 Minor Update November 2024.
- Cornthwaite K, Draycott T, Bahl R, Hotton E, Winter C, Lenguerrand E. Impacted fetal head: A retrospective cohort study of emergency caesarean section. Eur J Obstet Gynecol Reprod Biol. 2021 Jun;261:85-91. doi: 10.1016/j.ejogrb.2021.04.021. Epub 2021 Apr 21.
- Hansen AK, Wisborg K, Uldbjerg N, Henriksen TB. Risk of respiratory morbidity in term infants delivered by elective caesarean section: cohort study. BMJ. 2008 Jan 12;336(7635):85-7. doi: 10.1136/bmj.39405.539282.BE. Epub 2007 Dec 11
- Liston FA, Allen VM, O'Connell CM, Jangaard KA. Neonatal outcomes with caesarean delivery at term. Arch Dis Child Fetal Neonatal Ed. 2008 May;93(3):F176-82. doi: 10.1136/adc.2006.112565. Epub 2007 Oct 17
- Herstad, L., Klungsøyr, K., Skjærven, R. et al. Elective cesarean section or not? Maternal age and risk of adverse outcomes at term: a population-based registry study of low-risk primiparous women. BMC Pregnancy Childbirth 16, 230 (2016). https://doi.org/10.1186/s12884-016-1028-3
- MacDorman MF, Declercq E, Menacker F, Malloy MH. Neonatal mortality for primary cesarean and vaginal births to low-risk women: application of an "intention-to-treat" model. Birth. 2008 Mar;35(1):3-8. doi: 10.1111/j.1523-536X.2007.00205.x.

Anaesthetic options

- Holdcroft A, Gibberd FB, Hargrove RL, Hawkins DF, Dellaportas CI. Neurological complications associated with pregnancy. British Journal of Anaesthesia 1995 – chapter 75, pages 522–526.
- Jenkins K, Baker AB. Consent and anaesthetic risk. Anaesthesia 2003 – chapter 58, pages 962–984.
- Jenkins JG, Khan MM. Anaesthesia for Caesarean section: a survey in a UK region from 1992 to 2002. Anaesthesia 2003 – chapter 58, pages 1114-1118.
- Jenkins JG. Some immediate serious complications of obstetric epidural analgesia and anaesthesia: a prospective study of 145,550 epidurals. International Journal of Obstetric Anesthesia 2005 – chapter 14, pages 37–42.
- Reynolds F. Infection a complication of neuraxial blockade. International Journal of Obstetric Anesthesia 2005 – chapter 14, pages 183-188.
- Ruppen W, Derry S, McQuay H, Moore RA. Incidence of epidural hematoma, infection, and neurologic injury in obstetric patients with epidural analgesia/ anesthesia. Anesthesiology 2006 – chapter 105, pages 394–399.
- Cook TM, Counsell D, Wildsmith JAW. Major complications of central neuraxial block: report on the third National Audit Project of the Royal College of Anaesthetists. British Journal of Anaesthesia 2009; 102: 179-190
- Pandit JJ, Cook TM. The 5th National Audit Project of the Royal College of Anaesthetists and The Association of Anaesthetists of Great Britain and Ireland. Accidental Awareness during General Anaesthesia in the United Kingdom and Ireland. September 2014

Preparing for your caesarean birth

- Caesarean birth. London: National Institute for Health and Care Excellence (NICE); 2024 Jan 30. PMID: 33877751. Available from: https://www.nice.org.uk/guidance/ng192/chapter/Recommendations.
- Royal College of Obstetricians and Gynaecologists. Planned Caesarean Birth: Consent Advice No. 14 August 2022 Minor Update November 2024.

What to expect during a caesarean birth

- Caesarean birth. London: National Institute for Health and Care Excellence (NICE); 2024 Jan 30. PMID: 33877751. Available from: https://www.nice.org.uk/guidance/ng192/chapter/Recommendations.
- Mavrides E, Allard S, Chandraharan E, Collins P, Green L, Hunt BJ, Riris S, Thomson AJ on behalfof the Royal College of Obstetricians and Gynaecologists. Prevention and management of postpartum haemorrhage.BJOG 2016 ; 124:e106–e149.
- Reducing the Risk of Thrombosis and Embolism during Pregnancy and the Puerperium (Green-top Guideline No. 37a). Accessed 10/03/2025. Available from: https://www.rcog.org.uk/guidance/browse-all-guidance/green-top-guidelines/reducing-the-risk-of-thrombosis-and-embolism-during-pregnancy-and-the-puerperium-green-top-guideline-no-37a/.

What to expect following a caesarean birth

- Caesarean birth. London: National Institute for Health and Care Excellence (NICE); 2024 Jan 30. PMID: 33877751. Available from: https://www.nice.org.uk/guidance/ng192/chapter/Recommendations.
- Postnatal Care. London: National Institute for Health and Care Excellence (NICE); 2021 April 20. Available from: https://www.nice.org.uk/guidance/ng194
- NHS Maternity Statistics, England, 2023-24. last accessed 10/03/25. Available from: https://digital.nhs.uk/data-and-information/publications/statistical/nhs-maternity-statistics/2023-24/births
- Reducing the Risk of Thrombosis and Embolism during Pregnancy and the Puerperium (Green-top Guideline No. 37a). Accessed 10/03/2025. Available from: https://www.rcog.org.uk/guidance/browse-all-guidance/green-top-guidelines/reducing-the-risk-of-thrombosis-and-embolism-during-pregnancy-and-the-puerperium-green-top-guideline-no-37a/.

Recovering after a caesarean birth

- Caesarean birth. London: National Institute for Health and Care Excellence (NICE); 2024 Jan 30. PMID: 33877751. Available from: https://www.nice.org.uk/guidance/ng192/chapter/Recommendations.
- Postnatal Care. London: National Institute for Health and Care Excellence (NICE); 2021 April 20. Available from: https://www.nice.org.uk/guidance/ng194

Future pregnancies following a caesarean birth

- Caesarean birth. London: National Institute for Health and Care Excellence (NICE); 2024 Jan 30. PMID: 33877751. Available from:https://gbr01.safelinks.protection.outlook.com/?url=https%3A%2F%2Fwww.nice.org.uk%2Fguidance%2Fng192%2Fchapter%2FRecommendations&data=05%7C02%7Cmahmoud.aljubeh%40lwh.nhs.uk%7C3843a0ca508a4072072e08dd692e5ca2%7C473870bd20104a57ba6ddb4e47cf3d8b%7C0%7C0%7C638782370042525968%7CUnknown%7CTWFpbGZsb3d8eyJFbXB0eU1hcGkiOnRydWUsIlYiOiIwLjAuMDAwMCIsIlAiOiJXaW4zMiIsIkFOIjoiTWFpbCIsIldUIjoyfQ%3D%3D%7C0%7C%7C%7C&sdata=VWIaTW8HtlIPd%2FOFzenlUxt1YECIr4RTIGij0bR%2FmXE%3D&reserved=0.
- Gurol-Urganci I, Cromwell DA, Edozien LC, Smith GC, Onwere C, Mahmood TA, Templeton A, van der Meulen JH. Risk of placenta previa in second birth after first birth cesarean section: a population-based study and meta-analysis. BMC Pregnancy Childbirth. 2011 Nov 21;11:95. doi: 10.1186/1471-2393-11-95.
- Royal College of Obstetricians and Gynaecologists. Planned Caesarean Birth: Consent Advice No. 14 August 2022 Minor Update November 2024.
- Birth after Previous Caesarean Birth (Green-top Guideline No. 45) | RCOG Accessed: 10/03/2025. Available from: https://www.rcog.org.uk/guidance/browse-all-guidance/green-top-guidelines/birth-after-previous-caesarean-birth-green-top-guideline-no-45/.

How we present information about risk

**Very common**	**1 in 1 to 1 in 10**
**Common**	**Less than 1 in 10 to 1 in 100**
**Uncommon**	**Less than 1 in 100 to 1 in 1000**
**Rare**	**Less than 1 in 1000 to 1 in 10,000**
**Very rare**	**Less than 1 in 10,000**

FUNDED BY NIHR | National Institute for Health and Care Research

UNIVERSITY OF LIVERPOOL

NHS Liverpool Women's NHS Foundation Trust
