# Supplementary material for: Informing Decision‐Making About Caesarean Birth: A Delphi Study to Develop a Core Information Set
Source: BJOG. 2025 Jul 8;132(13):2024–39. doi: 10.1111/1471-0528.18269 (PMC12592771; doi:10.1111/1471-0528.18269)
Supplement: Supplementary file 13 — Data S13. [file BJO-132-2024-s005.pdf]

Emergency Caesarean Birth Core Information Set

Birth Options

What is a core information set?

A core information set is the information everyone needs before making a decision about their care. They do not replace personalised discussions. The Birth Options core information sets have been made for families and healthcare professionals to use to provide information to support decisions about birth. Women, birthing people, partners, midwives and doctors have decided which information is most important.

This information is intended as a guide and uses evidence from national guidelines, national statistics and research studies. It includes some more general information that explains usual practice. It has the best available information at the time that it was made (2025).

This core information set is for emergency caesarean birth (birth within 30 minutes).

This is intended for use during an emergency to give you the information you need to know to make an informed decision quickly.

There is also a **planned/unplanned caesarean birth** core information set when there is time for discussion during antenatal care and a **postnatal** core information set which you may find useful to discuss before you leave the hospital.

Caesarean birth information sets do not include extensive information about induction of labour, spontaneous vaginal birth or instrumental vaginal birth. Other Core Information Sets are available for **induction of labour** and **vaginal birth.**

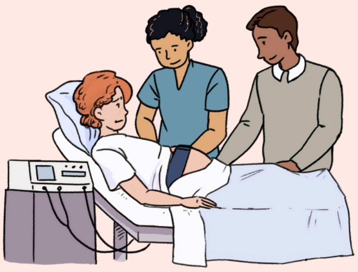

www.birthoptions.co.uk

FUNDED BY NIHR | National Institute for Health and Care Research

UNIVERSITY OF LIVERPOOL

NHS Liverpool Women's NHS Foundation Trust

Reason for emergency caesarean birth

A caesarean is being advised because of an immediate threat to the life of you or your baby, this is usually because:

Your baby's heart rate has dropped dangerously low

There are no signs of it getting/ staying higher. This means your baby is not getting enough oxygen.

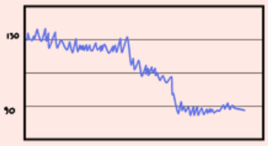

Cord prolapse

The umbilical cord has dropped from your womb into your vagina which could be stopping the blood supply to your baby.

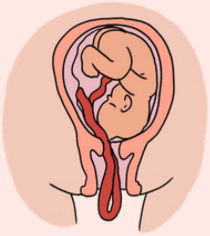

A problem with your placenta

You have significant bleeding because your placenta has separated before your baby is born or your placenta is covering the neck of your womb (cervix).

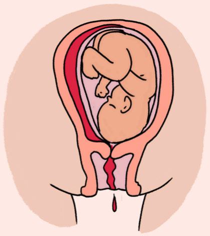

A tear in your womb (uterine rupture)

We suspect a tear in your womb which means your baby cannot get enough oxygen and you are at risk of serious bleeding.

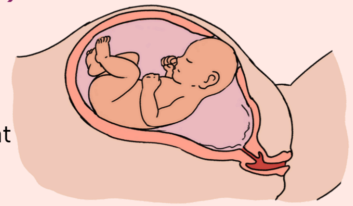

Risks at time of operation for baby

A baby doctor (neonatologist) will be in the room to assess your baby at birth. There is a chance of accidental injury to your baby e.g. cut to skin. Your baby may need help breathing. Your baby may need to go to the neonatal unit for specialist care.

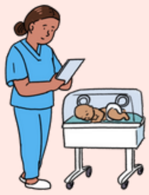

Risks at time of operation for mother

There is an increased chance of bleeding, infection, and injury to bladder/ bowel and blood vessels for you.

Emergency measures that may become necessary during the operation

If there are any known injuries to your bowel, bladder, or blood vessels these will be repaired during the surgery, we may need to take additional steps to deliver your baby e.g. Forceps/vaginal examination. If you experience excessive bleeding we need to stop it, if we cannot, we may need to perform a hysterectomy.

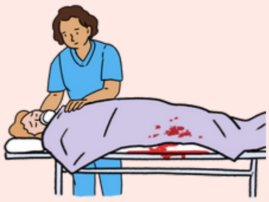

Anaesthetic options

- A spinal anaesthetic – a small needle in your back will be offered where possible.
- If you have an effective epidural in place it may be possible to use this.
- A general anaesthetic may be needed.

Your anaesthetist will advise.

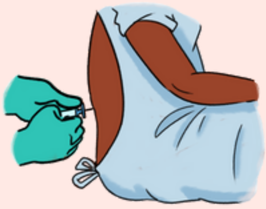

Epidural anaesthetic

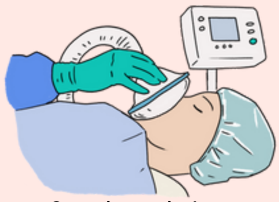

General anaesthetic

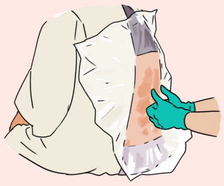

Spinal anaesthetic

FUNDED BY NIHR | National Institute for Health and Care Research

UNIVERSITY OF LIVERPOOL

NHS Liverpool Women's NHS Foundation Trust
