# Supplementary material for: Informing Decision‐Making About Caesarean Birth: A Delphi Study to Develop a Core Information Set
Source: BJOG. 2025 Jul 8;132(13):2024–39. doi: 10.1111/1471-0528.18269 (PMC12592771; doi:10.1111/1471-0528.18269)
Supplement: Supplementary file 14 — Data S14. [file BJO-132-2024-s009.pdf]

Postnatal Caesarean Birth Core Information Set

Birth Options

What is a core information set?

A core information set is the information everyone needs before making a decision about their care. They do not replace personalised discussions. The Birth Options core information sets have been made for families and healthcare professionals to use to provide information to support decisions about birth. Women, birthing people, partners, midwives and doctors have decided which information is most important.

This information is intended as a guide and uses evidence from national guidelines, national statistics and research studies. It includes some more general information that explains usual practice. It has the best available information at the time that it was made (2025).

This core information set is for after you've had an unplanned or emergency caesarean birth.

It may be useful to you as things may not have gone to plan.

This postnatal core information set may be used to inform conversations with your midwives or doctors before you leave hospital.

There is also an **antenatal planned/unplanned** caesarean birth core information set and a 5-point core information set for **emergency caesarean birth**.

Caesarean birth information sets do not include extensive information about induction of labour, spontaneous vaginal birth or instrumental vaginal birth. Other Core Information Sets are available for **induction of labour** and **vaginal birth.**

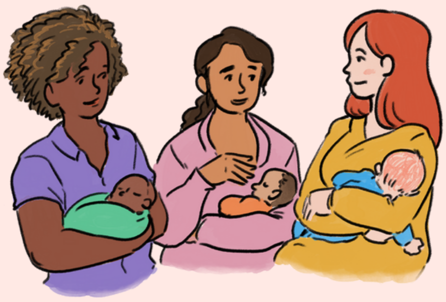

www.birthoptions.co.uk

FUNDED BY NIHR | National Institute for Health and Care Research

University of Liverpool

NHS Liverpool Women's NHS Foundation Trust

INFORMATION POINT	THINGS TO TALK TO YOUR MIDWIFE/DOCTOR ABOUT
Why caesarean birth was offered	• Reasons why caesarean was offered or needed – because of mother, baby or both?
How common caesarean birth is	• In England between 2023-2024 overall, **42%** of babies are born by caesarean.<br>• **44%** of women have a caesarean birth in their first pregnancy: **13%** are planned **31%** are unplanned or emergency.
Were there any other realistic options for you for the birth?	• Vaginal/instrumental/waiting longer/other
Were there any other realistic options for your baby for the birth?	• Vaginal/instrumental/waiting longer/other
How the caesarean birth went	• How the operation was performed.<br>• How long the operation took.<br>• How bleeding was managed.<br>• The use of a urinary catheter to protect your bladder.<br>• Were there any emergency measures necessary during the procedure?<br>• What was done to reduce the chance of infection?<br>• Where is the scar on my skin?<br>• Where is the scar on my womb?
Anaesthetic information	• Anaesthetic used (epidural/spinal/general anaesthetic).<br>• Common side effects I may experience now and in future.
Any complications at the time of the operation for you?	• How were these managed, if any?<br>• Any short or long term effects.
Any complications at the time of the operation for your baby?	• How were these managed, if any?<br>• Any short or long term effects.

FUNDED BY NIHR | National Institute for Health and Care Research
UNIVERSITY OF LIVERPOOL
NHS Liverpool Women's NHS Foundation Trust

Risks following the operation

Birth Options

Short-term risks for mother after birth

Going back to theatre

Sometimes because of bleeding, infections or other problems. Another procedure may be needed.

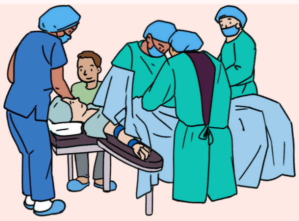

Admission to critical care unit

Admission to critical care is uncommon: **3 in 1000** women/birthing people.

Wound Infection

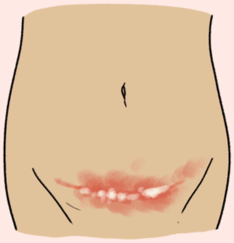

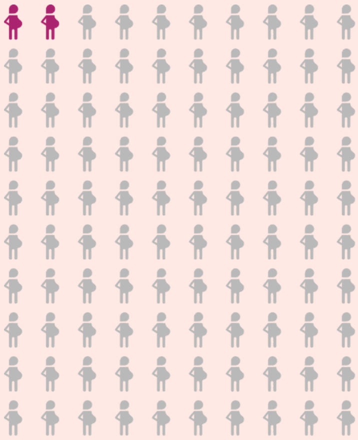

Wound infection is common:
2-7 in 100 women/birthing people.

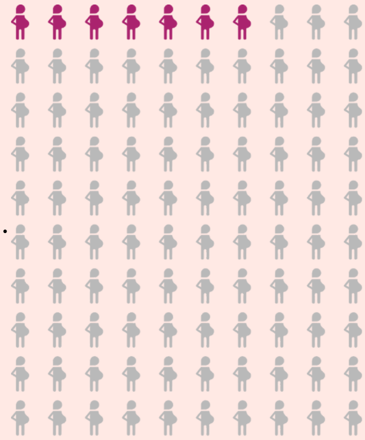

Mental health

Psychological complications such as postnatal depression or post-traumatic stress disorder (PTSD) are common:
3 in 100
women/birthing people

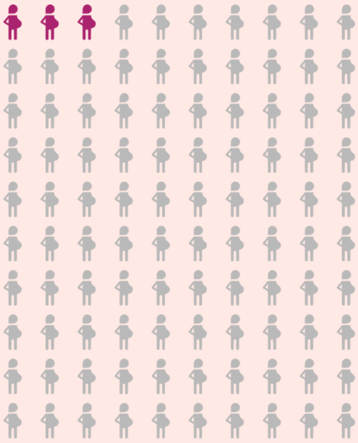

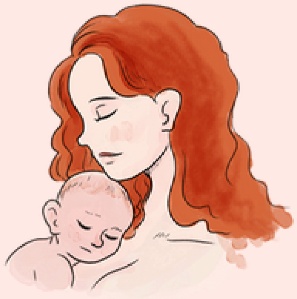

FUNDED BY NIHR | National Institute for Health and Care Research
UNIVERSITY OF LIVERPOOL
NHS Liverpool Women's NHS Foundation Trust

Risks Following the operation

Birth Options

Developing blood clots in legs and lungs

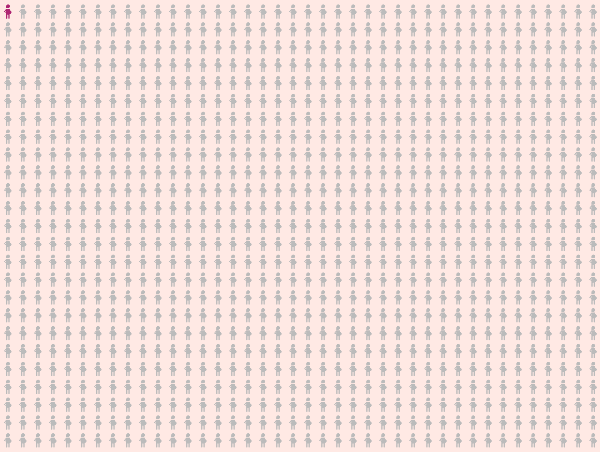

1-2 in 1000 women/birthing people

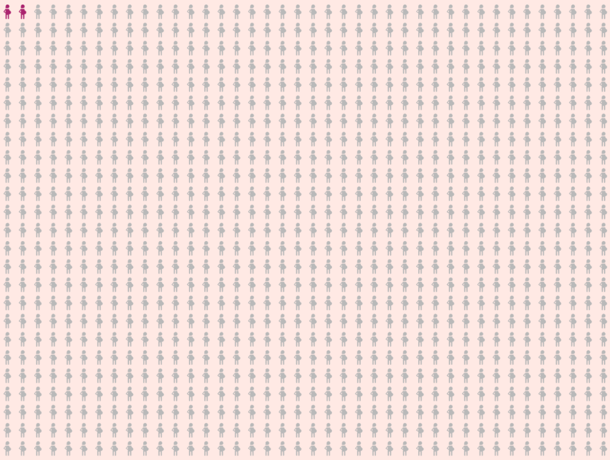

Long-term risks to mother after birth

Incontinence (leaking urine or faeces/wind)

Leaking urine (wee) more than a year after birth is common:

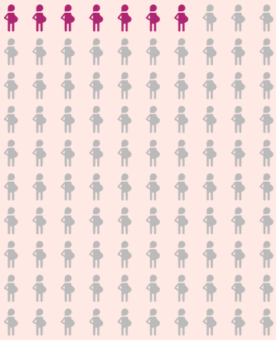

7-20 in 100 women/birthing people.

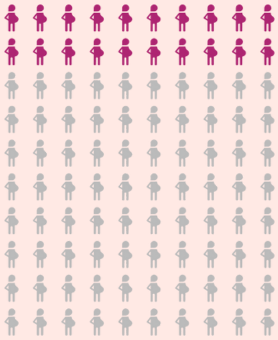

Leaking faeces (poo) or flatus (wind) more than a year after birth is common:

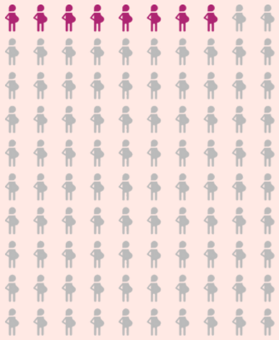

8 in 100 women/birthing people.

Prolapse

Pelvic organ prolapse (bulging bladder, womb or back passage (rectum)) requiring hospital treatment after a year is uncommon: **2 in 1000** women/birthing people

FUNDED BY NIHR | National Institute for Health and Care Research

UNIVERSITY OF LIVERPOOL

NHS Liverpool Women's NHS Foundation Trust

Risks following the operation

Birth Options

Short-term risks for baby after birth

Admission to Neonatal Care Unit

Sometimes when baby's are born they will need to go to the neonatal unit for specialist care. This could be for breathing difficulties, infection, low blood sugars or other reasons.

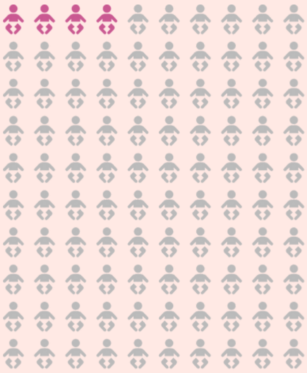

4-12 in 100

babies are admitted to the neonatal care unit after a caesarean birth.

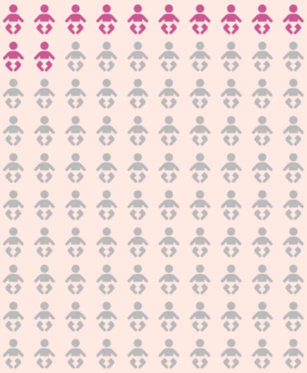

Death of baby (Up to 28 days after birth)

Neonatal death is rare: **58 in 100,000 babies.**

Long-term risks to baby after birth

For women/birthing people that have a caesarean birth,
18 in 1000 children will develop childhood asthma.

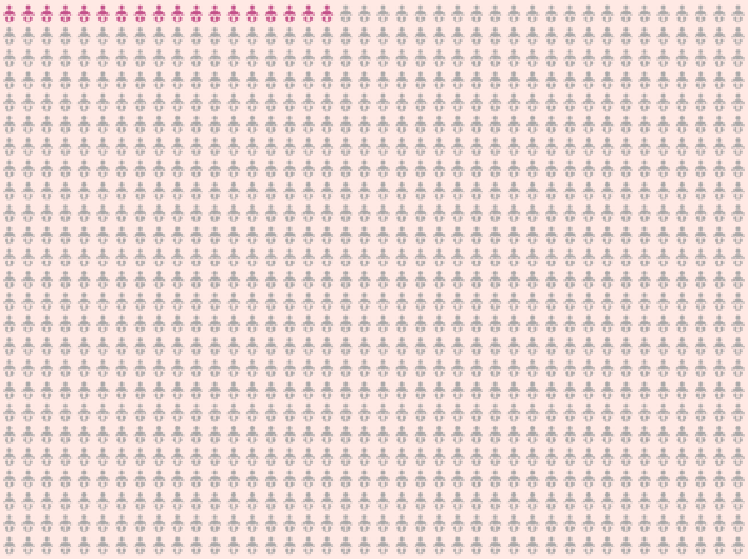

FUNDED BY NIHR | National Institute for Health and Care Research

UNIVERSITY OF LIVERPOOL

NHS Liverpool Women's NHS Foundation Trust

Recovering after a caesarean birth

Birth Options

Bowels

It can take some time for your bowels to get back into a normal pattern, but this usually happens by about 1 week after birth. Some women will experience painful trapped wind and constipation, which you can take medication for.

Vaginal bleeding

- Vaginal bleeding after caesarean is normal; use period pads (not tampons) to avoid infection.
- You can expect bleeding as heavy as a period which will slowly tail off until it stops around two weeks.

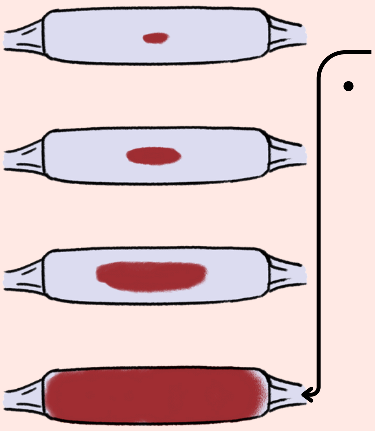

- If you are **soaking through pads** please see your midwife.
- If your bleeding slows down and then becomes heavier again speak to your midwife or doctor. It may be a sign of an infection.

Managing pain

Pain management both whilst in hospital and at home

You have had a big operation. It is important to take painkillers regularly to keep the pain under control. You will usually be offered paracetamol, dihydrocodeine and an anti-inflammatory (ibuprofen/naproxen/diclofenac).
In the hospital you may also be offered other drugs if needed, for example, morphine.

Caesarean scar pain in the short and long term

The scar (usually horizontal below the bikini line) will fade with time. Discomfort may last several days to weeks; but can continue for several months.

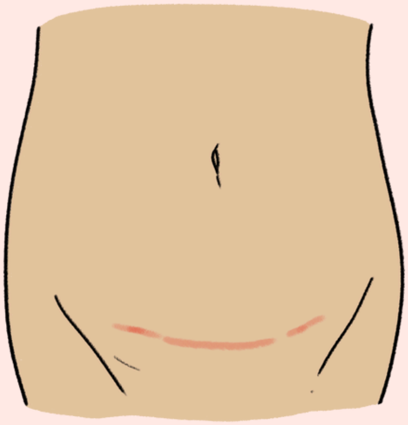

What to expect following a caesarean birth

Birth Options

Pain

You will experience pain after your caesarean. This will improve over time.
Each woman is different but pain should begin to get better by 2 weeks, although your cut is likely to remain tender for longer than this.

Feeding your baby

Both breastfeeding and formula feeding can be supported around the time of birth.
In theatre you or your birth partner may wish to have skin to skin.

Food and Drink

You will be able to eat and drink soon after birth once you feel up to it.

Moving to the ward

You will be moved from the recovery room to the ward within a couple of hours of your baby being born depending on how you are.

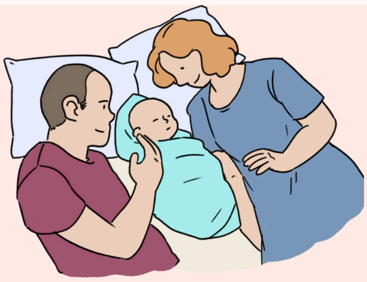

Walking

You will not be able to walk, shower, or have your catheter removed until you can feel and move your legs properly.

Catheter removal

Typically, bladder function returns in a few hours. Your catheter will usually be removed after 12 hours, once you are able to walk around. There may be some pain/discomfort when weeing (or when bladder is full) as you recover from the caesarean. Sometimes the bladder needs to rest more and you may need another catheter. You may need to go home with this for a few days.

FUNDED BY NIHR | National Institute for Health and Care Research
UNIVERSITY OF LIVERPOOL
NHS Liverpool Women's NHS Foundation Trust

What to expect following a caesarean birth

Birth Options

Wound Dressing

You will be advised to leave your dressing on for at least 24 hours.

Length of time in hospital

Many women will go home approximately 24 hours after a planned caesarean however some will stay longer.

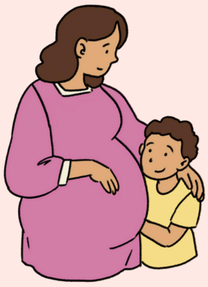

Prepare for your total hospital stay to be about 4 days.

Exercise and heavy lifting

We would advise no heavy lifting for 4-6 weeks.

Your body needs time to heal. You should be guided by your body in terms of exercise. It may be best to wait 6 weeks to resume high-impact exercise.

Sexual Activity

You should be guided by your body in terms of when you are ready to resume sexual activity.

Driving

You will be advised not to drive until you can do an emergency stop. This is often 4-6 weeks, but may be sooner. You are advised to contact your insurance company.

Blood thinning medication to reduce the risk of blood clots in legs and lungs

You will have an assessment of your risk of clots in your legs and lungs (deep vein thrombosis/pulmonary embolism) at the time of your caesarean.

Many women will be offered blood thinners as injections to reduce this risk. These can be taken at home for 10 days – 6 weeks.

FUNDED BY NIHR | National Institute for Health and Care Research
UNIVERSITY OF LIVERPOOL
NHS Liverpool Women's NHS Foundation Trust

Future pregnancies following a caesarean birth

Birth Options

The effects of caesarean birth on future pregnancies

Low-lying placenta (placenta praevia)

Where the placenta blocks the exit of the womb.

9 in 1000 women/birthing people

This increases with the number of caesarean births you have.

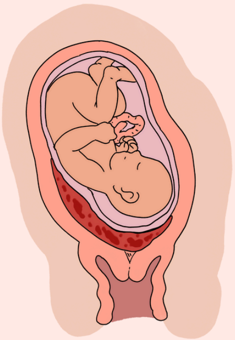

Invasive placenta (placenta accreta)

The placenta invades the wall of the womb. *This increases with the number of caesarean births you have.*

1 in 1000

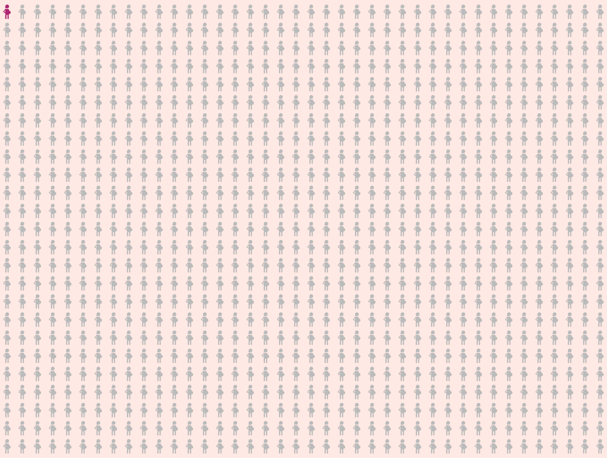

Stillbirth

Stillbirth after 39 weeks is uncommon, but may be increased in pregnancies after caesarean birth.

The *evidence is unclear.*

Womb (uterine) rupture

A hole forms in the womb.

2 in 1000 women/birthing people

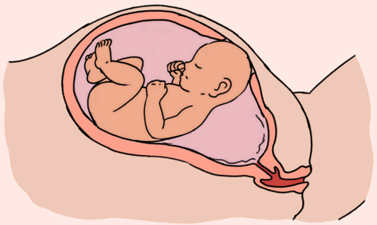

Future birth options

Choosing between a repeat caesarean birth or a vaginal birth after a caesarean birth (VBAC) may be an option. This will depend on the reason for your previous caesarean birth and your next pregnancy.

FUNDED BY NIHR | National Institute for Health and Care Research
UNIVERSITY OF LIVERPOOL
NHS Liverpool Women's NHS Foundation Trust

WHERE WE GOT THIS INFORMATION FROM

Why a caesarean birth was offered

- This information will be obtained from your medical records.

How common caesarean birth is

- NHS Maternity Statistics, England, 2023-24. last accessed 10/03/25. Available from: https://digital.nhs.uk/data-and-information/publications/statistical/nhs-maternity-statistics/2023-24/births

Were there any other realistic options for you for the birth?

- This information will be obtained from your medical records.

Were there any other realistic options for your baby for the birth

- This information will be obtained from your medical records.

How the caesarean birth went.

- This information will be obtained from your medical records.

Anaesthetic information

- This information will be obtained from your medical records.

Any complications at the time of the operation for you

- This information will be obtained from your medical records.

Any complications at the time of the operation for your baby

- This information will be obtained from your medical records.

Risks following the operation

- Royal College of Obstetricians and Gynaecologists. Planned Caesarean Birth: Consent Advice No. 14 August 2022 Minor Update November 2024.
- Larsson C, Källen K, Andolf E. Cesarean section and risk of pelvic organ prolapse: a nested case-control study. Am J Obstet Gynecol. 2009 Mar;200(3):243.e1-4. doi: 10.1016/j.ajog.2008.11.028.
- Rowlands, I.J., Redshaw, M. Mode of birth and women's psychological and physical wellbeing in the postnatal period. BMC Pregnancy Childbirth 12, 138 (2012). https://doi.org/10.1186/1471-2393-12-138.
- Reducing the Risk of Thrombosis and Embolism during Pregnancy and the Puerperium (Green-top Guideline No. 37a). Accessed 10/03/2025. Available from: https://www.rcog.org.uk/guidance/browse-all-guidance/green-top-guidelines/reducing-the-risk-of-thrombosis-and-embolism-during-pregnancy-and-the-puerperium-green-top-guideline-no-37a/.
- Herstad L, Klungsøyr K, Skjærven R, Tanbo T, Forsén L, Åbyholm T, Vangen S. Elective cesarean section or not? Maternal age and risk of adverse outcomes at term: a population-based registry study of low-risk primiparous women. BMC Pregnancy Childbirth. 2016 Aug 17;16:230. DOI: 10.1186/s12884-016-1028-3.

Recovering after a caesarean birth

- Caesarean birth. London: National Institute for Health and Care Excellence (NICE); 2024 Jan 30. PMID: 33877751. Available from: https://www.nice.org.uk/guidance/ng192/chapter/Recommendations.
- Postnatal Care. London: National Institute for Health and Care Excellence (NICE); 2021 April 20. Available from: https://www.nice.org.uk/guidance/ng194

FUNDED BY NIHR | National Institute for Health and Care Research

University of Liverpool

NHS Liverpool Women's NHS Foundation Trust

Birth Options

What to expect following a caesarean birth

- Caesarean birth. London: National Institute for Health and Care Excellence (NICE); 2024 Jan 30. PMID: 33877751. Available from: https://www.nice.org.uk/guidance/ng192/chapter/Recommendations.
- Postnatal Care. London: National Institute for Health and Care Excellence (NICE); 2021 April 20. Available from: https://www.nice.org.uk/guidance/ng194
- NHS Maternity Statistics, England, 2023-24. last accessed 10/03/25. Available from: https://digital.nhs.uk/data-and-information/publications/statistical/nhs-maternity-statistics/2023-24/births
- Reducing the Risk of Thrombosis and Embolism during Pregnancy and the Puerperium (Green-top Guideline No. 37a). Accessed 10/03/2025. Available from: https://www.rcog.org.uk/guidance/browse-all-guidance/green-top-guidelines/reducing-the-risk-of-thrombosis-and-embolism-during-pregnancy-and-the-puerperium-green-top-guideline-no-37a/.

Future pregnancies following a caesarean birth

- Caesarean birth. London: National Institute for Health and Care Excellence (NICE); 2024 Jan 30. PMID: 33877751. Available from:https://gbr01.safelinks.protection.outlook.com/?url=https%3A%2F%2Fwww.nice.org.uk%2Fguidance%2Fng192%2Fchapter%2FRecommendations&data=05%7C02%7Cmahmoud.aljubeh%40lwh.nhs.uk%7C3843a0ca508a4072072e08dd692e5ca2%7C473870bd20104a57ba6ddb4e47cf3d8b%7C0%7C0%7C638782370042525968%7CUnknown%7CTWFpbGZsb3d8eyJFbXB0eU1hcGkiOnRydWUsIlYiOiIwLjAuMDAwMCIsIlAiOiJXaW4zMiIsIkFOIjoiTWFpbCIsIldUIjoyfQ%3D%3D%7C0%7C%7C%7C&sdata=VWIaTW8HtlIPd%2FOFzenlUxt1YECIr4RTIGij0bR%2FmXE%3D&reserved=0.
- Gurol-Urganci I, Cromwell DA, Edozien LC, Smith GC, Onwere C, Mahmood TA, Templeton A, van der Meulen JH. Risk of placenta previa in second birth after first birth cesarean section: a population-based study and meta-analysis. BMC Pregnancy Childbirth. 2011 Nov 21;11:95. doi: 10.1186/1471-2393-11-95.
- Royal College of Obstetricians and Gynaecologists. Planned Caesarean Birth: Consent Advice No. 14 August 2022 Minor Update November 2024.
- Birth after Previous Caesarean Birth (Green-top Guideline No. 45) | RCOG Accessed: 10/03/2025. Available from: https://www.rcog.org.uk/guidance/browse-all-guidance/green-top-guidelines/birth-after-previous-caesarean-birth-green-top-guideline-no-45/.

How we present information about risk

**Very common**	**1 in 1 to 1 in 10**
**Common**	**Less than 1 in 10 to 1 in 100**
**Uncommon**	**Less than 1 in 100 to 1 in 1000**
**Rare**	**Less than 1 in 1000 to 1 in 10,000**
**Very rare**	**Less than 1 in 10,000**

FUNDED BY NIHR | National Institute for Health and Care Research

UNIVERSITY OF LIVERPOOL

NHS Liverpool Women's NHS Foundation Trust
